# Chemical modification patterns for microRNA therapeutic mimics: a structure-activity relationship (SAR) case-study on miR-200c

**DOI:** 10.1093/nar/gkae141

**Published:** 2024-02-29

**Authors:** Marion Garreau, Julie Weidner, Russell Hamilton, Ewa Kolosionek, Naoko Toki, Kathrin Stavenhagen, Clément Paris, Alessandro Bonetti, Werngard Czechtizky, Felix Gnerlich, Anna Rydzik

**Affiliations:** Medicinal Chemistry, Research & Early Development, Respiratory & Immunology, BioPharmaceutical R&D, AstraZeneca, Gothenburg, Sweden; Translational Science Experimental Medicine, Research & Early Development, Respiratory & Immunology, BioPharmaceutical R&D, AstraZeneca, Gothenburg, Sweden; Translational Science Experimental Medicine, Research & Early Development, Respiratory & Immunology, BioPharmaceutical R&D, AstraZeneca, Cambridge, UK; Bioscience COPD/IPF, Research & Early Development, Respiratory & Immunology, BioPharmaceutical R&D, AstraZeneca, Gothenburg, Sweden; Translational Genomics, Discovery Biology, Discovery Sciences, R&D, AstraZeneca, Gothenburg, Sweden; Medicinal Chemistry, Research & Early Development, Respiratory & Immunology, BioPharmaceutical R&D, AstraZeneca, Gothenburg, Sweden; Medicinal Chemistry, Research & Early Development, Respiratory & Immunology, BioPharmaceutical R&D, AstraZeneca, Gothenburg, Sweden; Translational Genomics, Discovery Biology, Discovery Sciences, R&D, AstraZeneca, Gothenburg, Sweden; Medicinal Chemistry, Research & Early Development, Respiratory & Immunology, BioPharmaceutical R&D, AstraZeneca, Gothenburg, Sweden; Medicinal Chemistry, Research & Early Development, Respiratory & Immunology, BioPharmaceutical R&D, AstraZeneca, Gothenburg, Sweden; Medicinal Chemistry, Research & Early Development, Respiratory & Immunology, BioPharmaceutical R&D, AstraZeneca, Gothenburg, Sweden

## Abstract

microRNA (miRNA) mimics are an emerging class of oligonucleotide therapeutics, with a few compounds already in clinical stages. Synthetic miRNAs are able to restore downregulated levels of intrinsic miRNAs, allowing for parallel regulation of multiple genes involved in a particular disease. In this work, we examined the influence of chemical modifications patterns in miR-200c mimics, assessing the regulation of a selection of target messenger RNAs (mRNA) and, subsequently, of the whole transcriptome in A549 cells. We have probed 37 mimics and provided an initial set of instructions for designing miRNA mimics with potency and selectivity similar to an unmodified miRNA duplex. Additionally, we have examined the stability of selected mimics in serum. Finally, the selected two modification patterns were translated to two other miRNAs, miR-34a and miR-155. To differing degrees, these designs acted on target mRNAs in a similar manner to the unmodified mimic. Here, for the first time, we describe a structured overview of ‘miRNA mimics modification templates’ that are chemically stabilised and optimised for use in an *in vitro* set up and highlight the need of further sequence specific optimization when mimics are to be used beyond *in vitro* tool experiments.

## Graphical Abstract

**Graphical Abstract:**
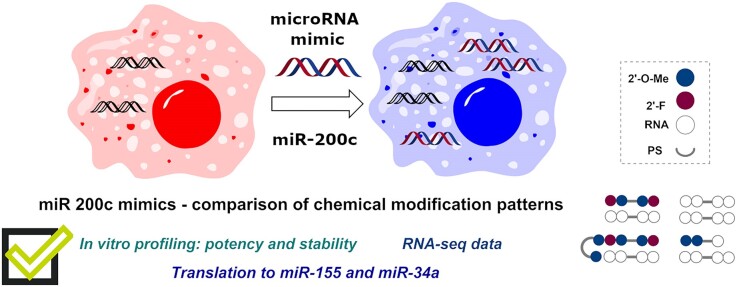

## Introduction

In recent years, oligonucleotide therapeutics have gained considerable attention, offering an expansion of the druggable target space (1–3). Among the diverse classes of oligonucleotides, microRNAs (miRNAs) have been widely used for diagnostic purposes and only recently emerged as a promising therapeutic approach (4–7). miRNAs are a class of endogenously expressed small non-coding double-stranded RNAs that are approximatively 17–25 nucleotides long in their mature form (8–10). miRNA mediated regulation is an intrinsic cellular mechanism accomplished through RNA interference (RNAi) (10,11). In the RNAi pathway, a mature miRNA duplex is incorporated into the RNA-induced silencing complex (RISC) and specifically recognized by an Argonaut protein (Ago). The miRNA duplex is functionally asymmetric and one of the strands (antisense or guide strand) is preferentially loaded into Ago, while the other strand (sense or passenger strand) is unwound from the duplex and discarded. The guide strand is then spatially arranged for binding to complementary mRNA sequences, leading to translational repression. miRNA binding motifs are often found in the 3′-UTR of target mRNAs, however in mammalian cells, there is seldom full complementary to the whole guide strand sequence. The principal interaction occurs at the seed region (nt 2–8) of the miRNA guide strand, but various other positions can also be involved resulting in many possible mRNA targets for one miRNA sequence. On the contrary, small interfering RNAs (siRNAs) are fully complementary to mRNA and optimised for specificity to one target.

miRNAs play a major role in regulating gene expression, therefore any disease driven dysregulation of miRNA levels can lead to abnormal expression of a whole gene network and, consequently, have a profound effect on cell homeostasis. For this reason, miRNA targeted approaches offer the unique opportunity to regulate multiple targets with a single drug. In the case of downregulation of intrinsic miRNA levels, a miRNA replacement therapy could be achieved using miRNA mimics (1,2,4,12). Mimics are synthetic versions of endogenous miRNAs designed with the goal to restore the level and activity of endogenous miRNAs. When considering the design of those molecules, miRNA mimics face pharmacological challenges common for all the oligonucleotide therapeutics. For instance, unmodified RNA is rapidly degraded *in vivo*. Introduction of chemical modifications is therefore crucial for oligonucleotide therapeutics of clinical use. Furthermore, modifications can have a profound effect on potency, selectivity and pharmacokinetic properties of oligonucleotides (1,3,13,14).

miRNA mimics are still in the early stages of clinical development, with only three candidates in clinical trials (15–17). Starting points for developing synthetic miRNA mimics are currently not well defined and chemical modification patterns are often covered by intellectual property (18). Therefore, in this work, we explored the structure-activity relationship for common oligonucleotide chemical modifications in the context of miRNA mimics. We envisaged providing a ‘general modification template’ that would introduce chemical stabilisation into the mimic, while maintaining the potency and selectivity of the unmodified miRNA. We hope that this work could serve as a foundation for developing a variety of miRNA mimic tool compounds and lay a path for future miRNA mimics in drug discovery.

## Materials and methods

### Oligonucleotide synthesis and purification

Oligonucleotides were synthesised on solid phase using a K&A Laborgeräte H8 SE oligonucleotide synthesizer. The synthesis was performed according to standard procedures on a 1 μmol scale using 0.1 M in acetonitrile phosphoramidites (Sigma-Aldrich) and CUTAG CPG support (Sigma-Aldrich). For RNA synthesis, preloaded CPG columns were purchased from Link. Detritylation was achieved by using 3% dichloroacetic acid in dichloromethane (Sigma-Aldrich). Activator 42**^®^** (Sigma-Aldrich) was used for coupling steps. Oxidation conditions were pyridine/water/iodine 9/1/12.7 (v/v/w). Thiolation was performed using Xanthane hydride (0.2 M) (TCI chemicals). Sequences were synthesized with removal of the final DMT group. At the end of the synthesis, cyanoethyl deprotection was performed on resin with diethylamine/acetonitrile (20% v/v) (Sigma-Aldrich). Phosphorylation at the 5′ position was achieved using the reagent 2-[2-(4,4′-dimethoxytrityloxy)ethylsulphonyl]ethyl-[(2-cyanoethyl)-(*N*,*N*-diisopropyl)]-phosphoramidite (Link, Item Number: 2101). According to the manufacturer protocol, it was added at the last coupling step in the same conditions.

After synthesis, the oligonucleotides were cleaved from the resin and deprotected for 15 h at 55°C using aqueous ammonia (35%) (1 ml/1 μmol). The crude oligonucleotide was then evaporated to dryness in a Speedvac vacuum concentrator. Different cleavage conditions were employed for RNA synthesis: after the same resin flushing with ammonia, 95% EtOH (20% of the total volume) was added, and the resulting solution placed 5 h at 55°C. After Speedvac vacuum concentration, the resulting solid was dissolved in DMSO (125 μl/1 μmol) and subjected to triethylamine trihydrofluoride (TEA*3HF) (125 μl/1 μmol) treatment for 2.5 hours at 65°C. *n*-butanol (1 ml/1 μmol) was added, and the vial cooled at –20°C. The precipitate was collected by centrifugation and dried in a Speedvac vacuum concentrator.

Purification of the crude oligonucleotides was performed by RP HPLC and pure fractions were freeze dried. An XBridge C18, 5μm 19 × 150mm column was used with mobile phases A: 60 mM DBuAA (H_2_O/ACN 95/5) pH7 and B: 60 mM DBuAA in ACN. The gradient was adapted to each sequence. Precipitation was achieved by addition of a 3M NaOAc solution at pH 5.2 (10% of volume) to an aqueous solution of the oligonucleotides, subsequent 95% EtOH addition (80% of total volume) and placing the mixture at -20°C. The precipitate was collected by centrifugation, dried in a Speedvac vacuum concentrator and dissolved in water for subsequent use.

### Oligonucleotide characterization

The concentration was determined by absorbance measurements using a NanoDrop™ 2000c Spectrophotometer (ThermoFisher Scientific, Sweden). Purity determination and high-resolution mass analysis were performed on a Waters BioAccord LC-MS equipped with TUV and ACQUITY RDa Detectors (Waters). The column was XBridge BEH C18, 3.5 μm 2.1 × 150 mm (Waters) with mobile phases A: 10 mM TBuAA in MeCN/water 1:9; and B: MeCN. The gradient was 20% to 70% B over 8 min. The purity was estimated at 260 nm. The spectrums and mass data are included in the supplementary data (SI Section 10: LCMS Data).

### Duplex annealing

Duplexes were annealed by mixing equimolar solutions of guide and passenger strands. To 20 μl of each strand at 500 μM was added 10 μl of PBS 10×, affording 0.01 μmol of each duplex at 200 μM. The mixture was incubated for 10 min at 85°C and slowly cooled to RT for 2 h. Purity was evaluated by gel electrophoresis using 20% TBE Gels 1.0MM 10W (ThermoFisher, Sweden) in TBE buffer. Loading was done with Novex**^®^** high density TBE sample buffer 5× (ThermoFisher, Sweden). Running conditions were 180 V and 350 W for 50 min. Staining was achieved using SYBR**^®^** Gold Nucleic Acid Gel Stain according to manufacturer's protocol (ThermoFisher Scientific, Sweden).

### Cell culture

A549 cells (ATCC #CCL-185) were cultured in DMEM medium (Gibco, DMEM, low glucose, GlutaMAX™ Supplement, pyruvate, Catalog number: 21885025) supplemented with 10% heat inactivated Fetal Bovine Serum (FBS), Penicillin (100 U/ml) and Streptomycin (100 U/ml) in a humidified incubator at 37°C; 5% CO_2_.

### Transfection procedure

The following procedure is given for 24-well plates and was scaled accordingly when used with 48-well plates. A549 cells were seeded on a 24-well plate at a density of 50 000 cells/well in complete DMEM. After 24 h of incubation, the medium was replaced with DMEM without antibiotics, but with heat inactivated FBS. Transfection was performed with miRNA mimics using Lipofectamine RNAiMAX® following the protocol from manufacturer (ThermoFisher, Sweden). miRNA mimics were used at a final concentration of 10 nM for miR-200c and miR-34a and 30 nM for miR-155 per transfection. The cells were incubated for 24 hours at 37°C in a 5% CO_2_ incubator before being harvested. Controls included media only, hsa-miR-200c-3p miRIDIAN® microRNA Mimic (Dharmacon, MIMAT0000617), hsa-miR-34a-3p miRVana® microRNA Mimic (ThermoFisher, Assay ID: MC13089), hsa-miR-155-5p miRVana® microRNA Mimic (ThermoFisher, Assay ID: MC28440), miRIDIAN® microRNA Mimic Transfection Control with Dy547 (Dharmacon, CP-004500-01-05) and lipofectamine alone (mock). Each experiment was done in at least technical duplicates and biological triplicates. RT-qPCR data from controls are presented in Supplementary Figure S5 and S6 but were not included in each heatmap for clarity.

### RNA extraction and TaqMan assay

Cells were washed with twice with 1× phosphate buffered saline solution (ThermoFisher Scientific) prior to the addition of 200 μl TRIzol Reagent (Invitrogen) per well. Samples were then snap-frozen at -80°C until processing occurred. Total RNA was extracted a Zymo RNeasy 96 kit (Zymo Research Catalog R2056) following the manufacturer's protocols. The yield and quality of extracted RNA was determined by a NanoDrop™ 8000 Spectrophotometer (ThermoFisher Scientific, Sweden). Total RNA (125 ng) was reverse-transcribed (RT) into cDNA using the High-Capacity cDNA Reverse Transcription Kit (Catalog 4368814; ThermoFisher Scientific) according to the supplier's instructions. Real-time qPCR was performed using TaqMan Fast Advanced Master Mix (Applied Biosystems, Sweden), 10 μg cDNA and 50 mM of the following primers: ZEB1 (Assay ID: Hs00232783), ZEB2 (Assay ID: Hs00207691), FN1 (Assay ID: Hs01549976), MSN (Assay ID: Hs00741306), ATRX (Assay ID: Hs00997529), ETS1 (Assay ID: Hs00428293_m1), HIF1a (Assay ID: Hs00153153_m1), SMAD5 (Assay ID: Hs00195437_m1), HDAC1 (Assay ID: Hs00606262_g1), PREB (Assay ID: Hs00936690_g1) and SIRT1 (Assay ID: Hs01009006_m1).

Samples were run on a QuantStudio 7 Flex thermocyler (ThermoFisher Scientific) using the following protocol: 95°C for 10 min (1 cycle), 95°C for 30 s, 58°C for 30 s, and 72°C for 30 s (40 cycles). The comparative 2^−ΔΔCt^ method was used to analyse mRNA fold changes between control with media only and miRNA mimics treatment. This was calculated as a ratio = 2-(ΔCt treatment − ΔCt control), where Ct is the cycle threshhold and ΔCt (Ct target gene − Ct reference gene) is the Ct value normalized to the reference gene (HPRT1; Assay ID Hs02800695) obtained for the same cDNA samples. Each experiment was performed in biological triplicate and at least experimental duplicates.

### Luciferase dual reporter assay

#### Screening

Hepa 1–6 cells were obtained from ATCC (ATCC in partnership with LGC Standards, Wesel, Germany, cat. #ATCC-CRL-1830) and cultured in DMEM (#30–2002, ATCC/LGC, Wesel, Germany). The culture medium was supplemented to contain 10% fetal calf serum (1248D, Biochrom GmbH, Berlin, Germany), and 100 U/ml penicillin/100 μg/ml Streptomycin (A2213, Biochrom GmbH, Berlin, Germany) at 37°C in an atmosphere with 5% CO_2_ in a humidified incubator. For co-transfection of Hepa1-6 cells with psiCheck-2 (containing the sequence of interest, see SI for details) and Oligos, cells were seeded at a density of 20000 cells/well into 96-well tissue culture plates (#655180, GBO, Germany).

#### Transfections

In Hepa 1–6 cells, co-transfection of plasmid and oligos was carried out with Lipofectamine2000 (Invitrogen/Life Technologies, Karlsruhe, Germany) according to manufacturer's instructions for reverse transfection with 0.5 μl Lipofectamine2000 and a constant concentration of 50 ng of plasmid per well. The dual dose screen was performed with oligos in quadruplicates at 10 nM and 0.5 nM, respectively, with one siRNA targeting RLuc (serving as an internal positive control), a positive control, an inactive control siRNA directed to F-Luc and a mock transfection. DualGlo Assay (Promega) was performed according to the manufacturer's protocol. Briefly, after 24h of incubation with plasmid and oligos, medium was removed and cells were lysed in 150 μl lysis reagent (1 volume Luciferase reagent, DualGlo, Promega, 1 volume cell culture medium) and then incubated at RT in the dark for 30 min, followed by readout in a luminescence counter for F-Luc signal. After addition of 75 μl Stop-&-Glo Buffer containing Stop-&-Glo reagent, plates were again incubated at RT in the dark for 10 min and again measured in the luminescence counter for R-Luc signal. Luminescence was read using 1420 Luminescence Counter (WALLAC VICTOR Light, Perkin Elmer, Rodgau-Jügesheim, Germany).

For each well, the R-Luc signal was normalized to the respective F-Luc signal to correct for plasmid loading and cell count. The activity of a given oligo was expressed as percent relative R-Luc activity (normalized to F-Luc) in treated cells, relative to the negative control signals (normalized to F-Luc) averaged across negative control wells.

### Statistics

Statistics are presented as bar graphs (Supplementary Figure S7–S9, S11) and in Tables S3-5, S7 and S8 that correspond to the heat graphs presented (Figure 2B, 3B and 4B), the luciferase assay (Figure 5) and the translated mimic designs (Figure 10). To compare knock down efficiency of different series of mimics, ordinary one-way ANOVA followed by Dunnett's multiple comparison test was performed. In general, comparisons were done with each mimic to untreated controls (shown in Tables S3–S5, S7 and S8) or each mimic to the WT miRNA design (Tables S3–S5 & Supplementary Figure S7–S9). Statistical analyses were performed using GraphPad Prism 9 software (GraphPad Software Inc., San Diego, CA, USA). *P*< 0.05 was considered to be significant.

### Stability assays

#### Stability in FBS

20 μl of 5 μM miRNA mimic was mixed with 80 μl of FBS (Gibco #10270) and incubated at 37°C. Samples were taken at 0 and 24 h time points. 20 pmol of each sample was loaded in Novex™ Hi-Density TBE Sample buffer (Thermofisher) and analyzed on Novex™ 20% TBE Gel. The gel was run for 50 min at 250 V in Novex™ TBE Running Buffer (Thermofisher). After electrophoresis, gels were incubated in Sybr™ Gold Nucleic Acid Stain (Thermofisher) for 10 min and subsequently washed twice with water. Imaging was done with UV transillumination using the GelDocGo imaging system (Biorad).

#### Stability in rat serum

RNA stability was tested in 80% rat serum over the course of 24 h and analyzed by anion-exchange chromatography under denaturing conditions. 30 μl of 200 μM miRNA in 80% rat serum (Wistar rats, male) were incubated in the autosampler of a Vanquish UHPLC (Thermo Fisher Scientific) at 37°C. Directly at *t* = 0, after 0.5, 4 and 24 h, 2 μl were injected onto a DNAPac PA200 analytical column (4 μm particle size, 4.6 × 150 mm, Thermo Fisher Scientific). The column temperature was set to 65°C. Samples were eluted with a gradient of buffer A (20 mM sodium phosphate, pH 11 (Sigma Aldrich)) and buffer B (20 mM sodium phosphate, 1 M sodium bromide (Sigma Aldrich), pH 11), going from 35% B to 98% B in 8 min, followed by a wash at 98% B over 1.5 min and column equilibration at a flow rate of 1 ml/min. UV detection was performed at 260 nm.

### RNA sequencing

#### Total RNA isolation

Total RNA from cells was extracted using the RNAdvance Cell kit (Beckman Coulter). RNA extraction was performed using Biomek i7 robotic handler (Beckman Coulter) following manufacturer instructions. RNA quality of extracted RNA was assessed using Fragment Analyzer automated capillary electrophoresis system (Advanced Analytical). All samples had RNA Integrity Number (RIN) above 9 and therefore were used for following transcriptional analysis.

#### Library preparation and sequencing

55 ng of total RNA were used for RNA-seq library preparation using the KAPA mRNA HyperPrep Kit (Roche) following manufacturer's instruction (KAPA Biosystem, Illumina Platforms) proceeded by TECAN Fluent liquid handing automation system (Tecan). The library was amplified for 14 cycles and quantified using the Fragment Analyzer (Advanced Analytical) with the standard sensitivity NGS kit (Agilent technologies) followed by the library clean-up with 1x volume of AMPure XP beads (Beckman Coulter Life Sciences). All the libraries were pooled and diluted into 1.8 nM quantified using Qubit Fluorometer (ThermoFisher Scientific), and sequenced with paired-end, 100 cycles, using SP regent kit by Illumina Novaseq 6000.

#### Bioinformatics data processing

RNA-Seq raw FASTQ files were processed using the Nextflow workflow management system (based on rnaseq-nf-3.0.0) (19). Read quality was assessed using FastQC (v0.11.9, https://www.bioinformatics.babraham.ac.uk/projects/fastqc/), before aligning to the human reference genome (GRCh38, Ensembl v105) using Salmon (v1.7.0) (20). Alignment BAM files were sorted and indexed with SAMtools (v1.15) (21), and quality assessed using RSeQC (v4.0.0) (22) and Qualimap (v2.2.2d) (23). Gene counts were quantified from the alignment files using Salmon (v1.7.0) (20). A summary for raw read, alignment, gene biotype, sample similarity, and strand-specificity checks (MultiQC (v1.12) (24). A PCA was constructed using variance stabilized transformation (vst) counts calculated in DESeq2 (v1.34.0) (25) to assess sample clustering and identify outliers (Supplementary Figure S12). All libraries were used in the subsequent differential expression analyses. Differential expression tests were performed with edgeR (v3.36.0) (26) and within R (v4.1.1, http://www.R-project.org/). The edgeR functions glmQLFit and glmQLFTest were used and all contrasts were performed using a false discovery rate cut off of 0.05. Threshold value was set to l2fc (Log_2_ Fold Change) > 1.3 and specified in the appropriate figures and tables. Gene targets of hsa-miR-200c-3p, hsa-miR-34a-5p, and hsa-miR-155-5p were identified using multiMiR (v1.16.0) and intersected with the differentially expressed genes for each contrast. Plots were generated in R (v4.1.1) using ggplot2 (v3.3.5). 3′UTR coordinates as GenomicRanges were extracted from TxDb.Hsapiens.UCSC.hg38.knownGene (v3.14.0), and the 3′UTR sequences retrieved from BSgenome.Hsapiens.UCSC.hg38 (v1.4.4). Counts of seed and passenger matches were made using vmatchPattern (max.mismatch = 0; BioStrings v2.62.0), and intersected with differential expression results for each comparison made (log Fold change cut offs ± 1.3). Seed and passenger sequences used were: hsa-miR-200c-3p (seed:AAUACUG; passenger:UCGUCAU).

## Results

### Model system selection and design

As our model miRNA, we chose hsa-miR-200c-3p (miR-200c), which has been widely investigated in cancer, particularly in the context of epithelial-mesenchymal transition (EMT) (27,28). With clear evidence of the reversible nature of EMT, the restoration of miR-200c levels appears to be an attractive therapeutic approach and it has been explored for several cancer types (29), particularly for non-small cell lung cancer (NSCLC) (30–32).

For the initial screen of miRNA mimics, we aimed to utilise a set of genes directly targeted by miR-200c and assess the effect of each mimic on the mRNAs by a RT-qPCR assay. To select gene targets, we queried three miRNA databases: miRDB (http://mirdb.org) (33), miRTarbase (http://miRTarBase.cuhk.edu.cn/) (34) and miRNET (https://www.mirnet.ca) (35). For miRNET, ‘lung’ was specifically examined as a target organ, as we considered the role of miR-200c in lung cancer of particular interest. Hit lists were then compared via Venny (http://bioinfogp.cnb.csic.es/tools/venny/) and the 95 mRNAs common to all databases were further analysed (Supplementary Figure S1). We then compared the available evidence behind target selection and prioritised targets confirmed by RT-qPCR and pulldown experiments over those with evidence only from Next-generation Sequencing (NGS) and microarrays. mRNA target selection was subsequently narrowed down by including expression levels in A549 cells (EBI Expression Atlas: https://www.ebi.ac.uk/gxa/experiments/E-MTAB-4729/Results, Supplementary Figure S2), which is a lung cancer cell line. Finally, we compared interaction networks of the targets using StringDB (https://string-db.org/, Supplementary Figure S3) (36). Following further validation using published literature, we selected five target genes for this study: ZEB1, ZEB2, FN1, MSN and ATRX (Table 1). ZEB1 and ZEB2 were of interest due to their involvement in EMT and wide correlation to miR-200c regulation (37,38). FN1 and MSN are aberrantly expressed in cancer cells and play a role in maintaining their epithelial character (39). ATRX has also been reported as a miR-200c family target (30).

**Table 1.** Direct target genes selected in this study

Target gene	Full name	References
**ZEB1**	Zinc finger E-box-binding homeobox 1	(37,38,40)
**ZEB2**	Zinc finger E-box-binding homeobox 2	(37,38,40,41)
**FN1**	Fibronectin	(39)
**MSN**	Moesin	(39)
**ATRX**	Alpha thalassemia/mental retardation syndrome X-linked	(30)

In the experimental set up we have used A549 cells, which have intrinsically low levels of miR-200c, allowing a better activity window for the mimics (Supplementary Table S1). To determine the required mimic concentration, we transfected the cells with 5, 10, 30 or 50 nM of a commercial mimic (miRIDIAN®), and analysed mRNA levels 24 h post-transfection by RT-qPCR (Supplementary Figure S4). We observed knockdown (KD) for all selected target mRNAs, with ZEB1 and ZEB2 being the most responsive, and ATRX the least responsive. 10 nM of the mimic was chosen as an optimal concentration for further experiments. Under those conditions, average knockdown was measured to be: 60% for ZEB1, 78% for ZEB2, 48% for FN1, 55% for MSN and 25% on ATRX (Supplementary Figure S4). The differences in KD intensities could arise from the abundance of miR-200c binding sites within the 3′-UTRs of the chosen targets. If both ZEB1 and ZEB2 possess six predicted binding sites, FN1 and MSN have only three each (42). The least responsive target, ATRX, presents only two binding sites (30). This occurrence could explain the variable effect across the studied mRNAs. The system was further validated with three commercial mimics together with the unmodified duplex (**M1**). Efficient knockdown was measured on the five targeted mRNAs with relatively similar profiles for all (Supplementary Figure S5).

### Chemical modifications in miRNA mimics

Chemical modifications are essential in RNA therapeutics. They can improve the pharmacokinetics (PK) profile, including half-life, but also influence potency and selectivity among others (1,3,13,14,18). In this work we aimed to use common oligonucleotide modifications to improve potency and modulate selectivity of miRNA mimics. Our goal is to achieve a mimic with a similar targeting profile to an unmodified miRNA and to provide an initial design guide for synthetic miRNA mimics, to be used as *in vitro* tool compounds. *In vivo* stability, potency evaluation and ADME aspects were out of scope for this study.

**Figure 1.**
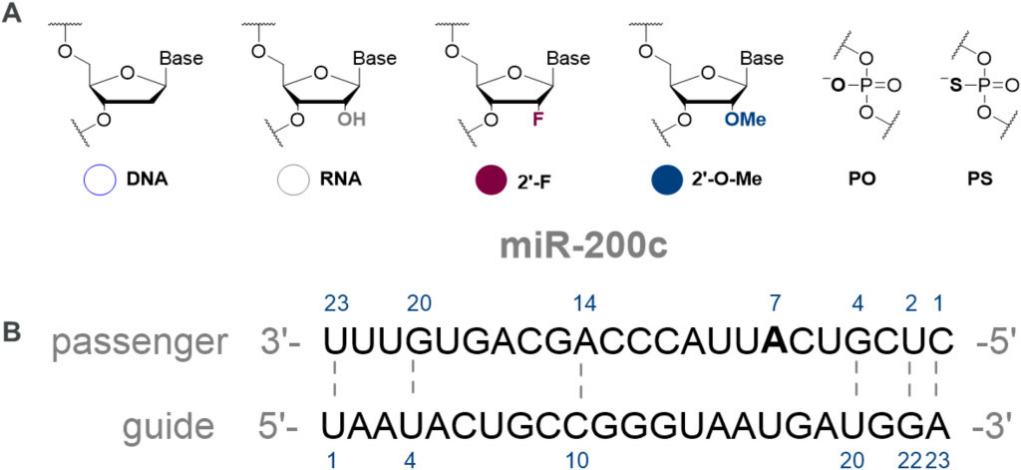
(**A**) Chemical modifications for miRNA mimics. (**B**) Reference sequence for the miR-200c mimics used in this study. Mismatches sites are indicated by numbered nucleotides and dashed lines.

Some of the common oligonucleotide chemical modifications include sugar and backbone functionalisation. For instance, replacing the 2′-hydroxy group of the ribose with fluoro (F) or methoxy (OMe) residues, can block nucleolytic degradation and increase duplex stability, leading to tighter target binding (14,43). In our design, apart from unmodified ribose sugar, we introduced 2′-F, 2′-OMe and 2′-deoxy (DNA) modifications (Figure 1). Furthermore, we studied the effect of replacing phosphodiester bonds by a phosphorothioate moiety. This modification improves stability from DNase cleavage, but also has a slightly destabilizing effect on a duplex (14,44). As most miRNA mimics are double-stranded structures, we also evaluated spatial design, for example duplex architecture, including mismatches, overhangs, and hairpin type designs.

### Iterative SAR case-study on miR-200c mimics

#### Series 1

The reference sequence for the unmodified miR-200c guide strand was sourced from miRbase (https://www.mirbase.org/cgi-bin/mirna_entry.pl?acc=MI0000650). A passenger strand was designed to be of the same length as the guide strand (Figure 1B). Appropriate mismatches were included in the positions 1, 2, 4, 14, 20 and 23, as guided by the pre-miRNA sequence. For enhanced duplex formation, a deletion in position 7 of the passenger strand, was replaced with a match (A–U). This was then used as the sequence template for subsequent mimic designs.

**Figure 2.**
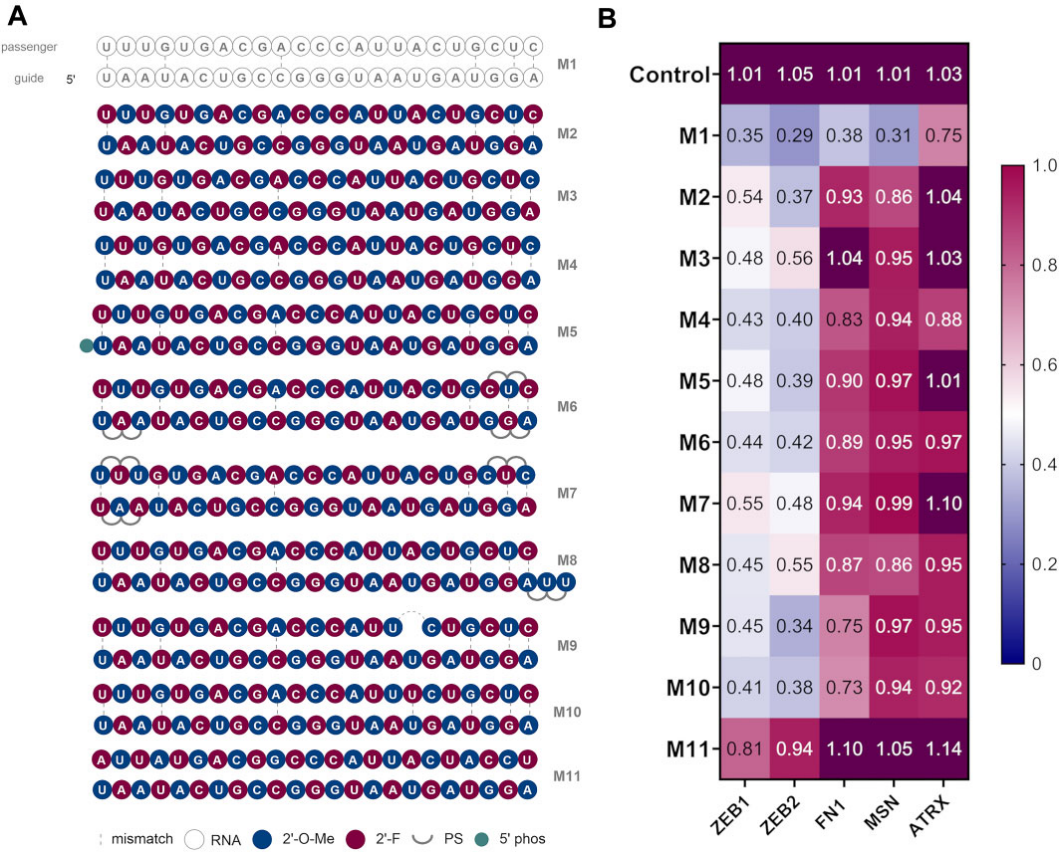
(**A**) Structures of first generation miR-200c mimics. (**B**) Knockdown (KD) data of first generation miR-200c mimics. A549 cells were transfected with 10 nM final concentration of mimics for 24 h then analysed by RT-qPCR (*n* = 3). Target mRNA expression was normalised to HPRT1 and baseline levels (control) were set to 1. The heat map indicates the average expression of each mRNA targeted by the different mimics (red = 1; blue = 0).

For our chemical modification study, we started with an alternating pattern consisting of 2′-F and 2′-OMe modified ribose moieties arranged one after another in both guide and passenger strands. In the initial design, the 5′-ribose of the guide strand was a 2′-OMe modified nucleoside and the passenger strand residues were arranged in the following manner so that 2′-OMe modified nucleotides of the guide strand were complementary to the 2′-F nucleotides in the passenger strand (Figure 2A, **M2**). This alternating pattern ensures optimal duplex conformation and is a widely used modification pattern in siRNA duplex designs (14,45). We explored further variations where the 5′ of the guide strand starts with 2′-F modified ribose (**M3**) and where 2′-OMe modifications in the guide are complementary to 2′-OMe in the passenger (**M4**). All three designs had very similar profiles when the KD of the five target genes were examined (Figure 2B, Table S3) and led to an efficient knockdown of ZEB1 and ZEB2 (50–60% observed) however, KD efficiency of the other targets was decreased. We based further designs on the pattern from **M2**, which was overall slightly more active than **M3** for most targets. Next, we investigated if introduction of a phosphate at the 5′ of the guide strand would lead to a change in activity (**M5**). The 5′-phosphate is a critical recognition element for the miRNA duplex - Ago protein binding, however in this setting no difference was observed between the phosphorylated (**M5**) and unphosphorylated (**M2**) design (Figure 2B). This was consistent with the current literature, where 5′ phosphorylation might have no major impact on siRNA activity *in vitro* (46).

Next, we introduced phosphorothioates (PS) at the ends of the duplex strands. This has been proven to be an effective strategy for siRNA design, providing additional stability towards exonucleases. In the **M6** and **M7** designs, six PS linkages were introduced at the different positions of the duplexes (Figure 2A). An improved activity was observed in case of **M6** for ZEB1, FN1 and ATRX comparted to **M7**. The addition of a 3′-UU overhang (**M8**) led to a comparable profile as **M6** and was not examined further (Figure 2).

miRNA duplexes are often not fully complementary and can contain several mismatches. For instance, the reported miR-200c duplex, apart from mismatches in position 1, 4, 10, 20, 22 and 23 of the guide strand, has a deletion in the passenger strand facing position 17 of the guide strand. We further investigated the role of single mismatches in mimic design (Figure 2A) and introduced the native deletion in mimic **M9** and a simple mismatch (U-U) in this position (**M10**). We have also tested a design where both strands are fully complementary to each other (**M11**). We observed no great difference in activity between designs **M9** and **M10**. Both mimics displayed a similar profile, with good KD of ZEB1 and ZEB2 and moderate to low activity on the other targets (Figure 2B). A fully complementary passenger strand (**M11**) led to a lower activity for ZEB1 and ZEB2 and no activity for FN1, MSN and ATRX, confirming the important role of mismatches in miRNA mimic design. Although the differences between activity and selectivity of **M2**, **M9** and **M10** were similar overall, **M10** demonstrated the best profile when all targets were considered. Therefore, for the next generation of mimics, we decided to introduce the U–U mismatch at positions 17 and 7 of the guide and passenger strands, respectively (Table S2).

This round of screening provided us with an optimised set of initial designs, where the KD of ZEB1 and ZEB2 was already comparable to the unmodified miR-200c duplex (**M1**). However, the inhibition of other targets remained to be optimised (Figure 2, Supplementary Figure S7, Table S3).

#### Series 2

**Figure 3.**
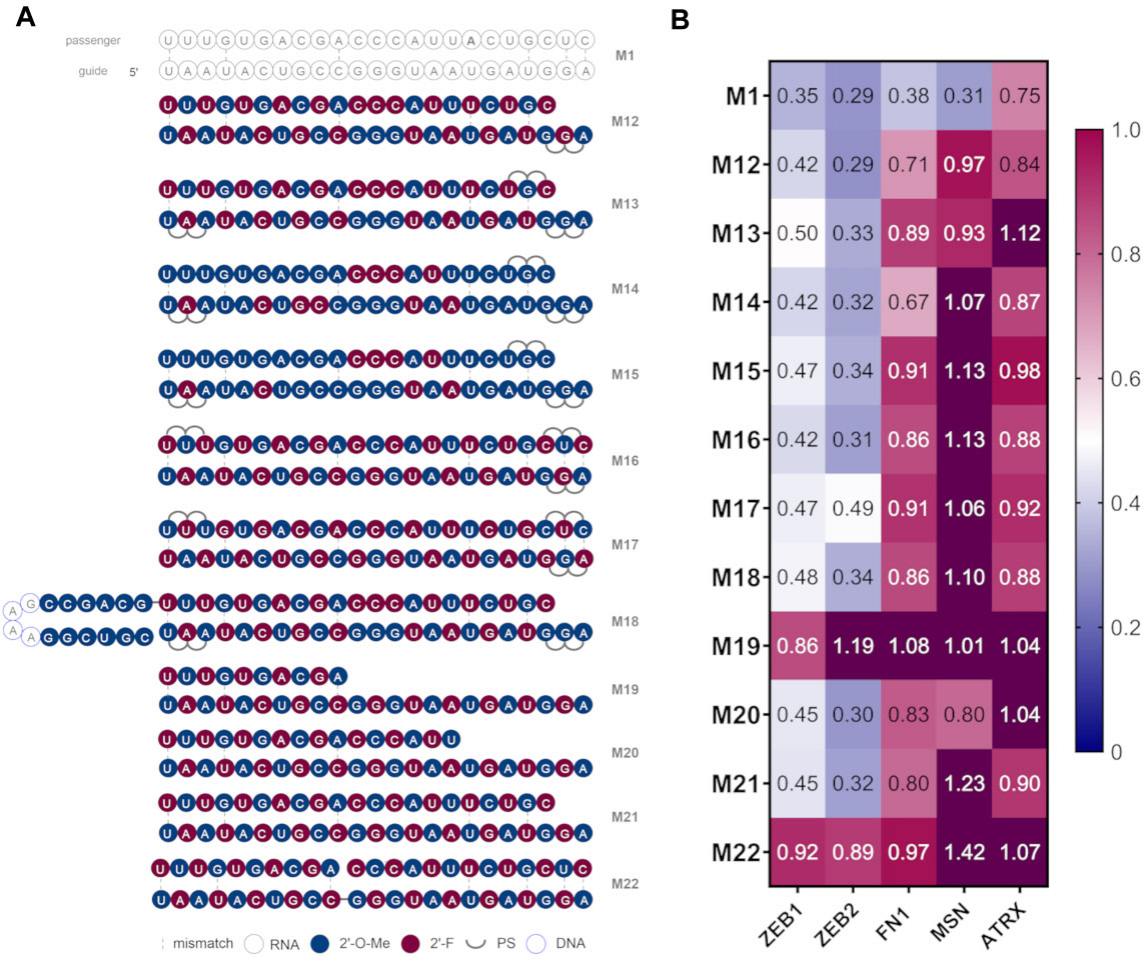
(**A**) Structures of the second generation of miR-200c mimics. (**B**) Knockdown data of second generation miR-200c mimics. A549 cells were transfected with 10 nM final concentration of mimics for 24 h then analysed by RT-qPCR (*n* = 3). Target mRNA expression was normalised to HPRT1 and baseline levels (control) were set to 1. The heat map indicates the average expression of each mRNA targeted by the different mimics (red = 1; blue = 0).

In the second design round we investigated commonly used siRNA modification patterns and their translatability into miRNA mimic activity. Pioneering companies in the therapeutic siRNA field, have introduced and evolved several chemical modification patterns for use in siRNA drugs and tool compounds (14,47,48). Several successful published chemistries were adapted to miR-200c in duplexes **M12** to **M17** (Figure 3A). That included Standard Template Chemistry (STC, **M12**), Enhanced Stabilization Chemistry (ESC, **M13**), Advanced ESC (**M14** and **M15**) and variations of alternating patterns (**M16** and **M17**). All designs tested presented improved activity for most genes (excluding MSN) compared to the mimics from the first series (Figure 3B, Supplementary Figure S8, Table S4). While robust knockdown on ZEB1 and ZEB2 was maintained, we also observed improved KD for FN1. In particular, the design **M14** proved to be nearly as effective as the unmodified miR-200c for ZEB1 and ZEB2 (**M1**).

The next design set explored the role of the duplex architecture (Figure 3A). A longer passenger strand was employed in **M18**. This structure was reported to assemble into a hairpin upon annealing, which resembles a pre-miRNA and can thus be loaded earlier into RISC. This approach was demonstrated to be beneficial to siRNA activity (1,49). The guide strand of **M18** was the same as in **M13**. Interestingly, the hairpin structure **M18**, has slightly improved activity as compared to **M13** for ZEB1, FN1 and ATRX (Figure 3B). Finally, we also explored asymmetric siRNA architectures (50,51). A shortened passenger strand was described to be associated with less passenger strand mediated off-target effects and diminished immune responses (52,53). We explored duplexes with alternating modification patterns (as in **M2**), but where the passenger strand was 10, 16 and 21 nucleotides long (**M19**, **M20**, **M21**, respectively). Moreover, we introduced a ‘nick’ in the passenger strand, so rather than a single strand, two shorter fragments complementary to the guide were employed (**M22**). Asymmetric miRNAs (**M20** and **M21**) had slightly better potency for ZEB1 and for **M21** for remaining genes than their counterpart with the fully complementary passenger (**M2**). However, a critical length of passenger strand had to be maintained as design **M19** lost potency compared to **M2**. The nicked design, **M22**, has proved to not to be effective in KD of the target mRNAs. This might be due to potentially incorrect duplex assembly and/or stability issues when complementary fragments which may have been too short were introduced.

#### Series 3

**Figure 4.**
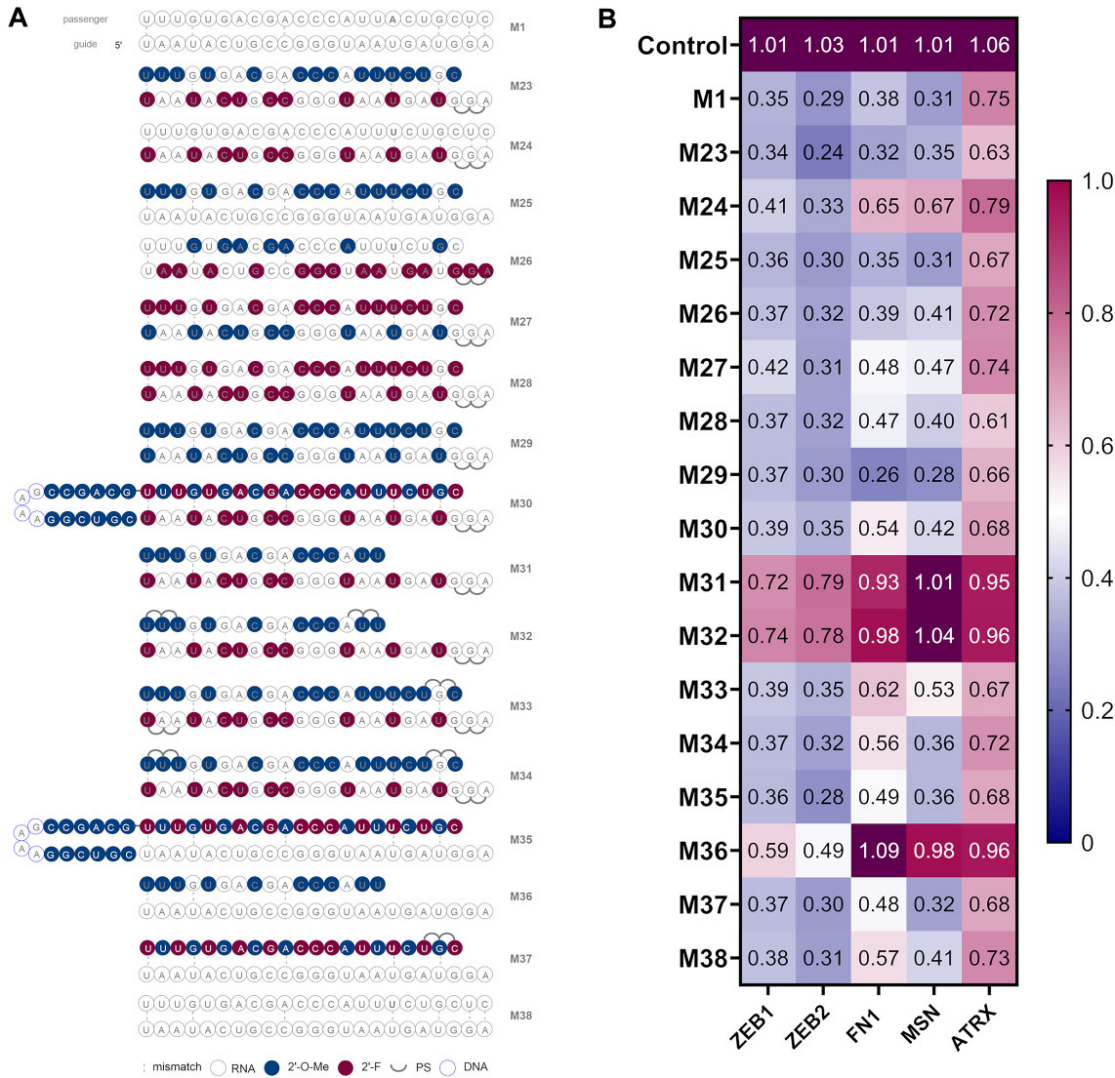
(**A**) Structures of third generation miR-200c mimics. (**B**) Knockdown data of third generation miR-200c mimics. A549 cells were transfected with 10 nM final concentration of mimics for 24 h then analysed by RT-qPCR (*n* = 3). Target mRNA expression was normalised to HPRT1 and baseline levels (control) were set to 1. The heat map indicates the average expression of each mRNA targeted by the different mimics (red = 1; blue = 0).

In the final round of design, we explored published miRNA patterns. Namely, we examined the modification pattern used for miR-29b mimics, which was employed in phase 2 clinical trials for fibrosis regulation as described by Montgomery *et al.* (54). In the final design, all pyrimidine containing nucleosides in the guide strand were modified in the 2′ position of the ribose with fluoro residues and in the passenger strand with methoxy moieties. We adapted this pattern to the miR-200c mimic as **M23** (Figure 4A). This design demonstrated superior activity, similar or better for all five targets as compared to unmodified **M1** (Figure 4B, Supplementary Figure S9, Table S5). We therefore explored more variations on this design and removed modifications from the passenger (**M24**) and guide strand (**M25**). This led to a maintained activity for **M25**, but decreased KD values in the case of **M24**, highlighting the role of modifications within the passenger strand. In design **M26**, purine nucleosides were functionalised with either 2′-F or 2′-OMe, instead of pyrimidine ones as in **M23**. This led to a slight loss of activity. In design **M27**, we inverted 2′-OMe and 2′-F modifications, so that they modified pyrimidines in guide and passenger strands respectively. This led to diminished potency on all targets as compared to **M23**. Next, in **M28** and **M29** purine nucleosides in both strands were modified with 2′-F and 2′-OMe on ribose, respectively. The fully 2′-OMe modified design **M29** was very similar in activity to **M23**.

For the next group of mimics, we carried over the guide strand from **M23** and used the best passenger strands from previous designs rounds (Figure 4A). **M30** contained the hairpin design from **M18**. **M31** and **M32** had shortened passenger strands (16-mer) from asymmetric designs possessing 2′-OMe in the ribose of pyrimidine nucleosides, with extra PS linkages in **M32**. Finally, **M33** and **M34** were analogous designs to **M31** and **M32**, but with a 21-nucleotide long passenger strand. Use of a hairpin passenger lowered KD efficiency (**M30**), except on ATRX where comparable knockdown to **M23** was measured (Figure 4B). When turning to asymmetric strands, there was almost complete loss of activity for mimics **M31** and **M32**. Variations from PS backbone on **M33** and **M34** both led to decreased knockdown, especially on FN1 and MSN. However, designs **M33** and **M34** resulted in comparable KD for ATRX to **M23**.

Finally, mimics containing the native guide strand, without modifications were generated (Figure 4A). Those consisted of a hairpin (**M35**), a 16-mer asymmetric passenger (**M36**), a fully modified passenger (**M37**) and a native passenger with a mismatch at position 7 (**M38**). The presence of a hairpin passenger in **M35** only affected the FN1 KD while the other targets showed similar levels to M23 (Figure 4B). The shorter passenger strand in **M36** led to a significant loss of activity in all cases. However, coupling a native guide with a fully modified passenger strand (**M37**) maintained a comparable profile to **M1**. Finally, the control with a native passenger with a U at SS7 exhibited a slightly lower overall activity (**M38**).

Among all synthesised duplexes, eight were selected for a further profiling, including the unmodified duplex (**M1**). Those selected displayed a variety of activities and designs. A comparison of knockdown data of shortlisted mimics is represented in Supplementary Figure S10.

### Luciferase reporter assay

**Figure 5.**
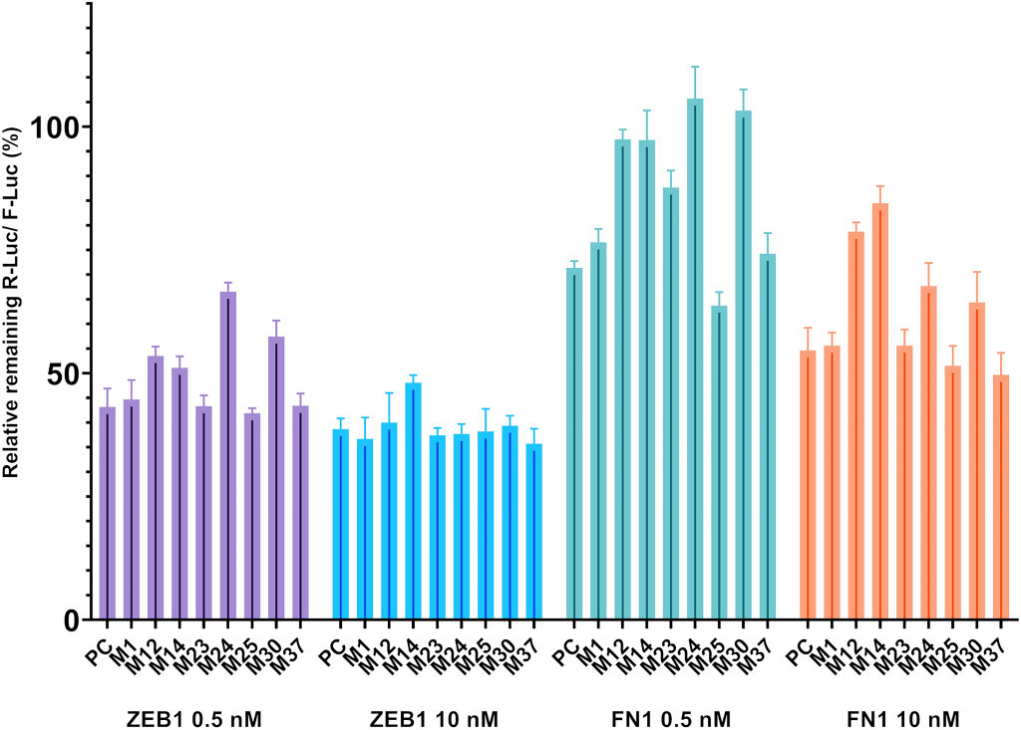
Luciferase reporter assay data. Hepa 1–6 cells were transfected with either 0.5 or 10 nM final concentration of mimics, for 24 h and analysed by luminescence (*n* = 4). R-Luc signal normalised with respective F-Luc signal with 100% as baseline. A commercial mimic (miRIDIAN®) was employed as positive control (PC).

In order to confirm that mimics act via RNAi mediated knockdown of the selected targets, we evaluated the binding of the eight shortlisted mimics to 3′-UTRs of two representative targets: ZEB1 and FN1 (37,39). This was accomplished by a dual luciferase reporter assay. Upon hybridization-dependent mRNA degradation of the mimic to such construct a decrease in luminescence can be observed (assay and construct details: Materials and Methods, SI Section 5). Results are summarized in Figure 5 (statistical analysis in Figure S11).

ZEB1 was efficiently targeted by all mimics at 10 nM, in agreement with the RT-qPCR results. While the other mimics maintained their activity even at the lower dose (0.5 nM), **M12, M14**, **M24** and **M30** appeared to be the least active mimics (Supplementary Figure S11). In general, activity of the mimics was lower when targeting FN1. This may be due to fewer miR-200c binding sites within FN1 3′-UTR as compared to ZEB1. The most active mimics (**M23**, **M25** and **M37**) were as potent as unmodified **M1** and one commercial mimic (**PC**) in the conditions assayed. In general, fully modified mimics such as **M12** or **M14** had a lower activity for both targets. **M24** and **M30** showed concentration dependent drop in potency with ZEB1 and, to an extent, FN1.

### Dose–response study

**Figure 6.**
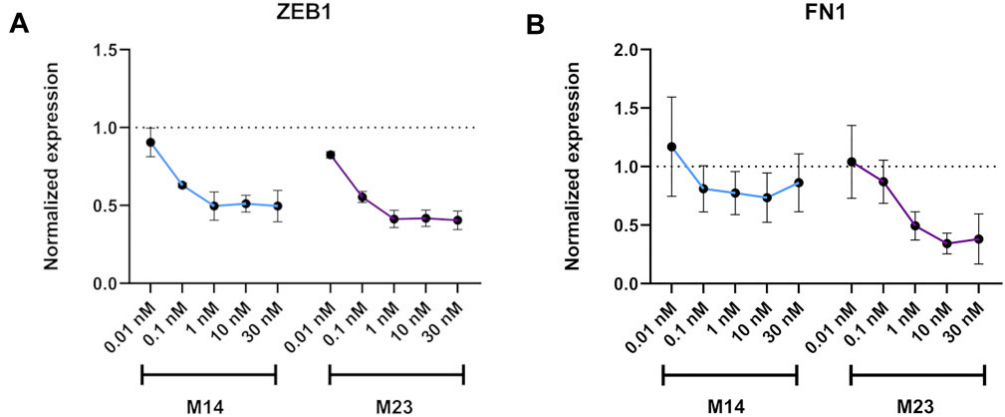
Dose-response data. A549 cells were transfected with 0.01–30 nM final concentration of mimics, for 24 h, and analysed by RT-qPCR (*n* = 3). Target mRNA expression was normalised to HPRT1 and baseline levels (untreated control) were set to 1. The plot indicates the average KD by each mimic per target with standard deviation as the error bar. (**A**) miR-200c mimics activity related to ZEB1 expression (**B**) miR-200c mimics activity related to FN1 expression.

For two selected designs among the fully modified group (**M14**) and partially modified family (**M23**), a dose response curve was performed with concentration of the mimics ranging from 0.01 to 30 nM and KD of ZEB1 and FN1 by qPCR were used as readouts. For both targets, the maximum KD was reached at 1 nM and a clear concentration dependency could be observed (Figure 6).

### Mimic stability

**Figure 7.**
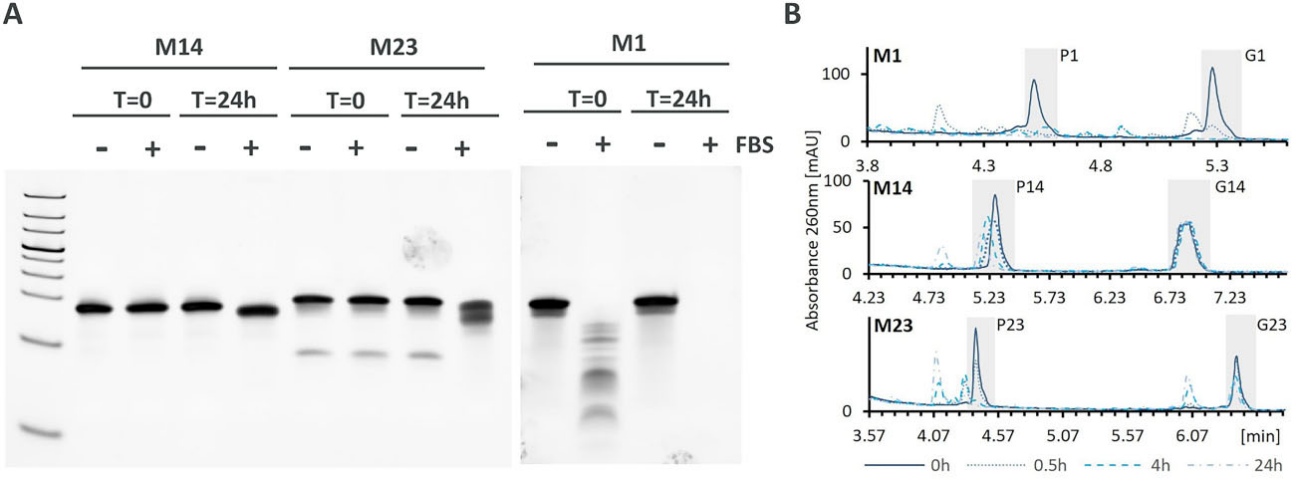
Stability of selected miRNA designs. (**A**) Stability of **M1, M14** and **M23** in FBS as assessed by non-denaturing gel electrophoresis. Line corresponds to time points 0 and 24 h incubation in FBS (+) and PBS (-). (**B**) stability of **M1, M14** and **M23** in rat serum as assessed by denaturing anion-exchange chromatography.

Chemical modifications can clearly influence the activity of miRNA mimics, however when used *in vivo* stability plays a major role in the final efficacy of the mimic (55–57). To approximate the *in vivo* behavior of the mimics, we have determined their stability in fetal bovine serum (FBS) (Figures 7, S12). Eight selected mimics were incubated in 80% FBS and samples were taken upon initial addition (T = 0) and then after 24 h. Additionally, a control containing the mimic in PBS without serum addition was run in a similar manner.

Unmodified mimic **M1** was substantially and completely degraded in the presence of FBS at time points 0 and 24 h, respectively. Partially modified mimics **M24**, **M25** and **M37** have already shown signs of degradation upon first contact with serum and were completely degraded after 24 h (Supplementary Figure S12). This is likely due to one of the strands in those mimics being completely unmodified. Mimics **M23** and **M30**, where both strands contained partial or full chemical modifications, appeared stable at time point zero, but showed degradation at 24 h. Finally, fully modified mimics **M12** and **M14** were stable under the tested conditions, with **M12** showing some signs of degradation at 24 h. This may be due to reduced PS content as compared to **M14**. All mimic samples incubated in PBS were stable over 24 h time period (Supplementary Figure S12).

Additionally, we selected 2 mimics to investigate strand degradation upon incubation with rat serum. Stability of the mimics was assessed in 80% rat serum over the course of 0.5, 4 and 24 h followed by anion-exchange chromatography analysis under denaturing conditions. The analysis revealed strand-specific stability. While both strands of unmodified miRNA **M1** were almost completely degraded after 0.5 h, **M23** and **M14** showed increased stability (Figure 7B). The antisense strand of **M14** (**G14**) was stable over 24 h and the corresponding sense strand (**P14**) appeared unstable due to some smaller degradation products which are not completely chromatographically resolved under the chosen conditions. This is consistent with extra PS modifications in the 3′end of **G14** as compared to **P14**. In **M23**, the antisense strand (**G23**) was more stable than the sense strand (**P23**), which was mostly degraded within 4 h. Overall miRNA stability could be ranked as **M14**> **M23**> **M1**, which follows the increasing number of chemical modifications in miRNA mimics.

### RNA-sequencing

**Figure 8.**
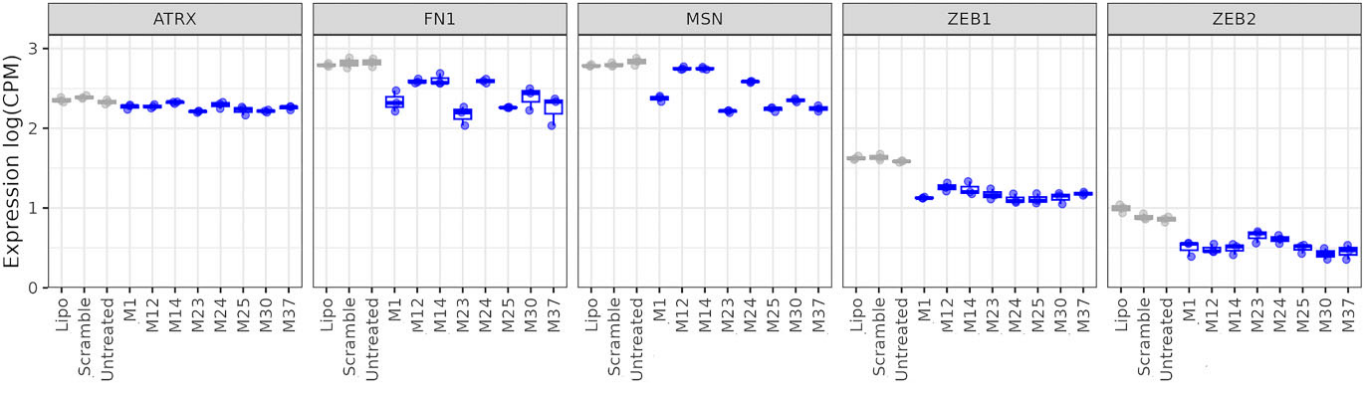
RNA-sequencing data on ATRX, FN1, MSN, ZEB1 and ZEB2. Expression of selected target genes based on the NGS analyses is given in log counts per million (CPM). Controls are shown in grey and mimics in blue.

Thus far, our screening cascade consisted of five representative miR-200c mRNA targets. This allowed for the rapid and efficient evaluation of the mimics. However, to fully understand the influence of chemical modifications on the activity and selectivity of the different mimics, we conducted a next generation sequencing (NGS) experiment on RNA isolated from A549 cells treated with the eight shortlisted mimics. Compounds were assayed at a final concentration of 10 nM. Controls consisted of media only, lipofectamine only (mock) and a negative transfection control (scramble). The results were compared to untreated (media only) control as all controls tended to cluster in the PCA (Supplementary Figure S13). Initially, we examined the effect of the mimics on previously selected targets (Figure 8). We observed downregulation of ZEB1 and ZEB2 with most of the mimics, confirming the RT-qPCR results, where these were the two most responsive targets. ATRX, similar to RT-qPCR data, had the lowest response rate. MSN and FN1 were regulated to a varying extent, also following the previous observations.

**Figure 9.**
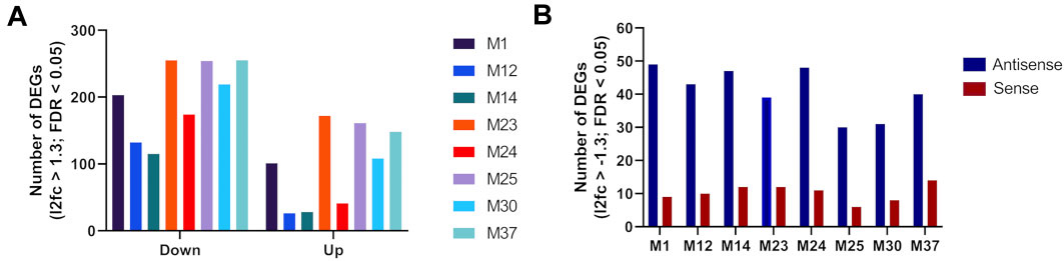
(**A**) Differentially expressed genes (DEGs) by RNA-sequencing with the number of downregulated genes on the left of the graph and upregulated genes to the right. (**B**) Number of downregulated DEGs with regions fully complementary to the seed sequence of antisense and sense strand of the miR-200c mimics versus the untreated control.

Subsequently, we looked at the number of differentially expressed genes (DEGs) upon treatment with the shortlisted mimics. Again, we compared all mimics *versus* the untreated control and *versus* the unmodified miR-200c (**M1**). We observed that fully modified designs (**M12** and **M14**) regulated a smaller number of genes (Figure 9A). This might be correlated to their general lower activity and/or higher selectivity. The partially modified designs were either similar to **M1** or had more regulated targets. Additionally, we have analysed identified downregulated DEGs for matches in the 3′ UTR for the seed sequence from the antisense and sense strand of the mimics (Figure 9B). This can be used as an approximation of the DEGs related to undesired activity, when the sense strand is loaded into Ago2 instead of the antisense strand. In all cases there were more matches related to the desired antisense strand activity than the sense strand, with ratios similar to that of the unmodified mimic **M1** (Table S6).

### Translatability of miRNA mimic designs

**Figure 10.**
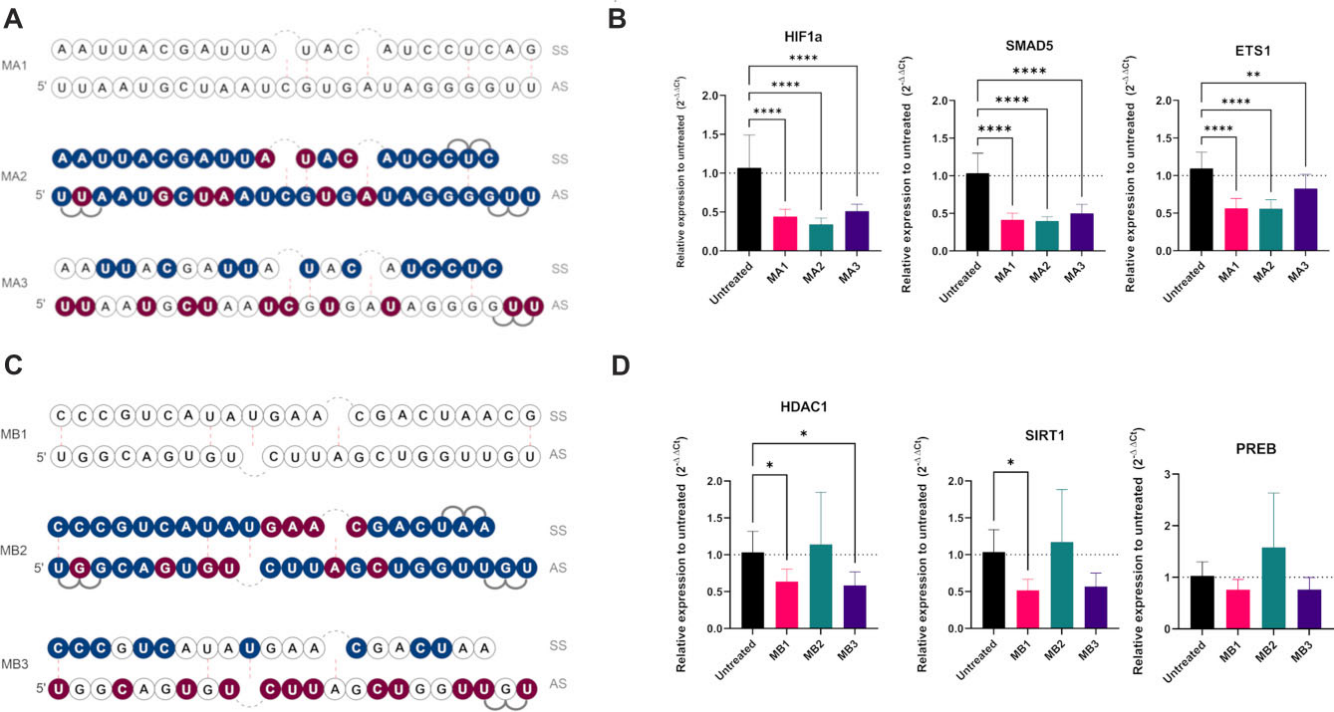
Translatability of M14 and M23 designs. (**A**) Designs for miR-155: **MA1** – unmodified mimic, **MA2** – design based on **M14**, **MA3** – design based on **M23**. (**B**) KD of miR-155 target genes ETS1, HIF1a and SMAD5 at 30 nM concentration of mimics (*n* = 3). (**C**) Designs for miR-34a: **MB1** – unmodified mimic, **MB2** – design based on **M14**, **MB3** – design based on **M23** (**D**) KD of miR-34a target genes HDAC1, PREB and SIRT1 at 10 nM concentration of mimics (*n* = 3). Statistical significance between the mimics and the untreated control are shown. * *P*< 0.05; ** *P*< 0.01; *** *P*< 0.001; **** *P*< 0.0001.

To determine if the selected mimic designs (**M14** and **M23**) were effective with other miRNA sequences, two additional miRNAs, miR-34a and miR-155, were chosen based on their role in the lung as well as the breadth of data available on their function (58–61). *In silico* prediction of the targets for miR-34a and miR-155 was done as described for miR-200c and gene lists were compared to those present on targeted miR-34a and miR-155 RT^2^ Profiler Arrays (Qiagen) to select candidate target genes for testing. For miR-34a, HDAC1, PREB and SIRT1 and for miR-155, ETS1, HIF1a and SMAD5 were chosen as target genes. Concentration curves were performed using commercially available miRNA mimics in A549 cells and 10 nM and 30 nM were selected for future testing with miR-34a and miR-155 mimics, respectively (Supplementary Figure S14 and S15). For the tested target mRNAs, the maximum KD efficiency was approximately 50%, similar to what we observed for miR-200c (Supplementary Figure S4). Subsequently, we adapted designs from **M14** and **M23** to miR-155 and miR-34a (Figure 10A, C) to obtain **MA2** and **MB2** (design **M14** adapted to miR-155 and miR-34a, respectively) and **MA3** and **MB3** (design **M23** adapted to miR-155 and miR-34a, respectively).**MA1** and **MB1** corresponded to unmodified miR-155 and miR-34a, respectively.

**Figure 11.**
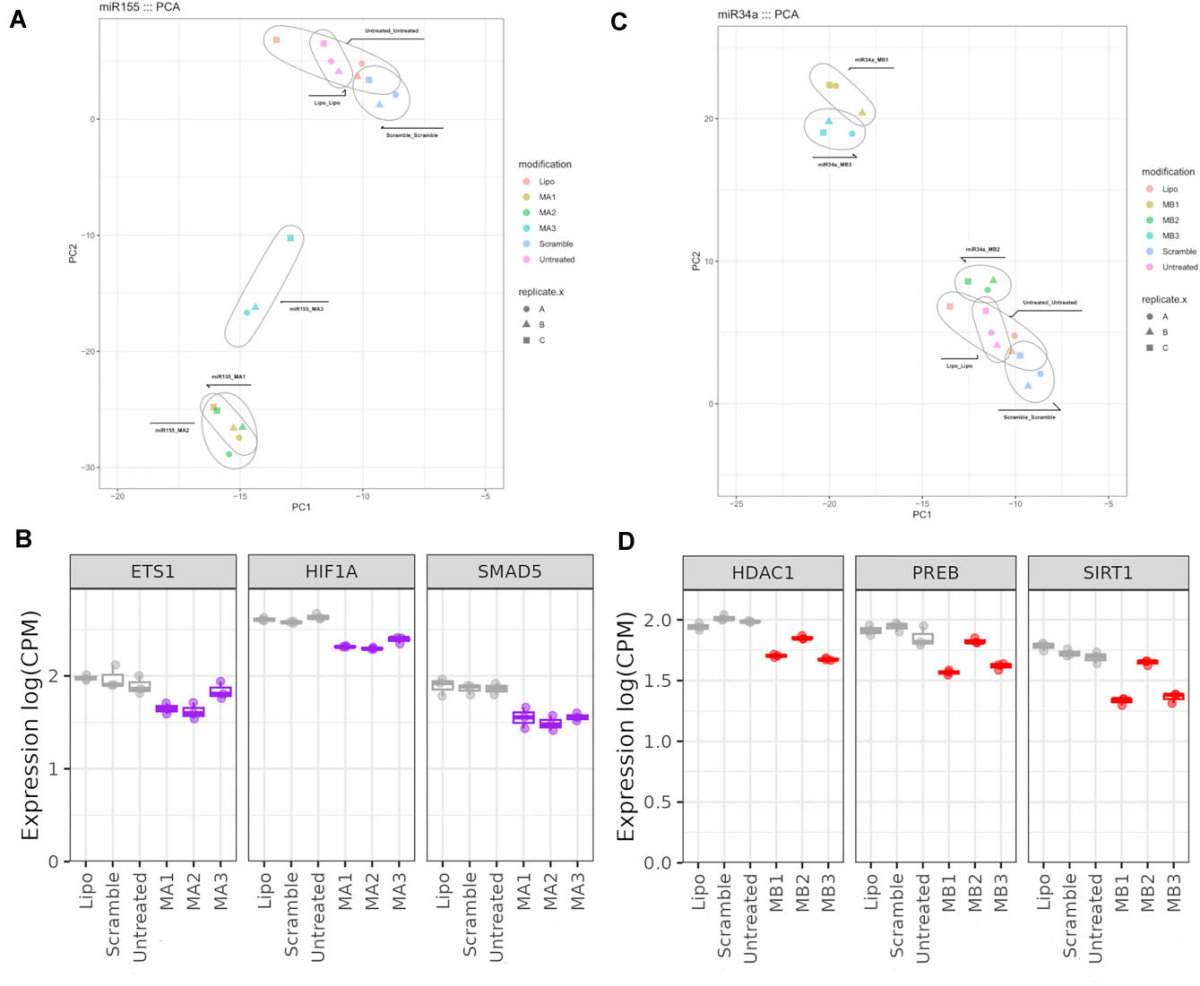
Profiling of miR-155 and miR-34a mimics by NGS (**A**) PCA analysis for **MA1**, **MA2** and **MA3. (B)** Expression of selected miR-155 target genes in the NGS analysis in counts per million (CPM). Controls are shown in grey and mimics in purple. (**C**) PCA analysis for MB1, MB2 and MB3. (**D**) Expression of selected miR-34a target genes in the NGS analysis in counts per million (CPM). Controls are shown in grey and mimics in red. *A, B, C* present in figures (A) and (C) indicates different biological replicates performed.

New mimics were then tested in qPCR assay for KD of selected target genes. **MA2** was able to downregulate genes in a similar manner to **MA1**, however, the **MA3** design regulated selected genes to a lesser extent (Figure 10B and Table S7). The opposite was observed for the miR-34a where **MB3** was able to downregulate miR-34a target mRNAs to a similar degree than unmodified mimic **MB1**, but **MB2** was not active (Figure 10D and Table S8). In the next step, the designs **MA1**-**MA3** and **MB1**-**MB3** were examined by NGS. Like the qPCR results, we found that mimics **MA1** and **MA2**, similarly **MB1** and **MB3** clustered by PCA, meaning that they were more similar to each other than to **MA3** or **MB2**, respectively (Figure 11A, C). We then examined the expression counts of the specific target mRNAs for each mimic- ETS1, HIF1a and SMAD5 for miR-155 and HDAC1, PREB1 and SIRT1 for miR-34a- from the NGS analysis. We found that the patterns were very similar to that observed by qPCR, with increased counts for both **MA3** and **MB2** (Figure 11B, D).

## Discussion

miRNAs have an inherent ability to impact many targets, regulating entire biological pathways. In the many diseases where miRNAs are downregulated, a replacement therapy with miRNA mimics represents an interesting therapeutic approach allowing for complex changes in cell homeostasis. However, due to the complexity of the interaction networks affected by miRNA mimics, extra care is required when designing and testing potential therapeutic or tool molecules. To be proven successful, those compounds need to present a very close activity pattern to their endogenous miRNA counterpart, while also having a favourable half-life and overall pharmacokinetic profile. To date, despite some clinical candidates, information on designing miRNA mimics remains scarce (18). Only a limited number of mimics were tested in a handful of reports (54,62–66). Often tool compounds for biological target validation purposes are ordered from commercial vendors, where chemical modifications of the mimics are hidden behind intellectual property firewalls. Thus, we aimed to provide the broad screening of common oligonucleotide chemical modifications and to assess their influence on the potency and selectivity of miRNA mimics.

In this work, we have selected an unmodified miR-200c duplex (**M1**) as the baseline for desired activity and specificity of the developed mimics. In an iterative design process, we evaluated different chemical modification patterns with RT-qPCR as a read-out for five direct target mRNAs in A549 cells.

Initial design rounds began with a simple alternating pattern of 2′-F and 2′-Ome ribose modifications in both the guide and passenger strand, as this is one of the earliest and most widely used patterns for siRNA design. miRNA mimics have a very similar structure to siRNA molecules and use the same enzymatic machinery to achieve translational repression of the target. This prompted us to explore different variations of siRNA modification patterns and check their translatability to miRNA mimic design. Early variations of the ‘alternating pattern’ yielded mimics displaying a good inhibition of two targets, however, activity for the other three remained to be optimised. The next series of designs employed more advanced chemistry used for siRNA design *in vivo* (14,47), improved activity profiles, with selectivity closer, but not identical to that of the unmodified duplex (**M1**). In particular, the designs **M12** and **M14**, based on the work from Milstein and co-workers (47), were the most potent among the fully modified mimics. Both siRNAs and miRNAs share many commonalities, however, it was also imperative to remember that an siRNA is optimised for target specificity, while we want to retain a more promiscuous profile for miRNAs, with many targets affected by one miRNA. This was confirmed by the RNA-seq data, where smaller knockdown fold changes correlated with fewer targeted mRNAs as in the case of **M14**.

In the fully modified mimic groups, we also investigated additional parameters, such as PS content and overall duplex architecture. The position and number of PS modifications indeed influenced the activity of the mimics, however not to a major extent. This modification has been known in literature to provide some nucleolytic stability (67) and modulate plasma protein binding and, therefore, might have influence in the *in vivo* mimic designs (14). The duplex architecture study has provided us with an interesting insight into the role of the passenger strand in an effective miRNA mimic. The miRNA duplex has to be stable enough to be efficiently loaded into the RISC, but also the passenger strand has to be efficiently unwound to provide an active form of the guide strand loaded into Ago. Our data demonstrated that a shorter passenger strand can provide an advantage, but only to a certain length. If the strand sequence is too short, mimics lose activity, possibly due to the lack of duplex stability. This was also observed where two separate passenger fragments were introduced instead of one uniform strand (**M22**). Significant shortening of the passenger strand was also only beneficial in the context of fully modified mimics, as partially modified designs (**M31**, **M32**, **M36**) were characterised by almost complete loss of activity. Shorter passenger strands are generally reported to possess fewer off-targets (52,53), therefore their use could be further explored within fully modified potent designs. The reverse approach using hairpin like passenger strands, which could enable earlier loading of the duplex into RISC, proved to be successful when they were combined with various efficient guides (**M18**, **M30**, **M35**).

Another distinctive feature of miRNAs is the presence of several mismatches within the endogenous duplex. This destabilizes the duplex and allows for easier removal (68) of the passenger strand from RISC, but also prevents the passenger strand from acting as an antagomir in a RISC independent manner (18,69). This was clearly illustrated by the fully complementary mimic (**M11**), which was completely inactive and may explain the poor performance of **M24** possessing a native passenger and modified guide. We then studied the influence of mismatch on a single position (7 in the passenger strand) within the mimic. Variation between either a deletion (**M9**), a match (**M2**) or a mismatch (**M10**) led to slightly different activity patterns across targets with somewhat better activity with a mismatch (Figure 2B).

The miR-29b mimic from Montgomery *et al.* is one of the best described miRNA mimics designed for clinical use (54). We tested the modification pattern employed for this mimic on our miR-200c model and **M23** was proven to be the most effective mimic among the tested designs. In this pattern, pyrimidine nucleosides of the guide strand were modified with 2′-F ribose, while the passenger strand had respective 2′-OMe modifications. This assembly is reported to allow for loading of a duplex in the correct orientation into RISC, with bulkier 2′-OMe modifications being unfavourable for the passenger strand (54). Subsequently, we investigated other partially modified miRNA mimics. Among those, a hairpin design (**M30**) possessed very similar activity to the unmodified duplex **M1**. Finally, annealing an unmodified guide strand with either a mixed passenger (**M25**) or fully modified design (**M37**) was a good strategy for an *in vitro* tool compound (Figure 3B and 4B).

Overall, we have shown that the desirable activity can be achieved with both fully and partially modified mimics, with their best representative examples being **M14** and **M23**. Transcriptome sequencing further confirmed favourable profiles among the eight shortlisted mimics, with duplexes **M23**, **M25**, **M30** and **M37** being very close to **M1** in terms of the number of similarly regulated mRNAs (Figure 8).

The next question was if the identified modification patterns would be of use to another miRNA. We have demonstrated that pattern from miR-29b could be successfully translated to the miR-200c mimic (**M23**). We then selected two additional miRNAs: miR-155 and miR-34a and synthesized their mimics based on modification patterns from **M23** and **M14** (**MA3** and **MB3**, respectively; Figure 10A). The **M23** pattern translated very well for miR-34a and also had an acceptable profile for miR155, suggesting that it is a good choice of chemical modifications for an *in vitro* tool miRNA mimic. Template **M14** provided an active an active miR-155 mimic (**MA2**), but very little activity was observed on the selected target genes for miR-34a (**MB2**). This highlights the need of sequence specific optimization of chemical modifications in miRNA mimics.

Partially modified mimics, in general, had better profiles as *in vitro* tool compounds. However, it is worth noting that fully modified mimics may be a better starting point for the design of mimics suitable for *in viv*o use. This has been nicely showcased in a recent publication on a next generation miR-29b mimic (57) and miR-34a mimic (56). The initial design for miR-29b consisted of an only partially modified duplex, but it was not pursued beyond Phase 2 clinical trial due to low efficacy (16). The efficacy of the miR-29b mimic was significantly improved with the fully modified design leading to a more desirable PK profile (57). Similarly for miR-34a, a partially modified mimic was less efficient *in vivo* compared to a fully modified mimic, even though they presented similar target gene KD *in vitro*. Another layer of the mimic's efficacy is supported by half-life optimisation. We have measured degradation of the fully and partially modified miRNA mimics in serum as an approximation of the *in vivo* stability ranking (Figure 7 and Supplementary Figure S12). Unsurprisingly, the stability was correlated with the level of chemical modifications in the mimic, suggesting that fully modified designs, even when less active *in vitro* might have advantage when used *in vivo*. Translation of *in vitro* results to *in vivo* efficacy still represents a considerable challenge in the development of oligonucleotide therapeutics, therefore of course enhanced stability *in vitro* may not result in therapeutic effects *in vivo*.

The goal of this work was to develop a tool miRNA mimic for *in vitro* studies that possesses an activity pattern as close as possible to the native miRNA while also being chemically stabilised. We present the evaluation of several different chemical modification patterns and provide an initial SAR observation for successful miRNA mimic design. In our system, we used miR-200c as a case study and further took two selected designs, **M14** and **M23**, and applied them to miR-34a and miR-155. Encouragingly, some of the patterns utilised have already shown good translatability between different miRNAs. For instance, **M23** has been a pattern of choice for initial miR-29b clinical candidate and in this work was proven successful in an *in vitro* tool miR-200c, miR-34a and to large extent miR155 mimic. Similarly, the fully modified design **M14** demonstrated a good profile as a miR-200c and miR-155 mimic in our study and has been employed in another clinical miR-29b design, as well as in many siRNA tool molecules.

## Supplementary Material

gkae141_Supplemental_File

## Data Availability

The RNA-seq FASTQ files reported in this article have been deposited in EMBL-EBI ArrayExpress (E-MTAB-13801).
