# Supplementary material for: Chemical modification patterns for microRNA therapeutic mimics: a structure-activity relationship (SAR) case-study on miR-200c
Source: Nucleic Acids Res. 2024 Feb 29;52(6):2792–807. doi: 10.1093/nar/gkae141 (PMC11014349; doi:10.1093/nar/gkae141)
Supplement: gkae141_Supplemental_File [file gkae141_supplemental_file.pdf]

**Chemical modification patterns for microRNA therapeutic mimics: a structure-activity relationship (SAR) case-study on miR-200c.**

**SUPPLEMENTARY DATA**

**Contents**


1. *In silico* target gene selection ........ 2
2. Ct values of miR-200c and mRNA targets in A549 cells ........ 3
3. Optimisation of the *in vitro* assay ........ 3
4. Duplexes sequences ........ 5
5. SAR study results ........ 7
6. Luciferase dual reporter assay ........ 15
7. Stability ........ 19
8. RNA-sequencing ........ 20
9. Translatability of mimic designs ........ 22
10. LCMS data ........ 24

References ........ 79

## 1. *In silico* target gene selection

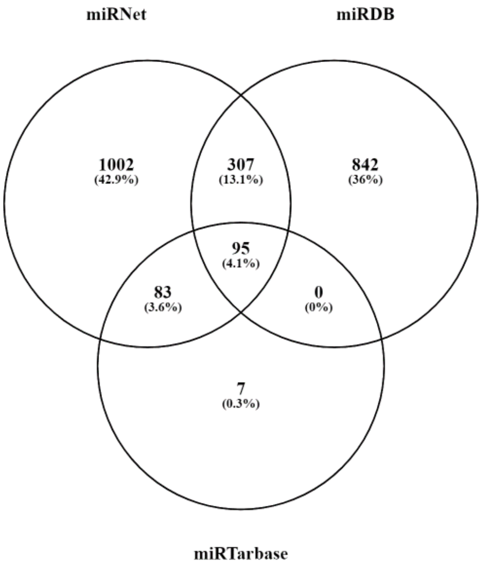


**Figure S1.** Intersection between database for hsa-miR-200c-3p target predictions (1-3).

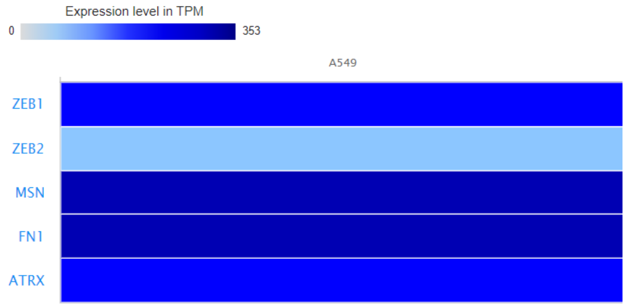


**Figure S2.** Expression levels of selected target genes in A549 cells. Data from Expression Atlas (4).

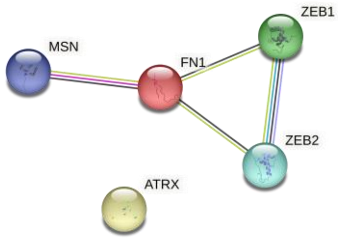


**Figure S3.** PPI interactions modeled by String (5).

## 2. Average Ct values of miR-200c and mRNA targets in A549 cells

| Target | Average Ct value |
|---|---|
| **hsa-miR-200c-3p** | 29.08 |
| **ZEB1** | 24.84 |
| **ZEB2** | 27.63 |
| **FN1** | 23.02 |
| **MSN** | 20.77 |
| **ATRX** | 24.38 |

**Table S1.** Average Ct values of miR-200c and mRNA targets in A549 cells. For assessing levels of miR-200c in the cells, total RNA was reverse transcribed into cDNA using the TaqMan MicroRNA Reverse Transcription kit (ThermoFisher Scientific) according to the manufacturer's instructions. The microRNA primers used were hsa-miR-200c (Assay ID: 002300) and RNU6 (Assay ID: Hs03910430; reference) were used to assess endogenous levels of miR-200c. Methodology and probes for the mRNA targets can be found in Materials and Methods under "*RNA extraction and TaqMan Assay.*" Ct values are the average of untreated cells in atleast 3 experiments in technical replicates.

## 3. Optimisation of the *in vitro* assay

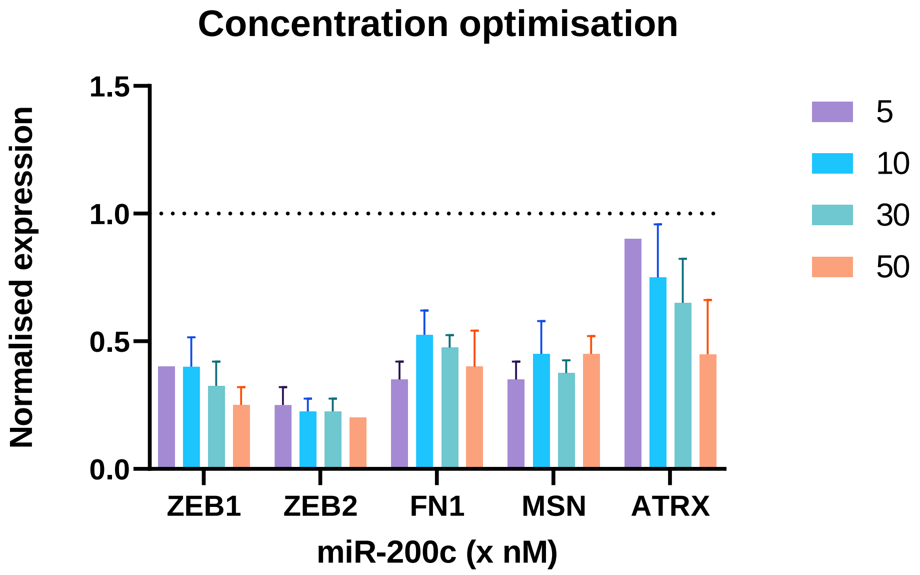


**Figure S4.** Knockdown (KD) data of concentration screening. A549 cells were transfected with 5, 10, 30 or 50 nM final concentration of commercial miRIDIAN® mimic for 24 h, then analysed by RT-qPCR (n=3). Target mRNA expression was normalised to HPRT1 and baseline levels (control) were set to 1 (dotted line). Data shown as average with standard deviation.

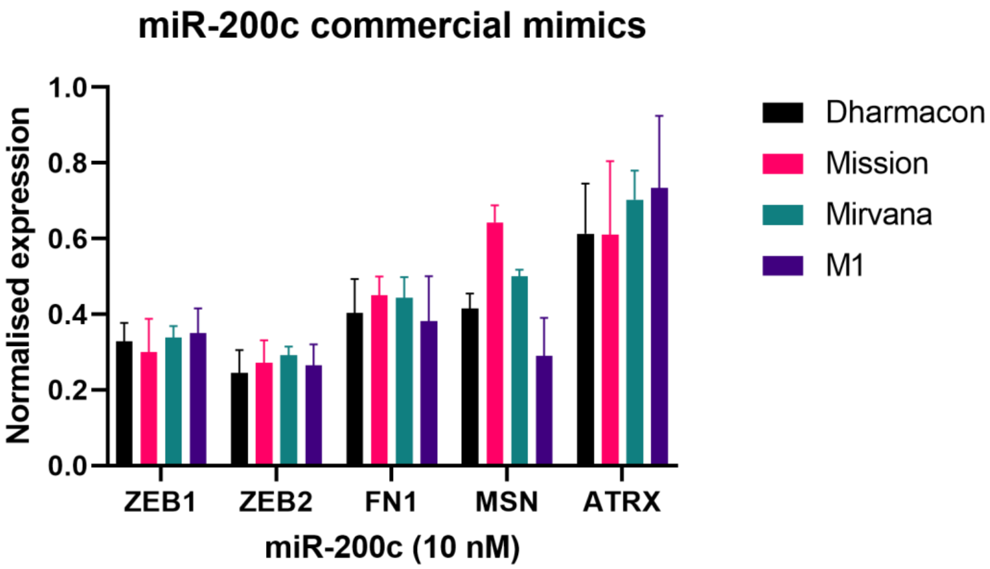


**Figure S5.** Knockdown (KD) data of commercial mimics. A549 cells were transfected with 10 nM final concentration of mimics for 24 h, then analysed by RT-qPCR (n=2). Target mRNA expression was normalised to HPRT1 and baseline levels (control) were set to 1. hsa-miR-200c-3p mimics from: miRIDIAN®, MISSION® and *mir*Vana® and the unmodified **M1** used throughout the study. Data shown as average with standard deviation.

## 4. Duplexes sequences

### Oligonucleotide modification legend:

X - DNA, rX - RNA, mX - 2'-methoxy, fX – 2'-fluoro, p – phosphate

* phosphorothioate linkage, - phosphate linkage

**Table S2.** Duplexes sequences

| Duplex | Guide | Guide sequence (5'-3') | Passenger | Passenger sequence (5'-3') |
|---|---|---|---|---|
| **M1** | **G1** | rU-rA-rA-rU-rA-rC-rU-rG-rC-rC-rG-rG-rG-rU-rA-rA-rU-rG-rA-rU-rG-rG-rA | **P1** | rC-rU-rC-rG-rU-rC-rA-rU-rU-rA-rC-rC-rA-rG-rC-rA-rG-rU-rG-rU-rU-rU |
| **M2** | **G2** | mU-fA-mA-fU-mA-fC-mU-fG-mC-fC-mG-fG-mG-fU-mA-fA-mU-fG-mA-fU-mG-fG-mA | **P2** | fC-mU-fC-mG-fU-mC-fA-mU-fU-mA-fC-mC-fC-mA-fG-mC-fA-mG-fU-mG-fU-mU-fU |
| **M3** | **G3** | fU-mA-fA-mU-fA-mC-fU-mG-fC-mC-fG-mG-fG-mU-fA-mA-fU-mG-fA-mU-fG-mG-fA | **P3** | mC-fU-mC-fG-mU-fC-mA-fU-mU-fA-mC-fC-mC-fA-mG-fC-mA-fG-mU-fG-mU-fU-mU |
| **M4** | **G2** | mU-fA-mA-fU-mA-fC-mU-fG-mC-fC-mG-fG-mG-fU-mA-fA-mU-fG-mA-fU-mG-fG-mA | **P3** | mC-fU-mC-fG-mU-fC-mA-fU-mU-fA-mC-fC-mC-fA-mG-fC-mA-fG-mU-fG-mU-fU-mU |
| **M5** | **G4** | P-mU-fA-mA-fU-mA-fC-mU-fG-mC-fC-mG-fG-mG-fU-mA-fA-mU-fG-mA-fU-mG-fG-mA | **P2** | fC-mU-fC-mG-fU-mC-fA-mU-fU-mA-fC-mC-fC-mA-fG-mC-fA-mG-fU-mG-fU-mU-fU |
| **M6** | **G5** | mU*fA*mA-fU-mA-fC-mU-fG-mC-fC-mG-fG-mG-fU-mA-fA-mU-fG-mA-fU-mG*fG*mA | **P4** | fC*mU*fC-mG-fU-mC-fA-mU-fU-mA-fC-mC-fC-mA-fG-mC-fA-mG-fU-mG-fU-mU-fU |
| **M7** | **G6** | mU*fA*mA-fU-mA-fC-mU-fG-mC-fC-mG-fG-mG-fU-mA-fA-mU-fG-mA-fU-mG-fG-mA | **P5** | fC*mU*fC-mG-fU-mC-fA-mU-fU-mA-fC-mC-fC-mA-fG-mC-fA-mG-fU-mG-fU*mU*fU |
| **M8** | **G7** | mU-fA-mA-fU-mA-fC-mU-fG-mC-fC-mG-fG-mG-fU-mA-fA-mU-fG-mA-fU-mG-fG-mA*mU*mU | **P2** | fC-mU-fC-mG-fU-mC-fA-mU-fU-mA-fC-mC-fC-mA-fG-mC-fA-mG-fU-mG-fU-mU-fU |
| **M9** | **G2** | mU-fA-mA-fU-mA-fC-mU-fG-mC-fC-mG-fG-mG-fU-mA-fA-mU-fG-mA-fU-mG-fG-mA | **P6** | fC-mU-fC-mG-fU-mC-mU-fU-mA-fC-mC-fC-mA-fG-mC-fA-mG-fU-mG-fU-mU-fU |
| **M10** | **G2** | mU-fA-mA-fU-mA-fC-mU-fG-mC-fC-mG-fG-mG-fU-mA-fA-mU-fG-mA-fU-mG-fG-mA | **P7** | fC-mU-fC-mG-fU-mC-fU-mU-fU-mA-fC-mC-fC-mA-fG-mC-fA-mG-fU-mG-fU-mU-fU |
| **M11** | **G2** | mU-fA-mA-fU-mA-fC-mU-fG-mC-fC-mG-fG-mG-fU-mA-fA-mU-fG-mA-fU-mG-fG-mA | **P8** | fU-mC-fC-mA-fU-mC-fA-mU-fU-mA-fC-mC-fC-mG-fG-mC-fA-mG-fU-mA-fU-mU-fA |
| **M12** | **G8** | mU-fA-mA-fU-mA-fC-mU-fG-mC-fC-mG-mG-mG-fU-mA-fA-mU-fG-mA-fU-mG*fG*mA | **P9** | fC-mG-fU-mC-fU-mU-fU-mA-fC-fC-fC-mA-fG-mC-fA-mG-fU-mG-fU-mU-fU |
| **M13** | **G9** | mU*fA*mA-fU-mA-fC-mU-fG-mC-fC-mG-mG-mG-fU-mA-fA-mU-fG-mA-fU-mG*mG*mA | **P10** | fC*mG*fU-mC-fU-mU-fU-mA-fC-fC-fC-mA-fG-mC-fA-mG-fU-mG-fU-mU-fU |
| **M14** | **G10** | mU*fA*mA-mU-mA-fC-mU-fG-fC-mC-mG-mG-mG-fU-mA-fA-mU-mG-mA-mU-mG*mG*mA | **P11** | mC*mG*mU-mC-mU-mU-fU-mA-fC-fC-fC-mA-mG-mC-mA-mG-mU-mG-mU-mU-mU |
| **M15** | **G11** | mU*fA*mA-mU-mA-fC-mU-mG-mC-mC-mG-mG-mG-fU-mA-fA-mU-mG-mA-mU-mG*mG*mA | **P11** | mC*mG*mU-mC-mU-mU-fU-mA-fC-fC-fC-mA-mG-mC-mA-mG-mU-mG-mU-mU-mU |
| **M16** | **G12** | mU-fA-mA-fU-mA-fC-mU-fG-mC-fC-mG-fG-mG-fU-mA-fA-mU-fG-mA-fU-mG*fG*mA | **P12** | fC*mU*fC-mG-fU-mC-fU-mU-fU-mA-fC-mC-fC-mA-fG-mC-fA-mG-fU-mG-fU*mU*fU |
| **M17** | **G13** | fU-mA-fA-mU-fA-mC-fU-mG-fC-mC-fG-mG-fG-mU-fA-mA-fU-mG-fA-mU-fG*mG*fA | **P13** | mC*fU*mC-fG-mU-fC-mU-fU-mU-fA-mC-fC-mC-fA-mG-fC-mA-fG-mU-fG-mU*fU*mU |
| **M18** | **G9** | mU*fA*mA-fU-mA-fC-mU-fG-mC-fC-mG-mG-mG-fU-mA-fA-mU-fG-mA-fU-mG*mG*mA | **P14** | fC-mG-fU-mC-fU-mU-fU-mA-fC-fC-fC-mA-fG-mC-fA-mG-fU-mG-fU-mU-fU-mG-mC-mA-mG-mC-mC-dG-dA-dA-dA-mG-mG-mC-mU-mG-mC |
| **M19** | **G2** | mU-fA-mA-fU-mA-fC-mU-fG-mC-fC-mG-fG-mG-fU-mA-fA-mU-fG-mA-fU-mG-fG-mA | **P15** | mA-fG-mC-fA-mG-fU-mG-fU-mU-fU |
| **M20** | **G2** | mU-fA-mA-fU-mA-fC-mU-fG-mC-fC-mG-fG-mG-fU-mA-fA-mU-fG-mA-fU-mG-fG-mA | **P16** | mU-fU-mA-fC-mC-fC-mA-fG-mC-fA-mG-fU-mG-fU-mU-fU |
| **M21** | **G2** | mU-fA-mA-fU-mA-fC-mU-fG-mC-fC-mG-fG-mG-fU-mA-fA-mU-fG-mA-fU-mG-fG-mA | **P17** | fC-mG-fU-mC-fU-mU-fU-mA-fC-mC-fC-mA-fG-mC-fA-mG-fU-mG-fU-mU-fU |
| **M22** | **G2** | mU-fA-mA-fU-mA-fC-mU-fG-mC-fC-mG-fG-mG-fU-mA-fA-mU-fG-mA-fU-mG-fG-mA | **P15+18** | mA-fG-mC-fA-mG-fU-mG-fU-mU-fU fC-mC-fC-mG-fU-mC-fU-mU-fU-mA-fC-mC-fC |
| **M23** | **G14** | fU-rA-rA-fU-rA-fC-fU-rG-fC-fC-rG-rG-rG-fU-rA-rA-fU-rG-rA-fU-rG*rG*rA | **P19** | mC-rG-mU-mC-mU-mU-mU-rA-mC-mC-mC-rA-rG-mC-rA-rG-mU-rG-mU-mU-mU |
| **M24** | **G14** | fU-rA-rA-fU-rA-fC-fU-rG-fC-fC-rG-rG-rG-fU-rA-rA-fU-rG-rA-fU-rG*rG*rA | **P20** | rC-rU-rC-rG-rU-rC-rU-rU-rU-rA-rC-rC-rC-rA-rG-rC-rA-rG-rU-rG-rU-rU-rU |
| **M25** | **G1** | rU-rA-rA-rU-rA-rC-rU-rG-rC-rC-rG-rG-rG-rU-rA-rA-rU-rG-rA-rU-rG-rG-rA | **P19** | mC-rG-mU-mC-mU-mU-mU-rA-mC-mC-mC-rA-rG-mC-rA-rG-mU-rG-mU-mU-mU |
| **M26** | **G15** | rU-fA-fA-rU-fA-rC-rU-fG-rC-rC-fG-fG-fG-rU-fA-fA-rU-fG-fA-rU-fG*fG*fA | **P21** | rC-mG-rU-rC-rU-rU-rU-mA-rC-rC-rC-mA-mG-rC-mA-mG-rU-mG-rU-rU-rU |
| **M27** | **G16** | mU-rA-rA-mU-rA-mC-mU-rG-mC-mC-rG- | **P22** | fC-rG-fU-fC-fU-fU-fU-rA-fC-fC-fC-rA-rG-fC- |

| | | | | |
|---|---|---|---|---|
| | | rG-rG-mU-rA-rA-mU-rG-rA-mU-rG*rG*rA | | rA-rG-fU-rG-fU-fU-fU |
| **M28** | **G14** | fU-rA-rA-fU-rA-fC-fU-rG-fC-fC-rG-rG-rG-fU-rA-rA-fU-rG-rA-fU-rG*rG*rA | **P22** | fC-rG-fU-fC-fU-fU-fU-rA-fC-fC-fC-rA-rG-fC-rA-rG-fU-rG-fU-fU-fU |
| **M29** | **G16** | mU-rA-rA-mU-rA-mC-mU-rG-mC-mC-rG-rG-rG-mU-rA-rA-mU-rG-rA-mU-rG*rG*rA | **P19** | mC-rG-mU-mC-mU-mU-mU-rA-mC-mC-mC-rA-rG-mC-rA-rG-mU-rG-mU-mU-mU |
| **M30** | **G14** | fU-rA-rA-fU-rA-fC-fU-rG-fC-fC-rG-rG-rG-fU-rA-rA-fU-rG-rA-fU-rG*rG*rA | **P14** | fC-mG-fU-mC-fU-mU-fU-mA-fC-fC-fC-mA-fG-mC-fA-mG-fU-mG-fU-mU-fU-mG-mC-mA-mG-mC-mC-dG-dA-dA-dA-mG-mG-mC-mU-mG-mC |
| **M31** | **G14** | fU-rA-rA-fU-rA-fC-fU-rG-fC-fC-rG-rG-rG-fU-rA-rA-fU-rG-rA-fU-rG*rG*rA | **P23** | mU-mU-rA-mC-mC-mC-rA-rG-mC-rA-rG-mU-rG-mU-mU-mU |
| **M32** | **G14** | fU-rA-rA-fU-rA-fC-fU-rG-fC-fC-rG-rG-rG-fU-rA-rA-fU-rG-rA-fU-rG*rG*rA | **P24** | mU*mU*rA-mC-mC-mC-rA-rG-mC-rA-rG-mU-rG-mU*mU*mU |
| **M33** | **G17** | fU*rA*rA-fU-rA-fC-fU-rG-fC-fC-rG-rG-rG-fU-rA-rA-fU-rG-rA-fU-rG*rG*rA | **P25** | mC*rG*mU-mC-mU-mU-mU-rA-mC-mC-mC-rA-rG-mC-rA-rG-mU-rG-mU-mU-mU |
| **M34** | **G14** | fU-rA-rA-fU-rA-fC-fU-rG-fC-fC-rG-rG-rG-fU-rA-rA-fU-rG-rA-fU-rG*rG*rA | **P26** | mC*rG*mU-mC-mU-mU-mU-rA-mC-mC-mC-rA-rG-mC-rA-rG-mU-rG-mU*mU*mU |
| **M35** | **G1** | rU-rA-rA-rU-rA-rC-rU-rG-rC-rC-rG-rG-rG-rU-rA-rA-rU-rG-rA-rU-rG-rG-rA | **P14** | fC-mG-fU-mC-fU-mU-fU-mA-fC-fC-fC-mA-fG-mC-fA-mG-fU-mG-fU-mU-fU-mG-mC-mA-mG-mC-mC-dG-dA-dA-dA-mG-mG-mC-mU-mG-mC |
| **M36** | **G1** | rU-rA-rA-rU-rA-rC-rU-rG-rC-rC-rG-rG-rG-rU-rA-rA-rU-rG-rA-rU-rG-rG-rA | **P23** | mU-mU-rA-mC-mC-mC-rA-rG-mC-rA-rG-mU-rG-mU-mU-mU |
| **M37** | **G1** | rU-rA-rA-rU-rA-rC-rU-rG-rC-rC-rG-rG-rG-rU-rA-rA-rU-rG-rA-rU-rG-rG-rA | **P9** | fC-mG-fU-mC-fU-mU-fU-mA-fC-fC-fC-mA-fG-mC-fA-mG-fU-mG-fU-mU-fU |
| **M38** | **G1** | rU-rA-rA-rU-rA-rC-rU-rG-rC-rC-rG-rG-rG-rU-rA-rA-rU-rG-rA-rU-rG-rG-rA | **P20** | rC-rU-rC-rG-rU-rC-rU-rU-rU-rA-rC-rC-rC-rA-rG-rC-rA-rG-rU-rG-rU-rU-rU |
| **MA1** | **GA1** | rU-rU-rA-rA-rU-rG-rC-rU-rA-rA-rU-rC-rG-rU-rG-rA-rU-rA-rG-rG-rG-rG-rU-rU | **PA1** | rG-rA-rC-rU-rC-rC-rU-rA-rC-rA-rU-rA-rU-rU-rA-rG-rC-rA-rU-rU-rA-rA |
| **MA2** | **GA2** | fU-fU-rA-rA-fU-rG-fC-fU-rA-rA-fU-fC-rG-fU-rG-rA-fU-rA-rG-rG-rG-rG*fU*fU | **PA2** | mC-mU-mC-mC-mU-rA-mC-rA-mU-rA-mU-mU-rA-rG-mC-rA-mU-mU-rA-rA |
| **MA3** | **GA3** | mU*fU*mA-mA-mU-fG-mC-fU-fA-mA-mU-mC-mG-fU-mG-fA-mU-mA-mG-mG-mG-mG*mU*mU | **PA3** | mC*mU*mC-mC-mU-mA-fC-mA-fU-fA-mU-mU-mA-mG-mC-mA-mU-mU-mA-mA |
| **MB1** | **GB1** | rU-rG-rG-rC-rA-rG-rU-rG-rU-rC-rU-rU-rA-rG-rC-rU-rG-rG-rU-rU-rG-rU | **PB1** | rG-rC-rA-rA-rU-rC-rA-rG-rC-rA-rA-rG-rU-rA-rU-rA-rC-rU-rG-rC-rC-rC |
| **MB2** | **GB2** | fU-rG-rG-fC-rA-rG-fU-rG-fU-fC-fU-fU-rA-rG-fC-fU-rG-rG-fU-fU*rG*fU | **PB2** | rA-rA-mU-mC-rA-rG-mC-rA-rA-rG-mU-rA-mU-rA-mC-mU-rG-mC-mC-mC |
| **MB3** | **GB3** | mU*fG*mG-mC-mA-fG-mU-fG-fU-mC-mU-mU-fA-mG-fC-mU-mG-mG-mU-mU*mG*mU | **PB3** | mA*mA*mU-mC-mA-mG-fC-fA-fA-fG-mU-mA-mU-mA-mC-mU-mG-mC-mC-mC |

## 5. SAR study results

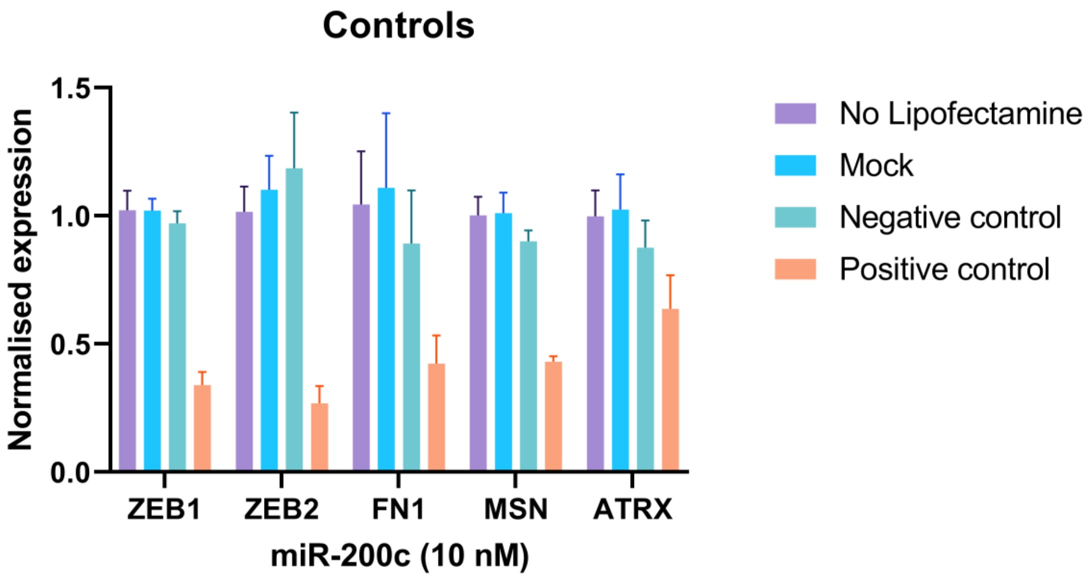


**Figure S6.** Knockdown (KD) data of controls conditions. A549 cells were transfected with 10 nM final concentration of mimics for 24 h, then analysed by RT-qPCR (n=3). Positive control was hsa-miR-200c-3p mimic from miRIDIAN®. Target mRNA expression was normalised to HPRT1 and baseline levels (control) were set to 1. Data shown as average with standard deviation.

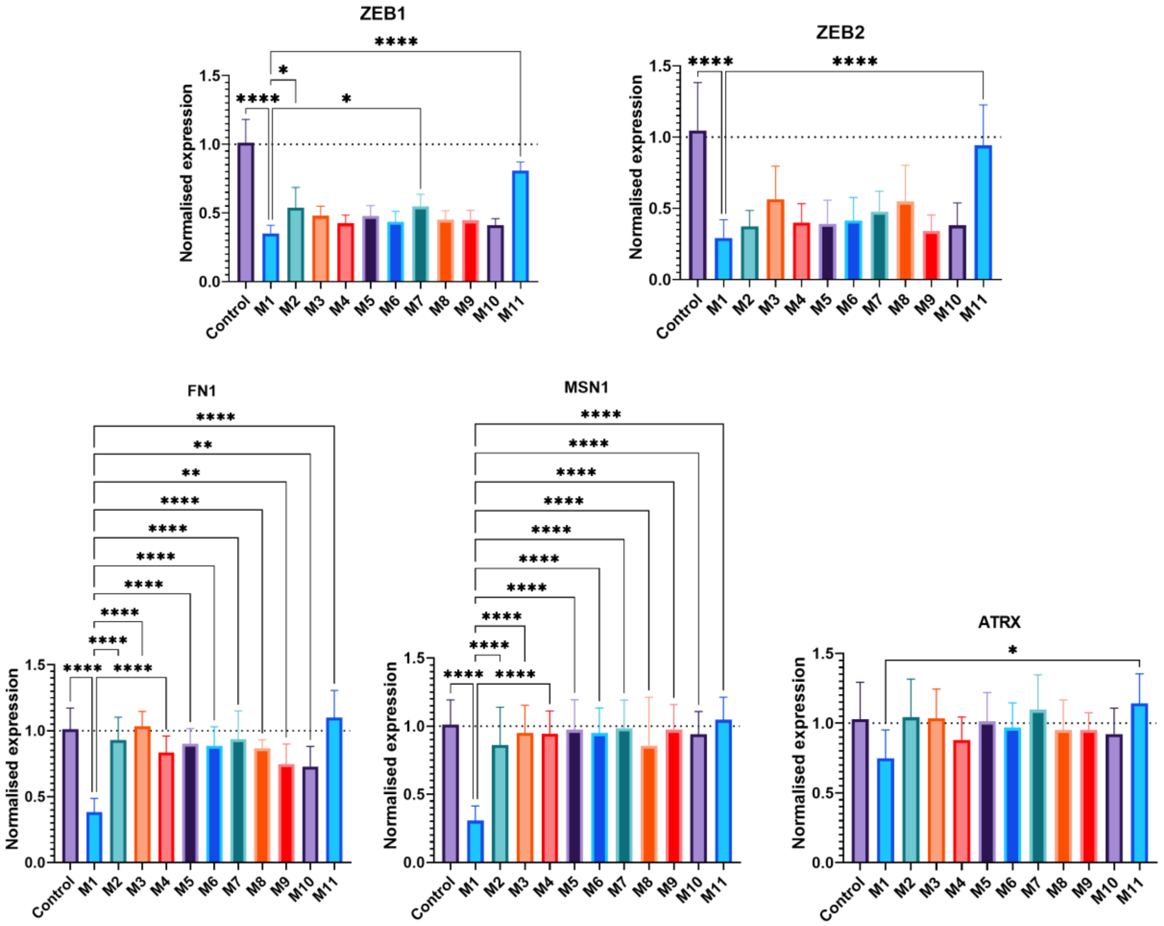


**Figure S7.** Knockdown (KD) data of the first series of miR-200c mimics. A549 cells were transfected with 10 nM final concentration of mimics for 24 h, then analysed by RT-qPCR (n=3). Target mRNA expression was normalised to HPRT1 and baseline levels (untreated control) were set to 1. Data shown as average with standard deviation. Statistical significance between the seris 1 mimics and the unmodified control (**M1**) are shown. *** p<0.05; ** p<0.01;*** p<0.001; **** p<0.0001**

**Table S3**: Series 1 mimics

| Compound | Average KD efficiency (%) | Significant from Untreated? | p-value (untreated vs compound) | Significant from M1? | p-value (M1 vs compound) |
|---|---|---|---|---|---|
| **ZEB1** | | | | | |
| M1 | 64.83 | Yes | <0.0001 | NA | NA |
| M2 | 46.17 | Yes | <0.0001 | Yes | 0.0185 |
| M3 | 52.00 | Yes | <0.0001 | No | 0.2302 |
| M4 | 57.5 | Yes | <0.0001 | No | 0.8729 |
| M5 | 52.17 | Yes | <0.0001 | No | 0.2437 |
| M6 | 56.33 | Yes | <0.0001 | No | 0.7365 |
| M7 | 45.33 | Yes | <0.0001 | Yes | 0.0119 |
| M8 | 55.00 | Yes | <0.0001 | No | 0.5571 |
| M9 | 55.33 | Yes | <0.0001 | No | 0.602 |
| M10 | 58.83 | Yes | <0.0001 | No | 0.9674 |
| M11 | 19.17 | Yes | 0.0035 | Yes | <0.0001 |
| **ZEB2** | | | | | |
| M1 | 70.83 | Yes | <0.0001 | NA | NA |
| M2 | 62.67 | Yes | <0.0001 | No | 0.9991 |
| M3 | 43.67 | Yes | 0.0038 | No | 0.365 |
| M4 | 60,00 | Yes | <0.0001 | No | 0.995 |
| M5 | 60.83 | Yes | <0.0001 | No | 0.9957 |
| M6 | 58.50 | Yes | <0.0001 | No | 0.9886 |
| M7 | 52.33 | Yes | 0.0003 | No | 0.8268 |
| M8 | 45.33 | Yes | 0.0024 | No | 0.4458 |
| M9 | 65.83 | Yes | <0.0001 | No | 0.9995 |
| M10 | 61.83 | Yes | <0.0001 | No | 0.999 |
| M11 | 5.67 | No | 0.9954 | Yes | <0.0001 |
| **FN1** | | | | | |
| M1 | 61.67 | Yes | <0.0001 | NA | NA |
| M2 | 6.99 | No | 0.9889 | Yes | <0.0001 |
| M3 | 0,00 | No | 0.9997 | Yes | <0.0001 |
| M4 | 16.67 | No | 0.3528 | Yes | <0.0001 |
| M5 | 10,00 | No | 0.8996 | Yes | <0.0001 |
| M6 | 11.5 | No | 0.7956 | Yes | <0.0001 |
| M7 | 6.49 | No | 0.9911 | Yes | <0.0001 |
| M8 | 13.33 | No | 0.6355 | Yes | <0.0001 |
| M9 | 25.5 | Yes | 0.0321 | Yes | 0.0021 |
| M10 | 27.17 | Yes | 0.0182 | Yes | 0.0039 |
| M11 | 0,00 | No | 0.9853 | Yes | <0.0001 |
| **MSN** | | | | | |
| M1 | 69.33 | Yes | <0.0001 | NA | NA |
| M2 | 13.83 | No | 0.8303 | Yes | <0.0001 |
| M3 | 5.00 | No | 0.9992 | Yes | <0.0001 |
| M4 | 5.67 | No | 0.9991 | Yes | <0.0001 |
| M5 | 2.67 | No | 0.9996 | Yes | <0.0001 |
| M6 | 5.17 | No | 0.9991 | Yes | <0.0001 |
| M7 | 1.5 | No | 0.9997 | Yes | <0.0001 |
| M8 | 14.33 | No | 0.799 | Yes | <0.0001 |
| M9 | 2.67 | No | 0.9996 | Yes | <0.0001 |
| M10 | 6.00 | No | 0.999 | Yes | <0.0001 |
| M11 | 0.00 | No | 0.9996 | Yes | <0.0001 |
| **ATRX** | | | | | |
| M1 | 25.33 | No | 0.1455 | NA | NA |
| M2 | 0.00 | No | 0.9999 | No | 0.1478 |
| M3 | 0.00 | No | >0.9999 | No | 0.1717 |
| M4 | 12.00 | No | 0.8855 | No | 0.9499 |
| M5 | 0.00 | No | 0.9998 | No | 0.2476 |
| M6 | 3.33 | No | 0.9993 | No | 0.4736 |
| M7 | 0.00 | No | 0.9992 | No | 0.0507 |
| M8 | 5,00 | No | 0.999 | No | 0.5774 |
| M9 | 5,00 | No | 0.999 | No | 0.5774 |
| M10 | 7.83 | No | 0.9888 | No | 0.758 |
| M11 | 0.00 | No | 0.9861 | Yes | 0.0172 |

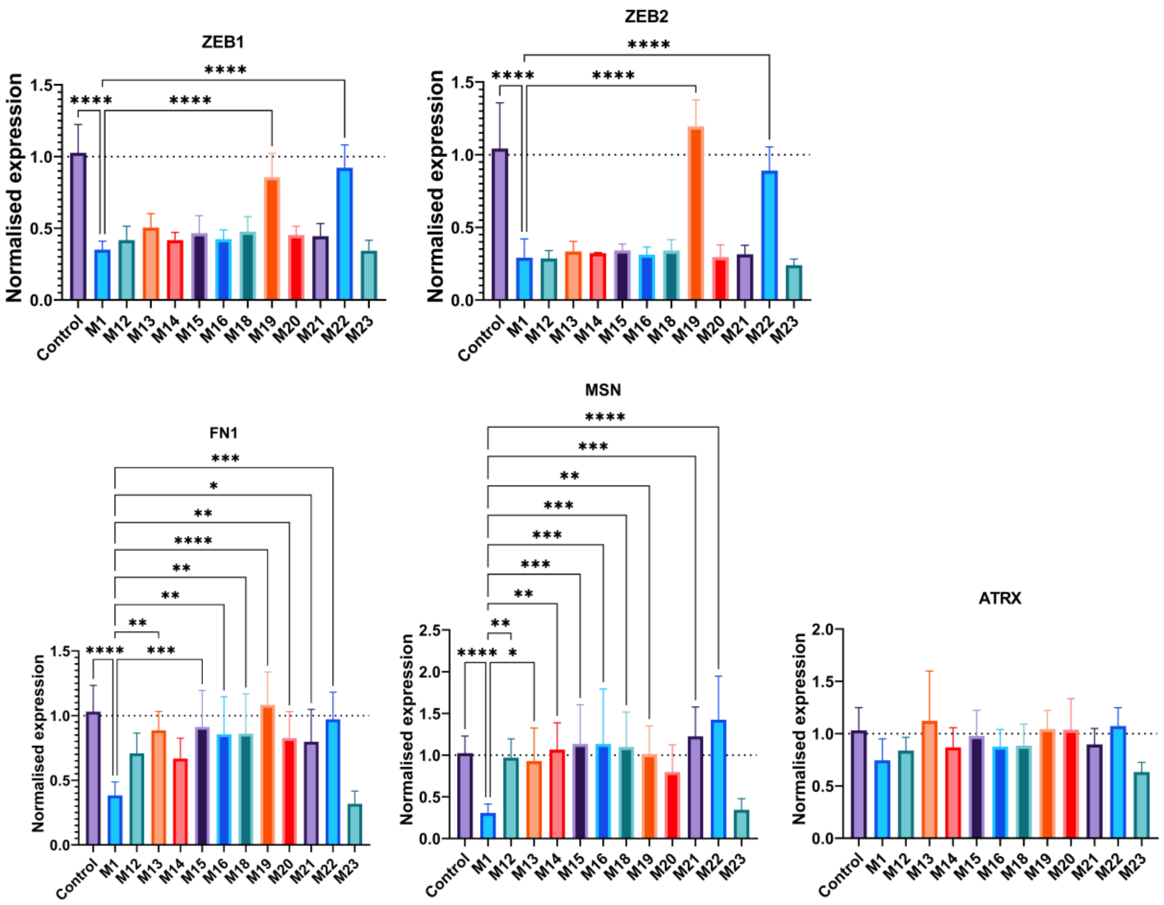


**Figure S8.** Knockdown (KD) data of the second series of miR-200c mimics. A549 cells were transfected with 10 nM final concentration of mimics for 24 h, then analysed by RT-qPCR (n=3). Target mRNA expression was normalised to HPRT1 and baseline levels (control) were set to 1. Data shown as average with standard deviation. Statistical significance between the seris 1 mimics and the unmodified control (**M1**) are shown. *** p<0.05; ** p<0.01;*** p<0.001; **** p<0.0001**

**Table S4**: Series 2 mimics

| Compound | Average KD efficiency (%) | Significant from Untreated? | p-value (untreated vs compound) | Significant from M1? | p-value (M1 vs compound) |
|---|---|---|---|---|---|
| **ZEB1** | | | | | |
| M1 | 64.83 | Yes | <0.0001 | NA | NA |
| M12 | 58.2 | Yes | <0.0001 | No | 0.9951 |
| M13 | 49.6 | Yes | <0.0001 | No | 0.4902 |
| M14 | 58.25 | Yes | <0.0001 | No | 0.9957 |
| M15 | 53.4 | Yes | <0.0001 | No | 0.8183 |
| M16 | 57.6 | Yes | <0.0001 | No | 0.9898 |
| M17 | 53.5 | Yes | <0.0001 | No | 0.2779 |
| M18 | 52.4 | Yes | <0.0001 | No | 0.737 |
| M19 | 14.4 | No | 0.2515 | Yes | <0.0001 |
| M20 | 54.8 | Yes | <0.0001 | No | 0.91 |
| M21 | 55.5 | Yes | <0.0001 | No | 0.9634 |
| M22 | 7.8 | No | 0.8661 | Yes | <0.0001 |
| **ZEB2** | | | | | |
| M1 | 70.83 | Yes | <0.0001 | NA | NA |
| M12 | 71.40 | Yes | <0.0001 | No | >0.9999 |
| M13 | 66.60 | Yes | <0.0001 | No | 0.9996 |
| M14 | 67.75 | Yes | <0.0001 | No | 0.9997 |
| M15 | 65.80 | Yes | <0.0001 | No | 0.9994 |
| M16 | 68.80 | Yes | <0.0001 | No | 0.9998 |
| M17 | 50.83 | Yes | <0.0001 | No | 0.478 |
| M18 | 66.00 | Yes | <0.0001 | No | 0.9994 |
| M19 | 0.00 | No | 0.8622 | Yes | <0.0001 |
| M20 | 70.40 | Yes | <0.0001 | No | >0.9999 |
| M21 | 68.50 | Yes | <0.0001 | No | 0.9997 |
| M22 | 11.00 | No | 0.8589 | Yes | <0.0001 |
| **FN1** | | | | | |
| M1 | 61.67 | Yes | <0.0001 | NA | NA |
| M12 | 29.20 | Yes | 0.0448 | Yes | 0.0964 |
| M13 | 11.40 | No | 0.8954 | Yes | 0.0014 |
| M14 | 33.25 | Yes | 0.0331 | No | 0.2623 |
| M15 | 8.80 | No | 0.9751 | Yes | 0.0006 |
| M16 | 14.40 | No | 0.7194 | Yes | 0.0031 |
| M17 | 9.33 | No | 0.9845 | Yes | <0.0001 |
| M18 | 14.00 | No | 0.7468 | Yes | 0.0028 |
| M19 | 0.00 | No | 0.9994 | Yes | <0.0001 |
| M20 | 17.40 | No | 0.5034 | Yes | 0.0068 |
| M21 | 20.25 | No | 0.4351 | Yes | 0.0252 |
| M22 | 2.80 | No | 0.9993 | Yes | 0.0001 |
| **MSN** | | | | | |
| M1 | 69.33 | Yes | <0.0001 | NA | NA |
| M12 | 3.00 | No | 0.9996 | Yes | 0.0059 |
| M13 | 7.20 | No | 0.9993 | Yes | 0.012 |
| M14 | 0.00 | No | 0.9997 | Yes | 0.0025 |
| M15 | 0.00 | No | 0.9991 | Yes | 0.0003 |
| M16 | 0.00 | No | 0.9991 | Yes | 0.0003 |
| M17 | 0.00 | No | 0.9989 | Yes | <0.0001 |
| M18 | 0.00 | No | 0.9994 | Yes | 0.0006 |
| M19 | 0.00 | No | >0.9999 | Yes | 0.0028 |
| M20 | 20.20 | No | 0.8597 | Yes | 0.0835 |
| M21 | 0.00 | No | 0.9605 | Yes | 0.0001 |
| M22 | 0.00 | No | 0.1533 | Yes | <0.0001 |
| **ATRX** | | | | | |
| M1 | 25.33 | No | 0.1455 | NA | NA |
| M12 | 16.20 | No | 0.7045 | No | 0.999 |
| M13 | 0.00 | No | 0.999 | No | 0.0672 |
| M14 | 13 | No | 0.9308 | No | 0.9895 |
| M15 | 2.20 | No | 0.9994 | No | 0.5456 |
| M16 | 12.40 | No | 0.9067 | No | 0.9791 |
| M17 | 8.33 | No | 0.9045 | No | 0.3367 |
| M18 | 11.60 | No | 0.9342 | No | 0.9662 |
| M19 | 0.00 | No | 0.9999 | No | 0.2422 |
| M20 | 0.00 | No | >0.9999 | No | 0.271 |
| M21 | 10.25 | No | 0.9846 | No | 0.958 |
| M22 | 0.00 | No | 0.9996 | No | 0.1536 |

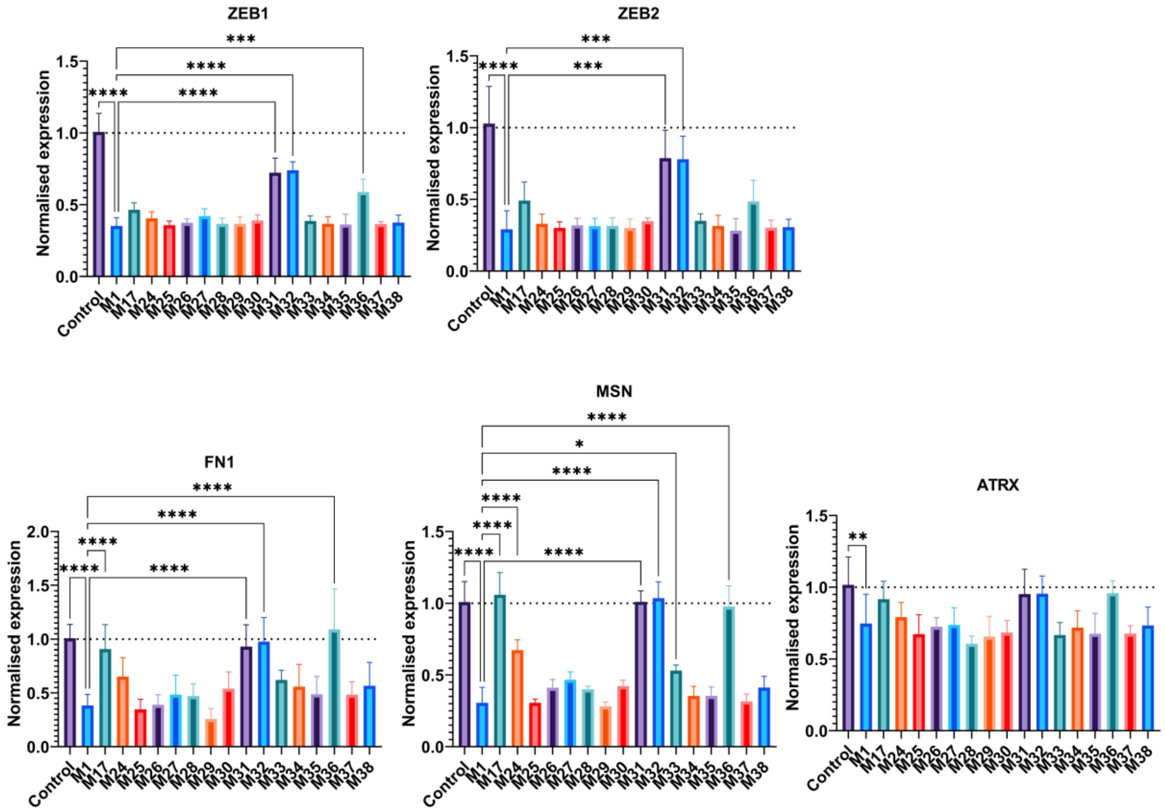


**Figure S9.** Knockdown (KD) data of the third series of miR-200c mimics. A549 cells were transfected with 10 nM final concentration of mimics for 24 h, then analysed by RT-qPCR (n=3). Target mRNA expression was normalised to HPRT1 and baseline levels (control) were set to 1. Data shown as average with standard deviation. Statistical significance between the seris 1 mimics and the unmodified control (**M1**) are shown. *** p<0.05; ** p<0.01;*** p<0.001; **** p<0.0001**

**Table S5**: Series 3 mimics

| Compound | Average KD efficiency (%) | Significant from Untreated? | p-value (untreated vs compound) | Significant from M1? | p-value (M1 vs compound) |
|---|---|---|---|---|---|
| **ZEB1** | | | | | |
| M1 | 64.83 | Yes | <0.0001 | NA | NA |
| M23 | 65.6 | Yes | <0.0001 | No | 0.9999 |
| M24 | 59.5 | Yes | <0.0001 | No | 0.9821 |
| M25 | 64.33 | Yes | <0.0001 | No | 0.9999 |
| M26 | 62.57 | Yes | <0.0001 | No | 0.9994 |
| M27 | 57.83 | Yes | <0.0001 | No | 0.8581 |
| M28 | 63.33 | Yes | <0.0001 | No | 0.9996 |
| M29 | 63.33 | Yes | <0.0001 | No | 0.9996 |
| M30 | 61.00 | Yes | <0.0001 | No | 0.9989 |
| M31 | 27.67 | Yes | <0.0001 | Yes | <0.0001 |
| M32 | 26.00 | Yes | <0.0001 | Yes | <0.0001 |
| M33 | 61.33 | Yes | <0.0001 | No | 0.999 |
| M34 | 63.33 | Yes | <0.0001 | No | 0.9996 |
| M35 | 63.83 | Yes | <0.0001 | No | 0.9997 |
| M36 | 41.17 | Yes | <0.0001 | Yes | 0.0002 |
| M37 | 63.4 | Yes | <0.0001 | No | 0.9997 |
| M38 | 62.4 | Yes | <0.0001 | No | 0.9994 |
| **ZEB2** | | | | | |
| M1 | 70.83 | Yes | <0.0001 | NA | NA |
| M23 | 76.00 | Yes | <0.0001 | No | 0.9994 |
| M24 | 67.00 | Yes | <0.0001 | No | 0.9995 |
| M25 | 69.83 | Yes | <0.0001 | No | 0.9999 |
| M26 | 68.14 | Yes | <0.0001 | No | 0.9997 |
| M27 | 68.67 | Yes | <0.0001 | No | 0.9997 |
| M28 | 68.50 | Yes | <0.0001 | No | 0.9997 |
| M29 | 69.83 | Yes | <0.0001 | No | 0.9999 |
| M30 | 65.20 | Yes | <0.0001 | No | 0.9993 |
| M31 | 21.33 | No | 0.1315 | Yes | 0.0002 |
| M32 | 22.00 | No | 0.1096 | Yes | 0.0003 |
| M33 | 65.00 | Yes | <0.0001 | No | 0.9992 |
| M34 | 68.50 | Yes | <0.0001 | No | 0.9997 |
| M35 | 71.83 | Yes | <0.0001 | No | 0.9999 |
| M36 | 51.50 | Yes | <0.0001 | No | 0.5239 |
| M37 | 69.80 | Yes | <0.0001 | No | 0.9999 |
| M38 | 69.40 | Yes | <0.0001 | No | 0.9999 |
| **FN1** | | | | | |
| M1 | 61.67 | Yes | <0.0001 | NA | NA |
| M23 | 68.20 | Yes | <0.0001 | No | 0.9992 |
| M24 | 34.83 | Yes | 0.0014 | No | 0.1109 |
| M25 | 65.33 | Yes | <0.0001 | No | 0.9995 |
| M26 | 61.00 | Yes | <0.0001 | No | >0.9999 |
| M27 | 51.67 | Yes | <0.0001 | No | 0.987 |
| M28 | 53.00 | Yes | <0.0001 | No | 0.9946 |
| M29 | 74.17 | Yes | <0.0001 | No | 0.9245 |
| M30 | 46.00 | Yes | <0.0001 | No | 0.7856 |
| M31 | 6.99 | No | 0.9955 | Yes | <0.0001 |
| M32 | 2.33 | No | 0.9996 | Yes | <0.0001 |
| M33 | 38.00 | Yes | 0.0003 | No | 0.2171 |
| M34 | 44.17 | Yes | <0.0001 | No | 0.595 |
| M35 | 51.33 | Yes | <0.0001 | No | 0.9847 |
| M36 | 0.00 | No | 0.995 | Yes | <0.0001 |
| M37 | 51.60 | Yes | <0.0001 | No | 0.9884 |
| M38 | 43.40 | Yes | <0,0001 | No | 0,6013 |
| **MSN** | | | | | |
| M1 | 69.33 | Yes | <0.0001 | NA | NA |
| M23 | 65.4 | Yes | 0.0008 | No | 0.9997 |
| M24 | 32.67 | Yes | <0.0001 | Yes | <0.0001 |
| M25 | 69.33 | Yes | <0.0001 | No | >0.9999 |
| M26 | 58.86 | Yes | <0.0001 | No | 0.7536 |
| M27 | 53.33 | Yes | <0.0001 | No | 0.2635 |
| M28 | 60.00 | Yes | <0.0001 | No | 0.8912 |
| M29 | 71.83 | Yes | <0.0001 | No | 0.9996 |
| M30 | 57.80 | Yes | <0.0001 | No | 0.7407 |
| M31 | 0.00 | No | >0.9999 | Yes | <0.0001 |
| M32 | 0.00 | No | 0.9995 | Yes | <0.0001 |
| M33 | 46.83 | Yes | <0.0001 | Yes | 0.0314 |
| M34 | 64.50 | Yes | <0.0001 | No | 0.999 |
| M35 | 64.50 | Yes | <0.0001 | No | 0.999 |
| M36 | 2.33 | No | 0.9994 | Yes | <0.0001 |
| M37 | 68.40 | Yes | <0.0001 | No | 0.9999 |
| M38 | 58.80 | Yes | <0.0001 | No | 0.8336 |
| **ATRX** | | | | | |
| M1 | 25.33 | No | 0.1455 | NA | NA |
| M23 | 36.60 | Yes | 0.0147 | No | 0.9903 |
| M24 | 20.83 | Yes | 0.0263 | No | 0.9992 |
| M25 | 32.67 | Yes | <0.0001 | No | 0.9898 |
| M26 | 27.57 | Yes | 0.0004 | No | 0.9996 |
| M27 | 26.33 | Yes | 0.0018 | No | 0.9999 |
| M28 | 39.33 | Yes | <0.0001 | No | 0.5928 |
| M29 | 34.5 | Yes | <0.0001 | No | 0.9601 |
| M30 | 31.60 | Yes | 0.0003 | No | 0.9959 |
| M31 | 4.83 | No | 0.9951 | No | 0.1436 |
| M32 | 4.50 | No | 0.9957 | No | 0.1312 |
| M33 | 33.33 | Yes | <0.0001 | No | 0.9869 |
| M34 | 28.17 | Yes | 0.0007 | No | 0.9995 |
| M35 | 32.50 | Yes | <0.0001 | No | 0.9906 |
| M36 | 4.17 | No | 0.9989 | No | 0.1196 |
| M37 | 32.40 | Yes | 0.0002 | No | 0.9948 |
| M38 | 26.60 | Yes | 0.0039 | No | 0.9998 |

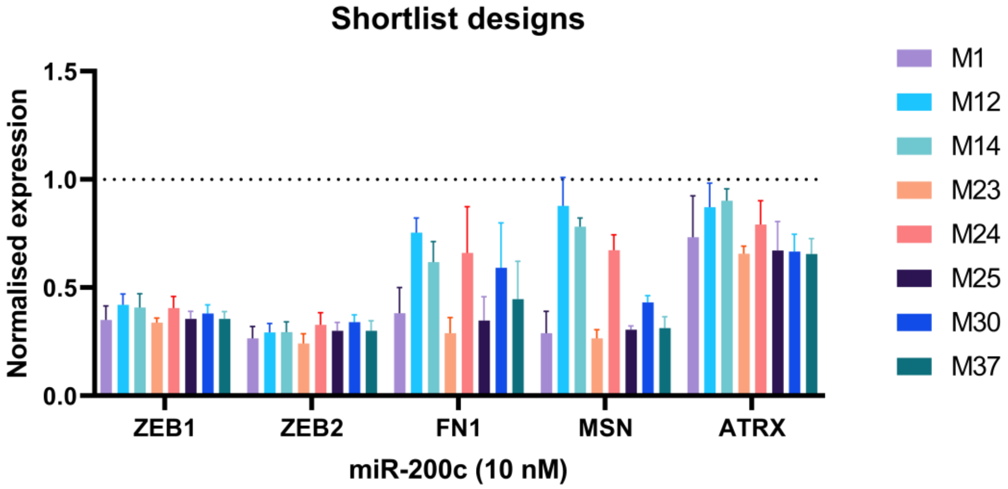


**Figure S10.** Knockdown (KD) data of shortlist designs of miR-200c mimics. A549 cells were transfected with 10 nM final concentration of mimics for 24 h, then analysed by RT-qPCR (n=3). Target mRNA expression was normalised to HPRT1 and baseline levels (control) were set to 1. Data shown as average with standard deviation.

## 6. Luciferase dual reporter assay

*Reporter Design*

Reporter construct design was based on the GenBank sequences of both targets FN1 and ZEB1 (NM_001323661.2 and NM_001306129.2) and the psiCHECK2B vector. The psiCHECK2B vector was generated by replacing the original multiple cloning site (MCS) of psiCHECK2 (AY535007.1, Promega, Madison, USA) with a modified version. The new MCS carried an additional SpeI recognition site between the XhoI and PmeI recognition sites. The insert sequences were trimmed of any remaining part of the poly-A tail. For the reporter plasmid containing FN1, restriction sites XhoI, together with SpeI were added for the 5' ligation and NotI for the 3' ligation of the insert. For the reporter construct containing ZEB1, only XhoI was added for the 5' ligation and NotI for the 3' ligation. Insert sequences were chemically synthesized and cloned into the psiCHECK2B vector by GeneArt (Regensburg, Germany).

Plasmid: **V0212**
Backbone: psiCHECK2B
Cloning sites: 5'- XhoI
3'- NotI
Gene: human NM_001323661 Homo sapiens ZEB1 zinc finger E-box binding homeobox 1, full
Comments: Gene synthesis and cloning by GeneArt
Insert length: 2,564 bp
Insert sequence:

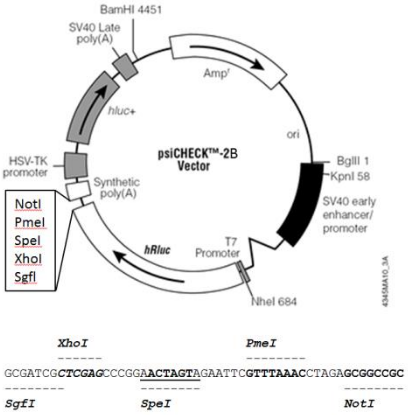


> ref| NM_001323661 | Homo sapiens ZEB1 zinc finger E-box binding homeobox 1| XhoI, NotI, 2,564 bp

CTCGAGTCGTTTTTCTAGAAGGAAAATAAATTCTAATTGATAATGAATTTCGTTCAATATTATCCTTGCTTTTCATGGAAACACAGTAACCTGT
ATGCTGTGATTCCTGTTCACTACTGTGTAAAGTAAAAACTAAAAAAATACAAAATACAAAACACACACACACACACACACACACACACACAC
ACACACAAAATAAATCCGGGTGTGCCTGAACCTCAGACCTAGTAATTTTTCATGCAGTTTTCAAAGTTAGGAACAAGTTTGTAACATGCAGCAG
ATTAGAAAACCTTAATGACTCAGAGAGCAACAATACAAGAGGTTAAAGGAAGCTGATTAATTAGATATGCATCTGGCATTGTTTTATCTTATCA
GTATTATCACTCTTATGTTGGTTTATTCTTAAGCTGTACAATTGGGAGAAATTTTATAATTTTTTATTGGTAAACATATGCTAAATCCGCTTCA
GTATTTTATTATGTTTTTTAAAATGTGAGAACTTCTGCACTACAAAATTCCCTTCACAGAGAAGTATAATGTAGTTCCAACCCGTGCTAACTAC
CTTTTATAAATTCAGTCTAGAAGGTAGTAATTTCTAATATTTAGATGTCTTAGTAGAGCGTATTATCATTTAAAGTGTATTGTTAGCCTTAAGA
AAGCAGCTGATAGAAGAACTGAAGTTTCTTACTCACGTGGTTTAAAATGGAGTTCAAAAGATTGCCATTGAGTTCTGATTGCAGGGACTAACAA
TGTTAATCTGATAAGGACAGCAAAATCATCAGAATCAGTGTTTGTGATTGTGTTTGAATATGTGGTAACATATGAAGGATATGACATGAAGCTT
TGTATCTCCTTTGGCCTTAAGCAAGACCTGTGTGCTGTAAGTGCCATTTCTCAGTATTTTCAAGGCTCTAACCCGCCTTCATCCAATGTGTGGC
CTACAATAACTAGCATTTGTTGATTTGTCTCTTGTATCAAAATTCCCAAATAAAACTTAAAACCACTGACTCTGTCAGAGAAACTGAAACACTG
GGACATTTCATCCTTCAATTCCTCGGTATTGATTTTATGTTGATTGATTTTCAGAATTTCTCTACAGAAACGAAAGGGAAATTTTCTAATCTGC
TTTATCCATGTACTTGCATTTCAGACATGGACATGCTATTGTTATTTGGCTCATAACTGTTTCCAAATGTTAGTTATTATGGACCCAATTTATT
AACAACATTAGCTGATTTTTACCTATCAGTATTATTTTATTTCTTTTAGTTTATAGATCTGTGCAACATTTTTGTACTGTATGTCTTCAAACCT
GGCAGTATTAATACCCTTCTTACTGACATATGTACTTTTAGTTTTAGAAAACTTTTATATTTATGTGTCTTATTTTTATATTTCTTTATTTATT
ACACAGTGTAGTGTATAATACTGTAGTTTGTATTAATACAATAATATATTTTAGTATGAAAATTTGGAAAGTTGATAAGATTTAAAGTAGAGAT
GCAATTGGTTCTCCTGCATTGAGATTTGATTTAACAGTGTTATGTTAACATTTATACTTGCCTTGGACTGTAGAACAGAACTTAAATGGGAATG
TATTAGTTTTACAACTACAATCAAGTCATTTTACCTTTACCCAGTTTTTAATATAAAACTTAAATTTTGAAATTCACTGTGTGACTAATAGCAT
GATGCTCTGCAGTTTTATTAAGAAATCAGCCTAACCATACAACTCTCATTTCCTTAGTAAGCCAAATTAGGATTAACTTCTATAAACAGTGTTG

GGAACAATGTTTAACATTTTGTGCCAATTTGTTCCTGTATTCATGTATGTAAGTTACAGATCTGACTCTTCATTTTTAAGTTCCTTGTTACATC
ATGGTCATTTTCTAGTTTTTTACCAGACTCCCATCTCACAATAAAATGCATCAACAAGCCTGAACTGCTGTCATTCTTTTCATCATTATCAGTA
TTTTCTTTGGAAAACTGTGAAATGGGGTACATTGTCATCCTGCATTTGATTCATCTTGAGCTGAATTTGGGTAACACTAAATGTTTTAGACATT
CTCCACTAAATTATGGATTTTCTTGTGGCTAAATGTTTCTGGAGAGGTCAGAGTTGACAAAACCTCTTCACAGGTTGCTCCTTCTTCCTGAAAT
CCTTAATCCTCCGCATTTCATGCTTCAGGTCATTTCAGGGAAGCCTGGGTTTAGATGCCTTTCTGACTCTCAGCTCCTGCACTTCTGTCATCAT
ACCTCTGATACTATTATTTATATTCCTTCCCCACTAGGAACAGGAACCACATTTGTCATAGTCACTCTCACATTCCTCACTGCCTAACAGGGTG
CCTGGCATAAGTTGGGACAACAGATATTTGTTGAATAAAAATATAATTTGCATGTTTATGGAGCTCAGCTATGTTCTCACTTTTTTTGCTTCTA
ATTCCAGAATATATGTTAAATGATCTAATAATTTGATTATTTTCTTATAAGTCTTATTAAACACTAGTCATAATAGACACAATAAATTATGCCT
TCTTTTTCTATTGCCTTAGCGGCCGC

| Name | Sequence | Length | Overhang | Frequency | Cut position |
|---|---|---|---|---|---|
| XhoI | CTCGAG | 6 | 5-prime | 1 | 1 |
| NotI | GCGGCCGC | 8 | 5-prime | 1 | 2557 |

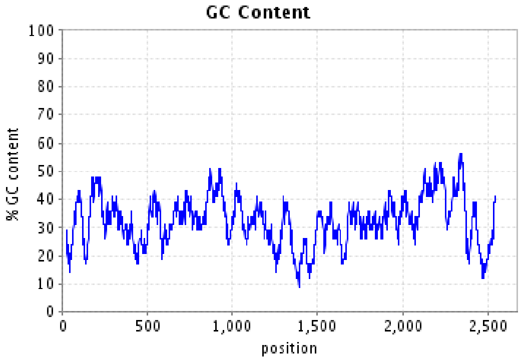


Plasmid: **V0213**
Backbone: psiCHECK2B
Cloning sites: 5'- XhoI, SpeI
3'- NotI
Gene: human NM_001306129, Homo sapiens fibronectin 1 (FN1), transcript variant 8, full
Comments: Gene synthesis and cloning by GeneArt
Insert length: 1,139 bp
Insert sequence:

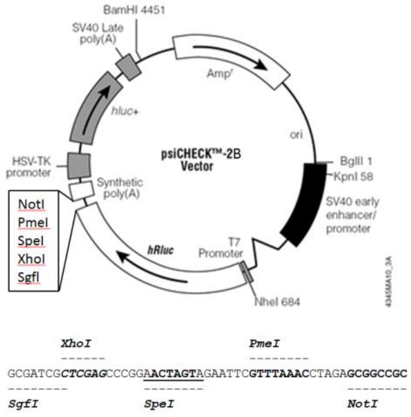


> ref| NM_001306129| Homo sapiens fibronectin 1 (FN1), transcript variant 8, mRNA| XhoI/SpeI, NotI, 1,139 bp
CTCGAGACTAGTATCATCTTTCCAATCCAGAGGAACAAGCATGTCTCTCTGCCAAGATCCATCTAAACTGGAGTGATGTTAGCAGACCCAGCTT
AGAGTTCTTCTTTCTTTCTTAAGCCCTTTGCTCTGGAGGAAGTTCTCCAGCTTCAGCTCAACTCACAGCTTCTCCAAGCATCACCCTGGGAGTT
TCCTGAGGGTTTTCTCATAAATGAGGGCTGCACATTGCCTGTTCTGCTTCGAAGTATTCAATACCGCTCAGTATTTTAAATGAAGTGATTCTAA
GATTTGGTTTGGGATCAATAGGAAAGCATATGCAGCCAACCAAGATGCAAATGTTTTGAAATGATATGACCAAAATTTTAAGTAGGAAAGTCAC
CCAAACACTTCTGCTTTCACTTAAGTGTCTGGCCCGCAATACTGTAGGAACAAGCATGATCTTGTTACTGTGATATTTTAAATATCCACAGTAC
TCACTTTTTCCAAATGATCCTAGTAATTGCCTAGAAATATCTTTCTCTTACCTGTTATTTATCAATTTTTCCCAGTATTTTTATACGGAAAAAA
TTGTATTGAAAACACTTAGTATGCAGTTGATAAGAGGAATTTGGTATAATTATGGTGGGTGATTATTTTTTATACTGTATGTGCCAAAGCTTTA
CTACTGTGGAAAGACAACTGTTTTAATAAAAGATTTACATTCCACAACTTGAAGTTCATCTATTTGATATAAGACACCTTCGGGGGAAATAATT
CCTGTGAATATTCTTTTTCAATTCAGCAAACATTTGAAAATCTATGATGTGCAAGTCTAATTGTTGATTTCAGTACAAGATTTTCTAAATCAGT
TGCTACAAAAACTGATTGGTTTTTGTCACTTCATCTCTTCACTAATGGAGATAGCTTTACACTTTCTGCTTTAATAGATTTAAGTGGACCCCAA
TATTTATTAAAATTGCTAGTTTACCGTTCAGAAGTATAATAGAAATAATCTTTAGTTGCTCTTTTCTAACCATTGTAATTCTTCCCTTCTTCCC

TCCACCTTTCCTTCATTGAATAAACCTCTGTTCAAAGAGATTGCCTGCAAGGGAAATAAAAATGACTAAGATATTAAAAGTATTTGAATAGTAA
AAAGCGGCCGC

| Name | Sequence | Length | Overhang | Frequency | Cut position |
|---|---|---|---|---|---|
| XhoI | CTCGAG | 6 | 5-prime | 1 | 1 |
| SpeI | ACTAGT | 6 | 5-prime | 1 | 7 |
| NotI | GCGGCCGC | 8 | 5-prime | 1 | 1132 |

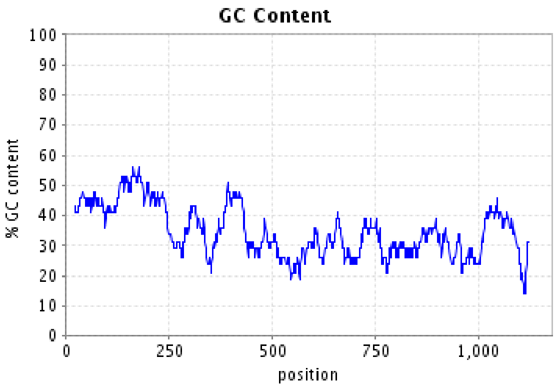

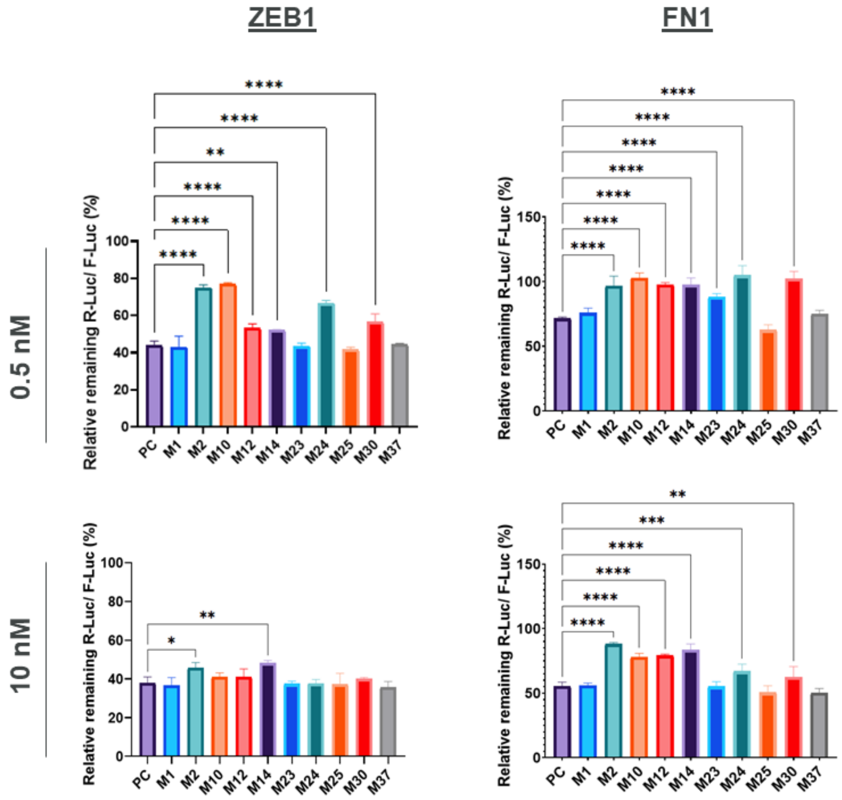


**Figure S11.** Statistical analysis of luciferase assay. Data shown as average with standard deviation. Statistical significance between the selected mimics and the positive control (PC; commerical mimic) are shown. No statisitcal significance indicates that the mimic has activity similar to that of the postive control. *** p<0.05; ** p<0.01;*** p<0.001; **** p<0.0001**

## 7. Stability

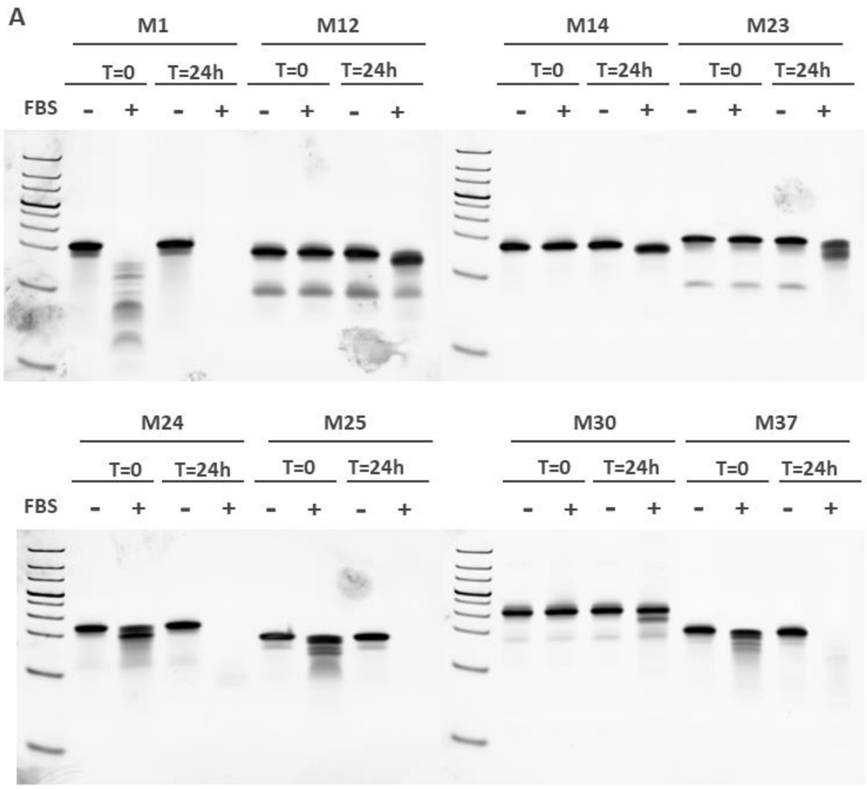


**Figure S12**: Stability of designs selected for further analyses. Selected miRNA mimic designs were analysed for stability in FBS (+) or PBS (-) over 24 hrs.

## 8. RNA-sequencing

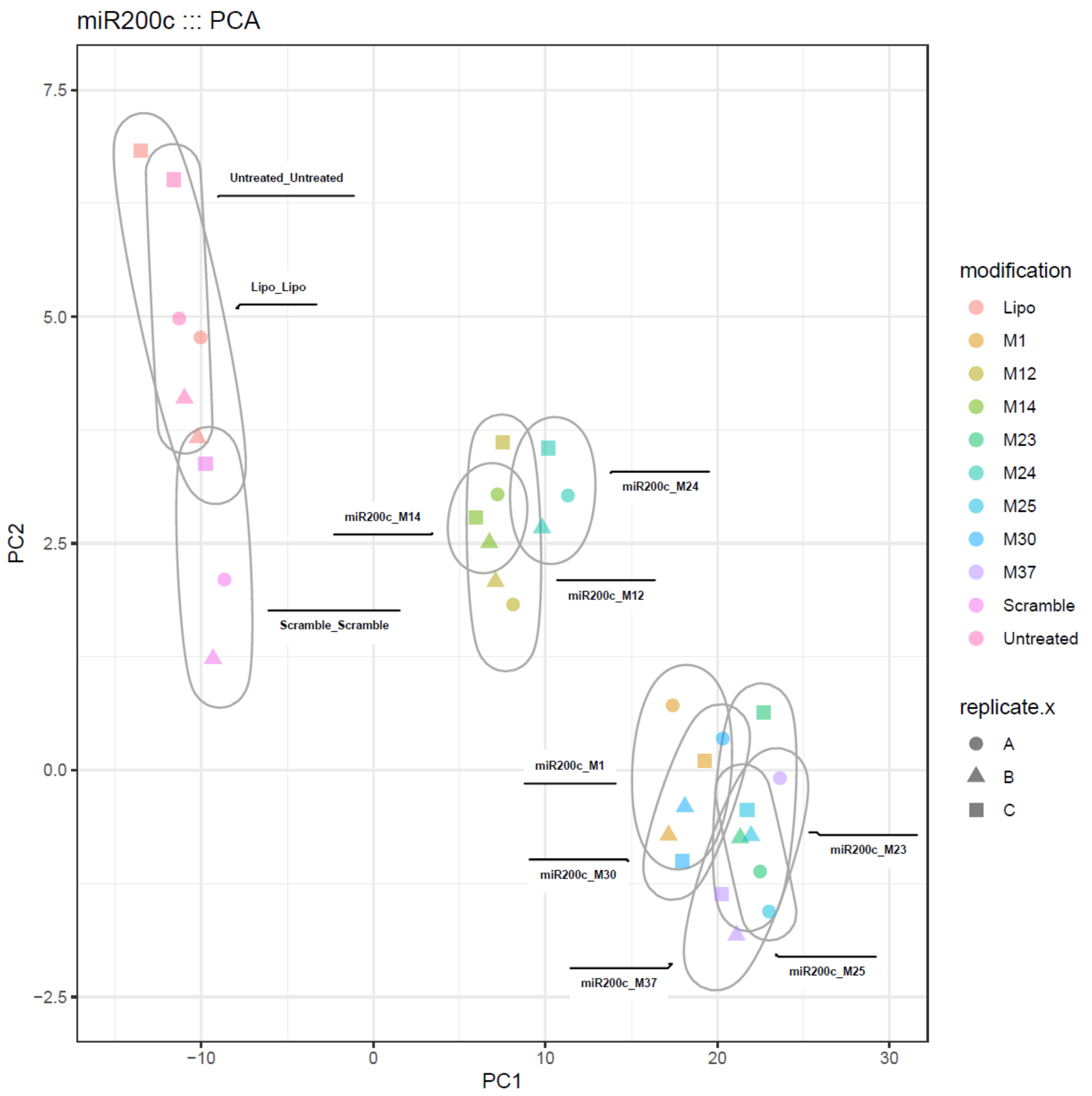


**Figure S13**. PCA plot with top 8 candidate mimics from RNA-seq data. *A, B, C* indicates different biological replicates performed.

**Table S6**: Number of downregulated DEGs in selected mimics compared to untreated sample with differing mismatches in the antisense or sense sequences

| Downregulated | | | | | | | | |
|---|---|---|---|---|---|---|---|---|
| mimic | M1 | M12 | M14 | M23 | M24 | M25 | M30 | M37 |
| | No Mismatches | No Mismatches | No Mismatches | No Mismatches | No Mismatches | No Mismatches | No Mismatches | No Mismatches |
| Antisense | 40 | 31 | 30 | 48 | 39 | 47 | 43 | 49 |
| Sense | 14 | 8 | 6 | 11 | 12 | 12 | 10 | 9 |
| | | | | | | | | |
| mimic | M1 | M12 | M14 | M23 | M24 | M25 | M30 | M37 |
| | 1 Mismatch | 1 Mismatch | 1 Mismatch | 1 Mismatch | 1 Mismatch | 1 Mismatch | 1 Mismatch | 1 Mismatch |
| Antisense | 131 | 66 | 69 | 174 | 74 | 171 | 119 | 150 |
| Sense | 107 | 46 | 58 | 144 | 68 | 143 | 96 | 125 |
| | | | | | | | | |
| mimic | M1 | M12 | M14 | M23 | M24 | M25 | M30 | M37 |
| | 2 Mismatches | 2 Mismatches | 2 Mismatches | 2 Mismatches | 2 Mismatches | 2 Mismatches | 2 Mismatches | 2 Mismatches |
| Antisense | 234 | 191 | 171 | 270 | 241 | 278 | 247 | 272 |
| Sense | 232 | 189 | 167 | 264 | 234 | 269 | 241 | 266 |

## 9. Translatability of mimic designs

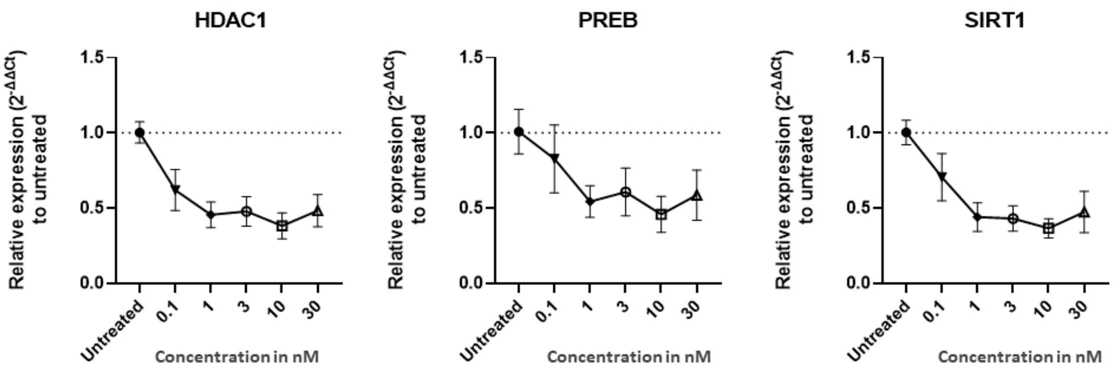


**Figure S14**: Concentration curves with miRVANA commercial miR-34a mimic (n=3).

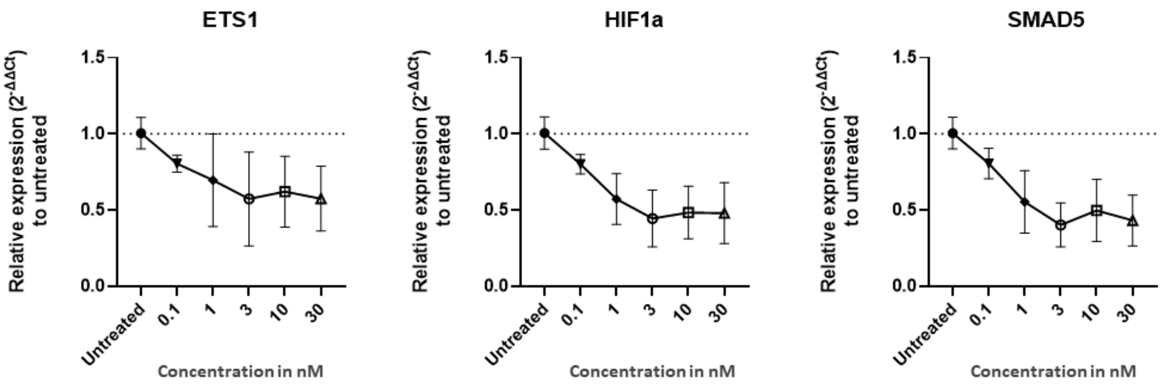


**Figure S15**: Concentration curves with miRVANA commercial miR-155 mimic (n=3).

**Table S7**: Knock-down of translatable mimic designs: miR-155.

| | | | |
|---|---|---|---|
| ETS1 | | | |
| Compound | KD efficiency | Significant from Untreated? | p-value (untreated vs compound) |
| MA1 | 43.68 | Yes | <0.0001 |
| MA2 | 44.02 | Yes | <0.0001 |
| MA3 | 17.46 | Yes | 0.0024 |
| | | | |
| HIF1a | | | |
| Compound | KD efficiency | Significant from Untreated? | p-value (untreated vs compound) |
| MA1 | 55.96 | Yes | <0.0001 |
| MA2 | 65.91 | Yes | <0.0001 |
| MA3 | 48.8 | Yes | <0.0001 |
| | | | |
| SMAD5 | | | |
| Compound | KD efficiency | Significant from Untreated? | p-value (untreated vs compound) |
| MA1 | 58.77 | Yes | <0.0001 |
| MA2 | 60.32 | Yes | <0.0001 |
| MA3 | 50.11 | Yes | <0.0001 |
| | | | |
| *** any KD efficient value less than zero was set to 0 | | | |

**Table S8**: Knock-down of translatable mimic designs: miR-34a.

| | | | | |
|---|---|---|---|---|
| HDAC | | | | |
| | Compound | KD efficiency | Significant from Untreated? | p-value (untreated vs compound) |
| | MB1 | 36.49 | Yes | 0.0294 |
| | MB2 | 0.00 | No | 0.9546 |
| | MB3 | 41.5 | Yes | 0.0106 |
| | | | | |
| PREB | Compound | KD efficiency | Significant from Untreated? | p-value (untreated vs compound) |
| | MB1 | 23.83 | No | 0.5629 |
| | MB2 | 0,00 | Yes | 0.0268 |
| | MB3 | 23.52 | No | 0.5744 |
| | | | | |
| SIRT1 | | | | |
| | Compound | KD efficiency | Significant from Untreated? | p-value (untreated vs compound) |
| | MB1 | 48.28 | Yes | 0.0238 |
| | MB2 | 0,00 | No | 0.932 |
| | MB3 | 43.03 | No | 0.051 |
| | | | | |
| | *** any KD efficient value less than zero was set to 0 | | | |

### 10. LCMS data

*Guide strands*

5’ - rU-rA-rA-rU-rA-rC-rU-rG-rC-rC-rG-rG-rG-rU-rA-rA-rU-rG-rA-rU-rG-rG-rA (**G1**)

| Item name | Observed RT (min) | Neutral mass (Da) | Observed neutral mass (Da) | Observed m/z | Mass error (mDa) | Mass error (ppm) |
|---|---|---|---|---|---|---|
| EN12762-73 | 4.62 | 7408.02 | 7407.9780 | 1850.9872 | -41.6 | -5.6 |

Channel name: TUV 260 : Integrated : Smoothed

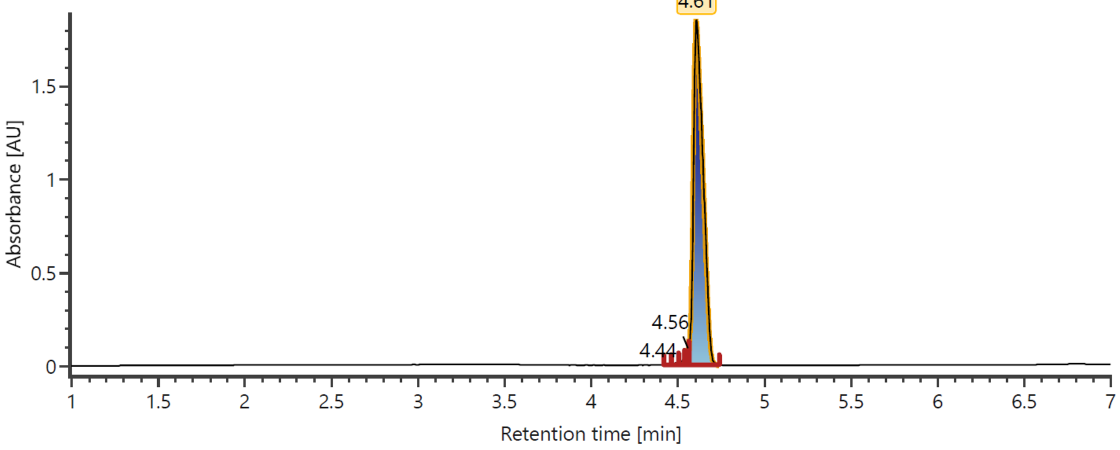


| . | Retention time (min) | Area | % Area (%) | Height |
|---|---|---|---|---|
| 1 | 4.61 | 7179339 | 98.34 | 1848999 |

**Item name: EN12762-73**
**Channel name: 1: TOF MS (400-5000) -40V ESI- (TIC)**

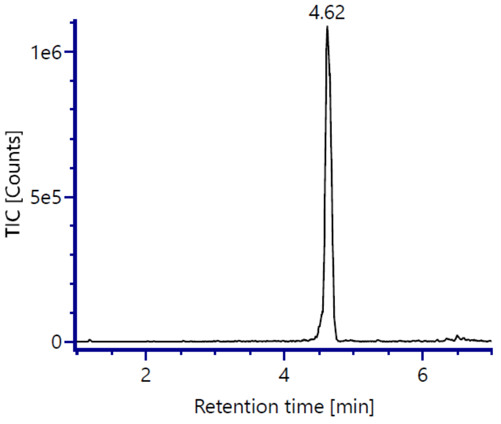


Item description: Channel name: Time 4.6219 +/- 0.0173 minutes

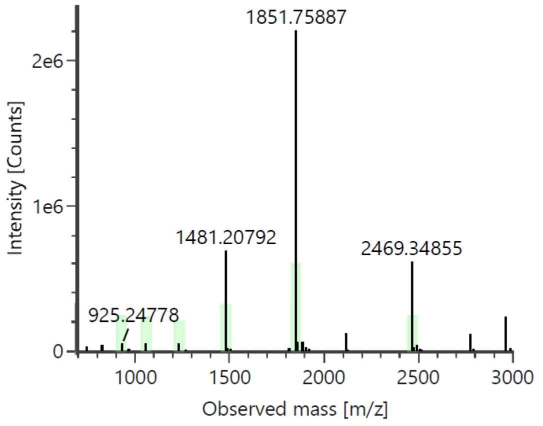

5’ - mU-fA-mA-fU-mA-fC-mU-fG-mC-fC-mG-fG-mG-fU-mA-fA-mU-fG-mA-fU-mG-fG-mA (**G2**)

| Item name | Observed RT (min) | Neutral mass (Da) | Observed neutral mass (Da) | Observed m/z | Mass error (mDa) | Mass error (ppm) |
|---|---|---|---|---|---|---|
| EN12762-33-003 | 4.67 | 7598.16 | 7598.1109 | 1898.5204 | -48.8 | -6.4 |

Channel name: TUV 260 : Integrated : Smoothed

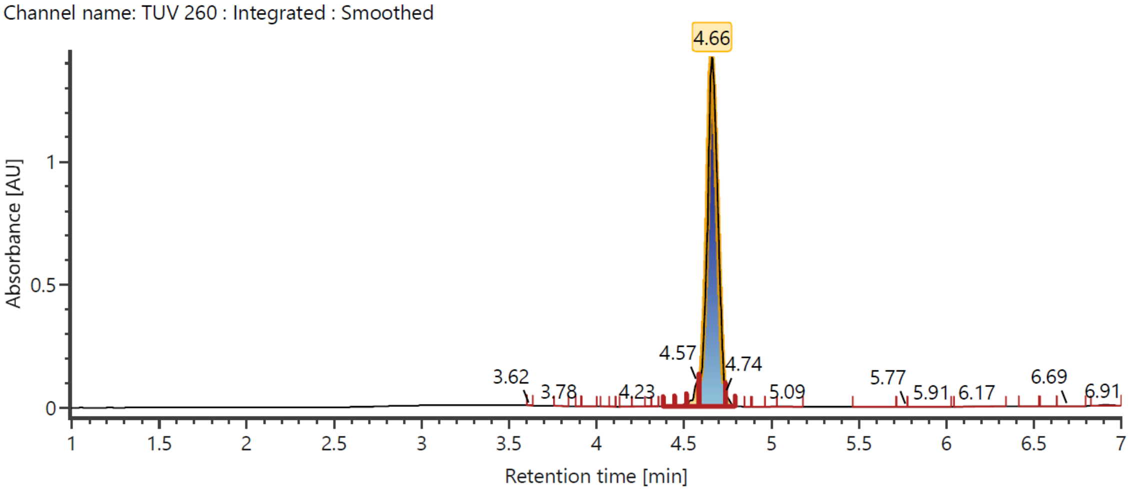


| . | Retention time (min) | Area | % Area (%) | Height |
|---|---|---|---|---|
| 1 | 4.66 | 5915860 | 94.66 | 1417958 |

**Item name: EN12762-33-003**
**Channel name: 1: TOF MS (400-5000) -40V ESI- (TIC)**

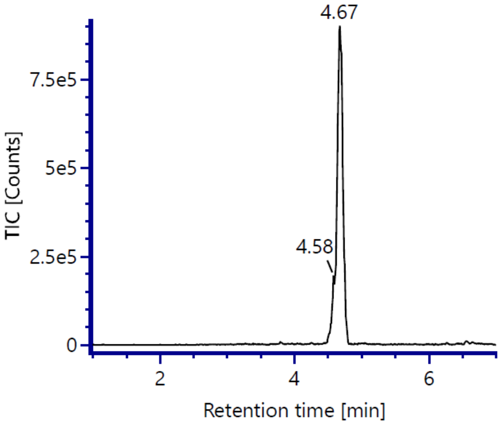


Item description: Channel name: Time 4.6687 +/- 0.0167 minutes

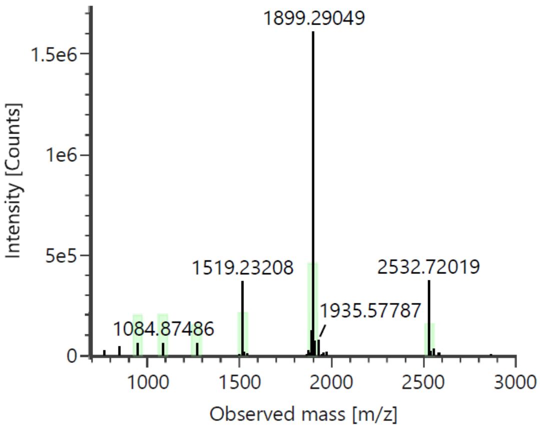

5’ - fU-mA-fA-mU-fA-mC-fU-mG-fC-mC-fG-mG-fG-mU-fA-mA-fU-mG-fA-mU-fG-mG-fA (**G3**)

| Item name | Observed RT (min) | Neutral mass (Da) | Observed neutral mass (Da) | Observed m/z | Mass error (mDa) | Mass error (ppm) |
|---|---|---|---|---|---|---|
| EN12762-33-002 | 4.70 | 7586.14 | 7586.0722 | 1895.5108 | -67.5 | -8.9 |

Channel name: TUV 260 : Integrated : Smoothed

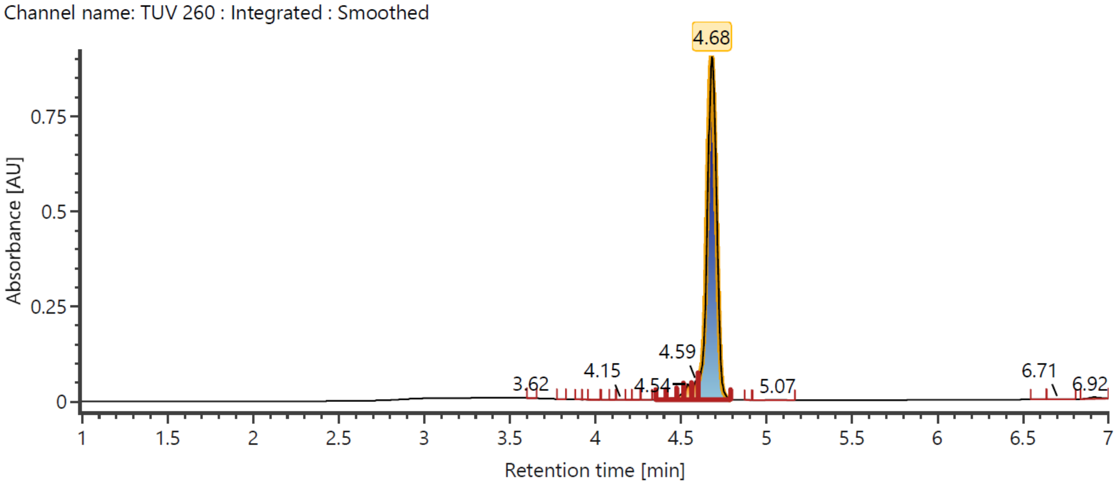


| . | Retention time (min) | Area | % Area (%) | Height |
|---|---|---|---|---|
| 1 | 4.68 | 3435844 | 93.55 | 901451 |

**Item name: EN12762-33-002**
**Channel name: 1: TOF MS (400-5000) -40V ESI- (TIC)**

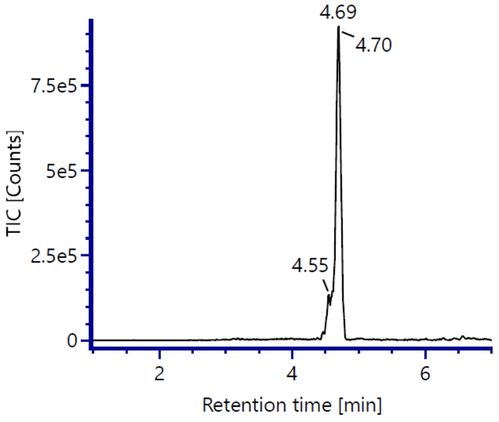


Item description: Channel name: Time 4.6980 +/- 0.0178 minutes

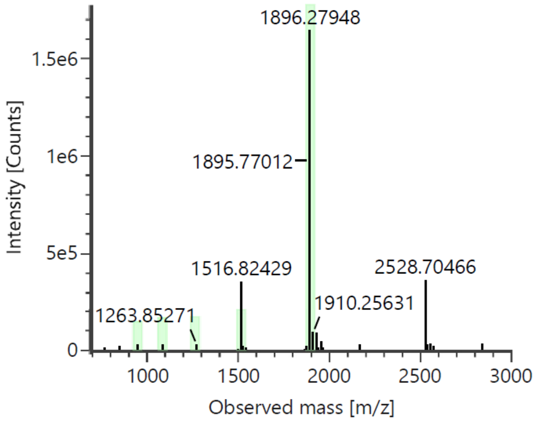

5’ - P-mU-fA-mA-fU-mA-fC-mU-fG-mC-fC-mG-fG-mG-fU-mA-fA-mU-fG-mA-fU-mG-fG-mA (**G4**)

| Item name | Observed RT (min) | Neutral mass (Da) | Observed neutral mass (Da) | Observed m/z | Mass error (mDa) | Mass error (ppm) |
|---|---|---|---|---|---|---|
| EN12762-33-004 | 4.72 | 7678.13 | 7678.0793 | 1918.5125 | -46.8 | -6.1 |

Channel name: TUV 260 : Integrated : Smoothed

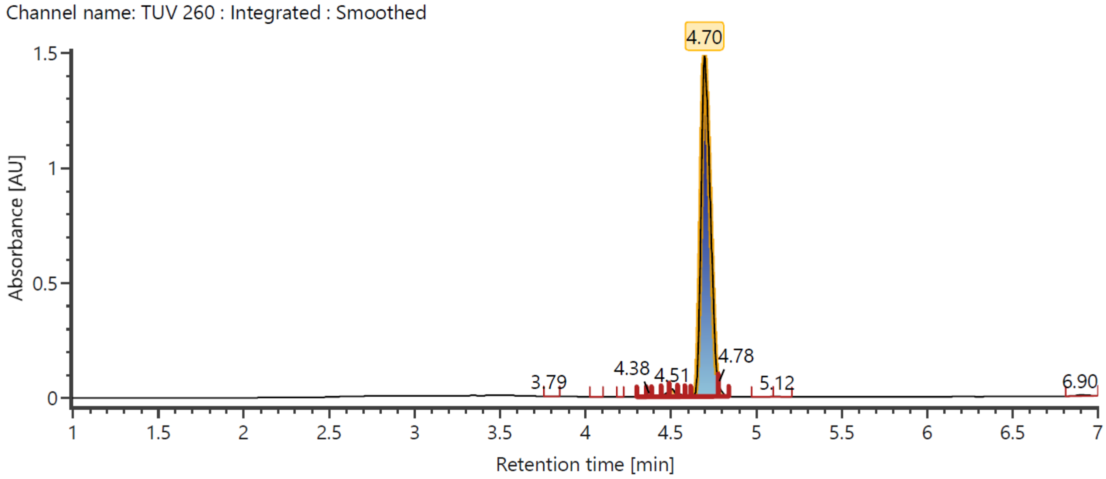


| . | Retention time (min) | Area | % Area (%) | Height |
|---|---|---|---|---|
| 1 | 4.70 | 5564547 | 96.05 | 1480724 |

**Item name: EN12762-33-004**
**Channel name: 1: TOF MS (400-5000) -40V ESI- (TIC)**

Item description:

Channel name: Time 4.7151 +/- 0.0188 minutes

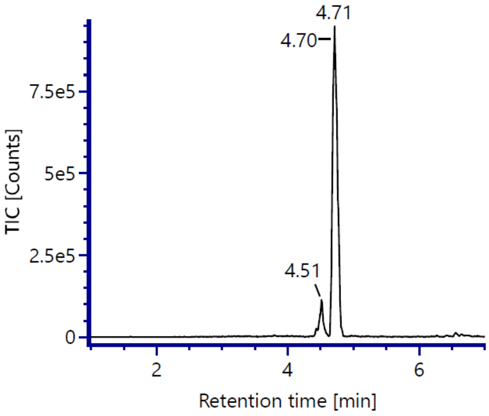


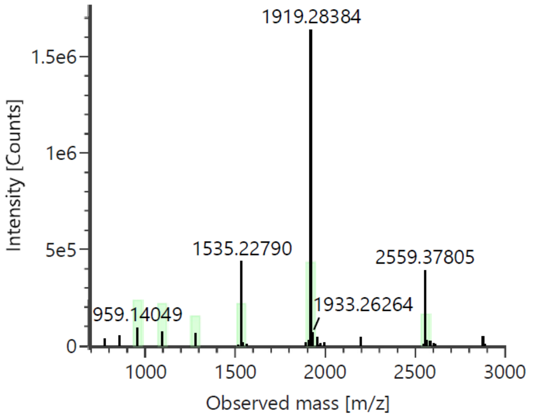

5’ - mU*fA*mA-fU-mA-fC-mU-fG-mC-fC-mG-fG-mG-fU-mA-fA-mU-fG-mA-fU-mG*fG*mA (**G5**)

| Item name | Observed RT (min) | Neutral mass (Da) | Observed neutral mass (Da) | Observed m/z | Mass error (mDa) | Mass error (ppm) |
|---|---|---|---|---|---|---|
| EN12762-33-006 | 4.73 | 7662.07 | 7662.0061 | 1914.4942 | -62.2 | -8.1 |

Channel name: TUV 260 : Integrated : Smoothed

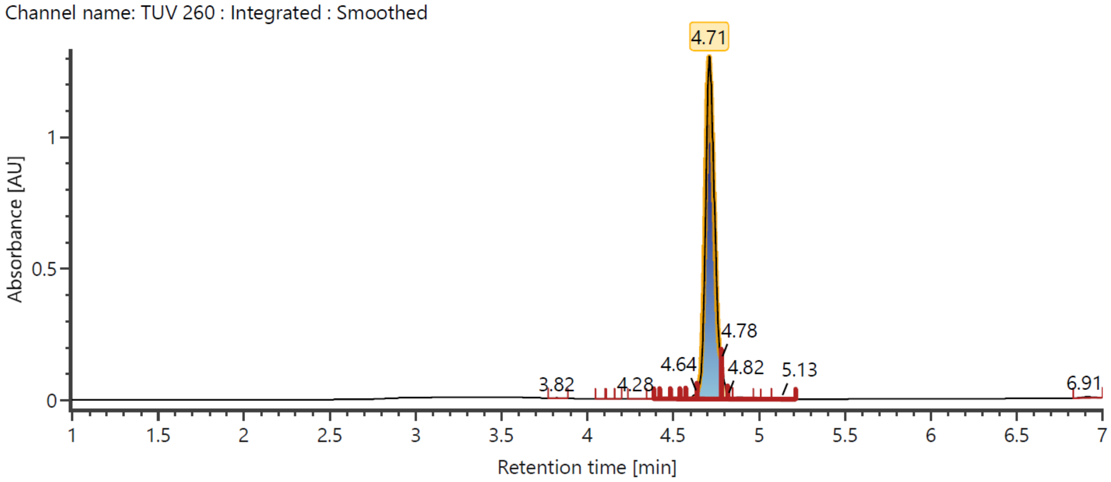


| . | Retention time (min) | Area | % Area (%) | Height |
|---|---|---|---|---|
| 1 | 4.71 | 4771328 | 94.37 | 1302491 |

**Item name: EN12762-33-006**
**Channel name: 1: TOF MS (400-5000) -40V ESI- (TIC)**

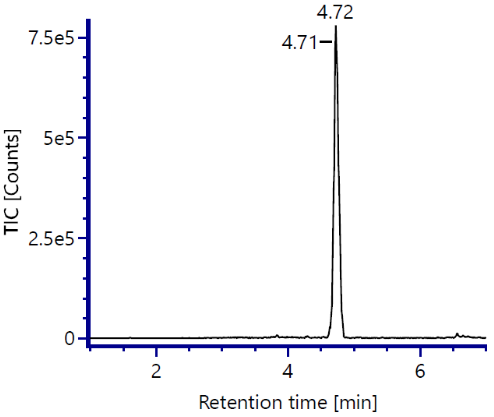


Item description: Channel name: Time 4.7283 +/- 0.0200 minutes

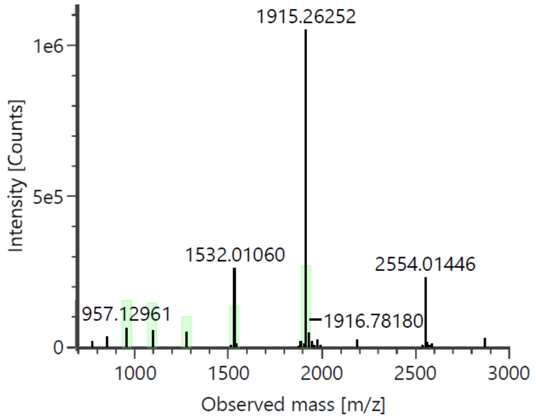

5’ - mU*fA*mA-fU-mA-fC-mU-fG-mC-fC-mG-fG-mG-fU-mA-fA-mU-fG-mA-fU-mG-fG-mA (**G6**)

| Item name | Observed RT (min) | Neutral mass (Da) | Observed neutral mass (Da) | Observed m/z | Mass error (mDa) | Mass error (ppm) |
|---|---|---|---|---|---|---|
| EN12762-33-005 | 4.69 | 7630.11 | 7630.0682 | 1906.5098 | -45.8 | -6.0 |

Channel name: TUV 260 : Integrated : Smoothed

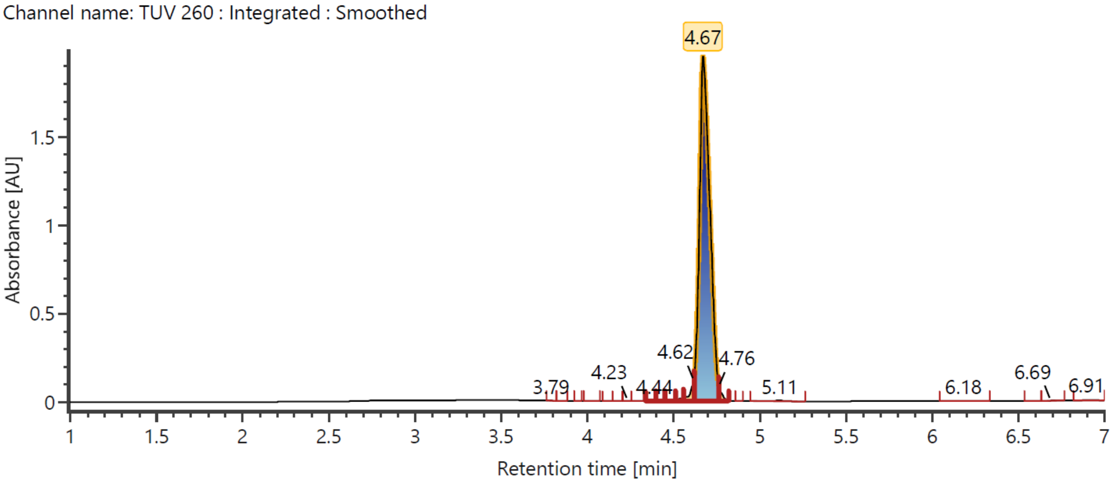


| . | Retention time (min) | Area | % Area (%) | Height |
|---|---|---|---|---|
| 1 | 4.67 | 8159454 | 96.01 | 1947997 |

**Item name: EN12762-33-005**
**Channel name: 1: TOF MS (400-5000) -40V ESI- (TIC)**

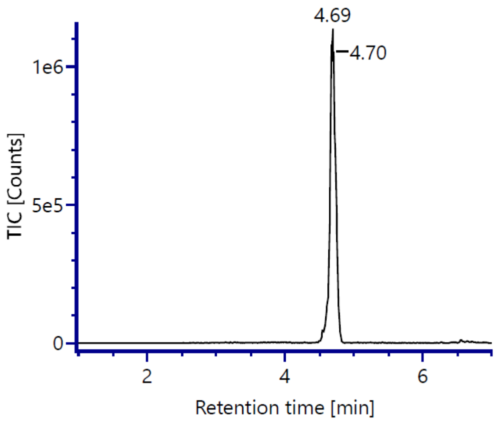


Item description: Channel name: Time 4.6939 +/- 0.0227 minutes

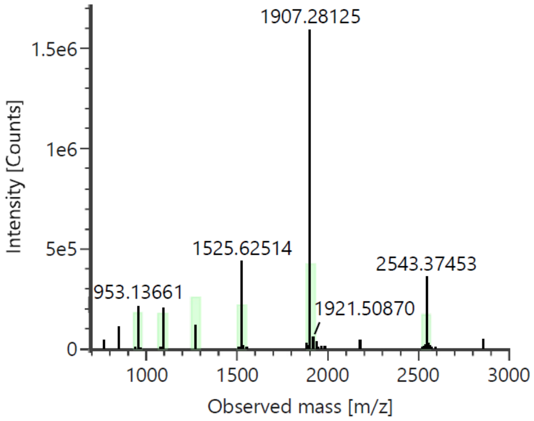

5’ - mU-fA-mA-fU-mA-fC-mU-fG-mC-fC-mG-fG-mG-fU-mA-fA-mU-fG-mA-fU-mG-fG-mA*mU*mU (**G7**)

| Item name | Observed RT (min) | Neutral mass (Da) | Observed neutral mass (Da) | Observed m/z | Mass error (mDa) | Mass error (ppm) |
|---|---|---|---|---|---|---|
| EN12762-33-007 | 4.76 | 8270.20 | 8270.1258 | 2066.5242 | -70.1 | -8.5 |

Channel name: TUV 260 : Integrated : Smoothed

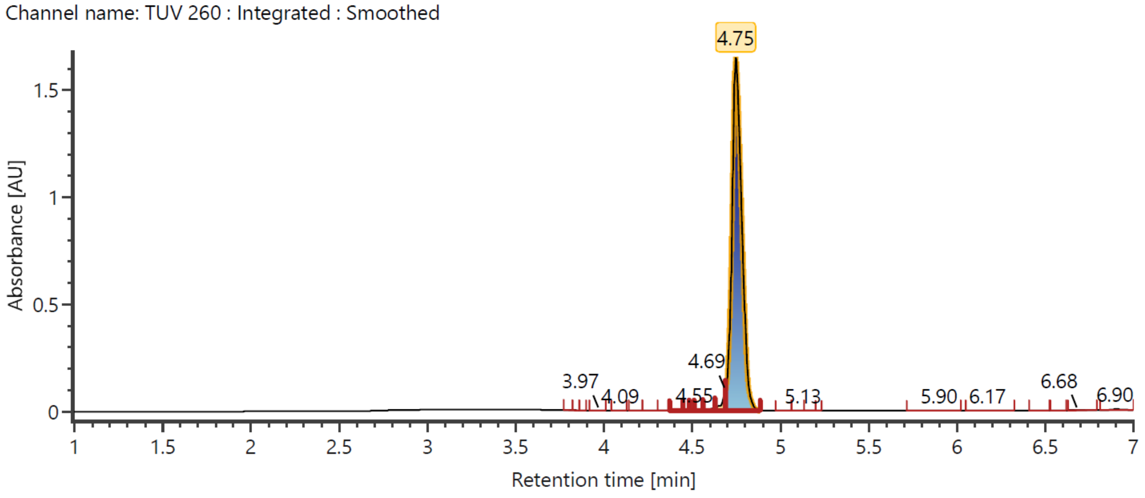


| . | Retention time (min) | Area | % Area (%) | Height |
|---|---|---|---|---|
| 1 | 4.75 | 6304812 | 96.76 | 1644901 |

**Item name: EN12762-33-007**
**Channel name: 1: TOF MS (400-5000) -40V ESI- (TIC)**

Item description: Channel name: Time 4.7601 +/- 0.0191 minutes

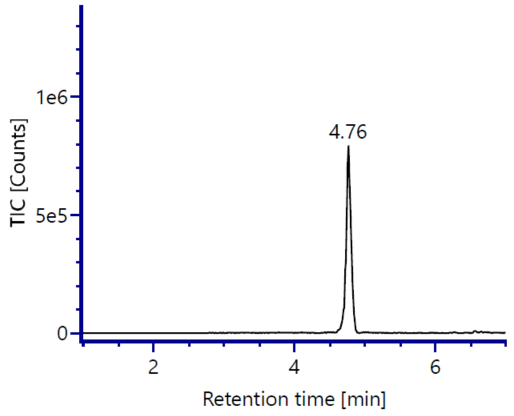


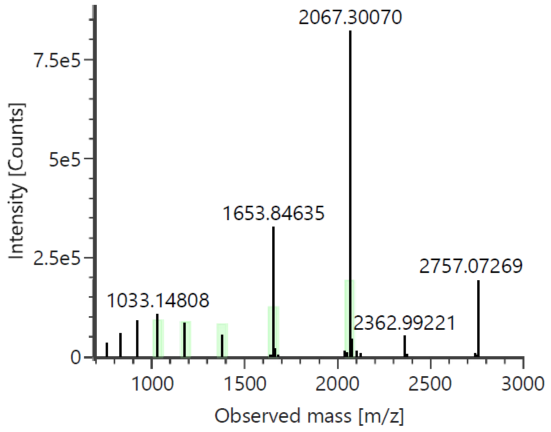

5’ - mU-fA-mA-fU-mA-fC-mU-fG-mC-fC-mG-mG-mG-fU-mA-fA-mU-fG-mA-fU-mG*fG*mA (**G8**)

| Item name | Observed RT (min) | Neutral mass (Da) | Observed neutral mass (Da) | Observed m/z | Mass error (mDa) | Mass error (ppm) |
|---|---|---|---|---|---|---|
| EN12762-37-001 | 4.77 | 7642.13 | 7642.0637 | 1909.5086 | -70.3 | -9.2 |

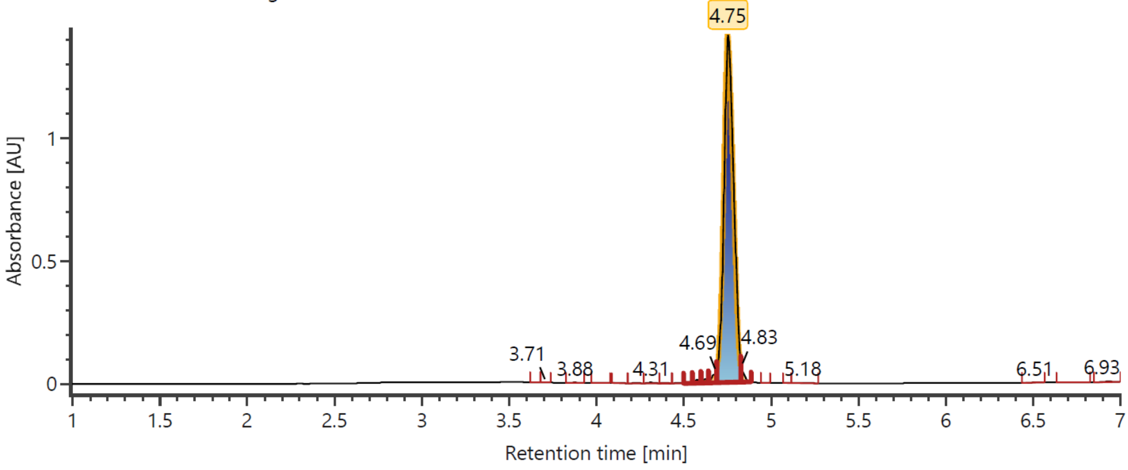


| . | Retention time (min) | Area | % Area (%) | Height |
|---|---|---|---|---|
| 1 | 4.75 | 5499467 | 96.02 | 1412404 |

**Item name: EN12762-37-001**
**Channel name: 1: TOF MS (400-5000) -40V ESI- (TIC)**

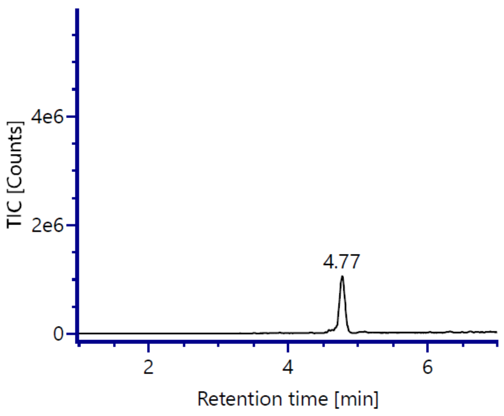


Item description: Channel name: Time 4.7665 +/- 0.0196 minutes

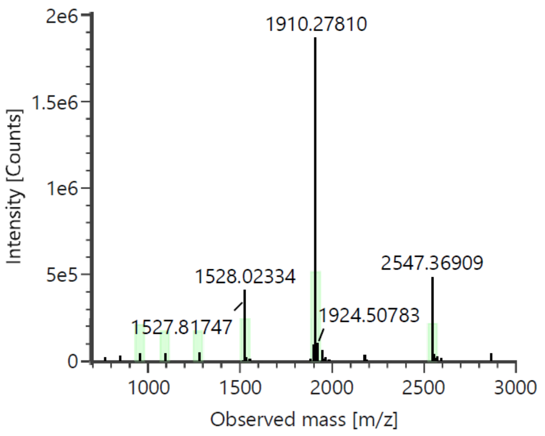

5’ - mU*fA*mA-fU-mA-fC-mU-fG-mC-fC-mG-mG-mG-fU-mA-fA-mU-fG-mA-fU-mG*mG*mA (**G9**)

| Item name | Observed RT (min) | Neutral mass (Da) | Observed neutral mass (Da) | Observed m/z | Mass error (mDa) | Mass error (ppm) |
|---|---|---|---|---|---|---|
| EN12762-37-002 | 4.78 | 7686.11 | 7686.0318 | 1920.5007 | -76.5 | -10.0 |

Channel name: TUV 260 : Integrated : Smoothed

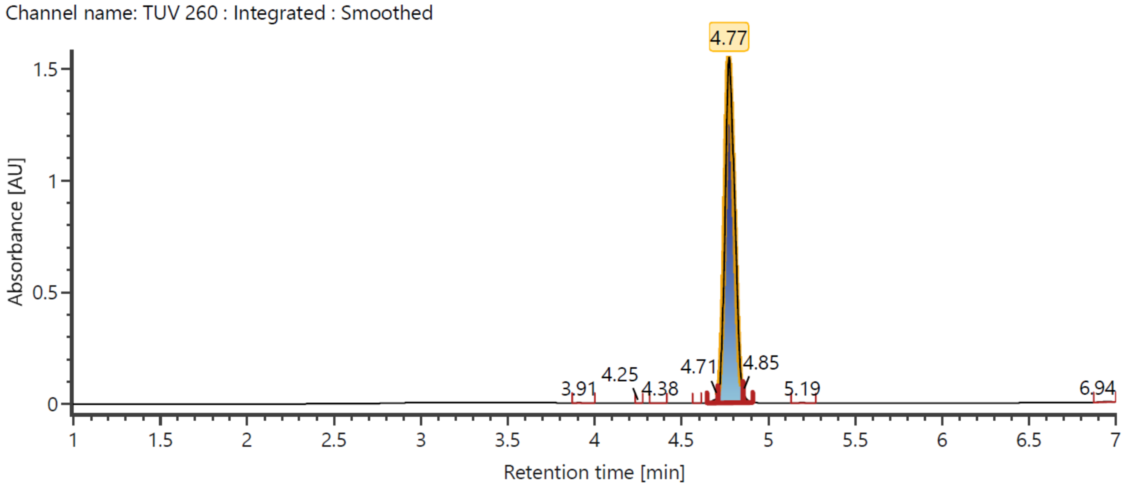


| . | Retention time (min) | Area | % Area (%) | Height |
|---|---|---|---|---|
| 1 | 4.77 | 5939329 | 98.14 | 1545802 |

**Item name: EN12762-37-002**
**Channel name: 1: TOF MS (400-5000) -40V ESI- (TIC)**

Item description:

Channel name: Time 4.7825 +/- 0.0184 minutes

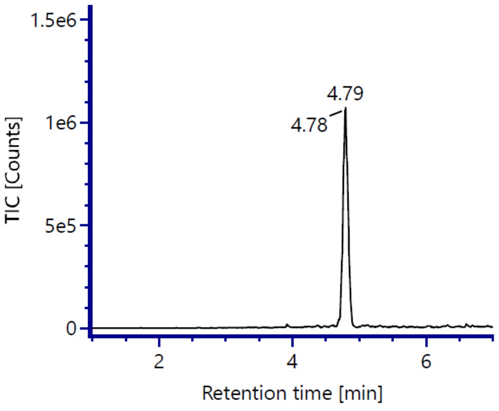


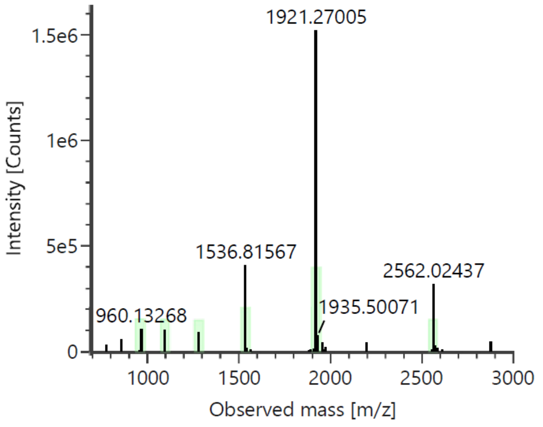

5’ - mU*fA*mA-mU-mA-fC-mU-fG-fC-mC-mG-mG-mG-fU-mA-fA-mU-mG-mA-mU-mG*mG*mA (**G10**)

| Item name | Observed RT (min) | Neutral mass (Da) | Observed neutral mass (Da) | Observed m/z | Mass error (mDa) | Mass error (ppm) |
|---|---|---|---|---|---|---|
| EN12762-37-3 | 4.76 | 7722.17 | 7722.0982 | 1929.5173 | -70.0 | -9.1 |

Channel name: TUV 260 : Integrated : Smoothed

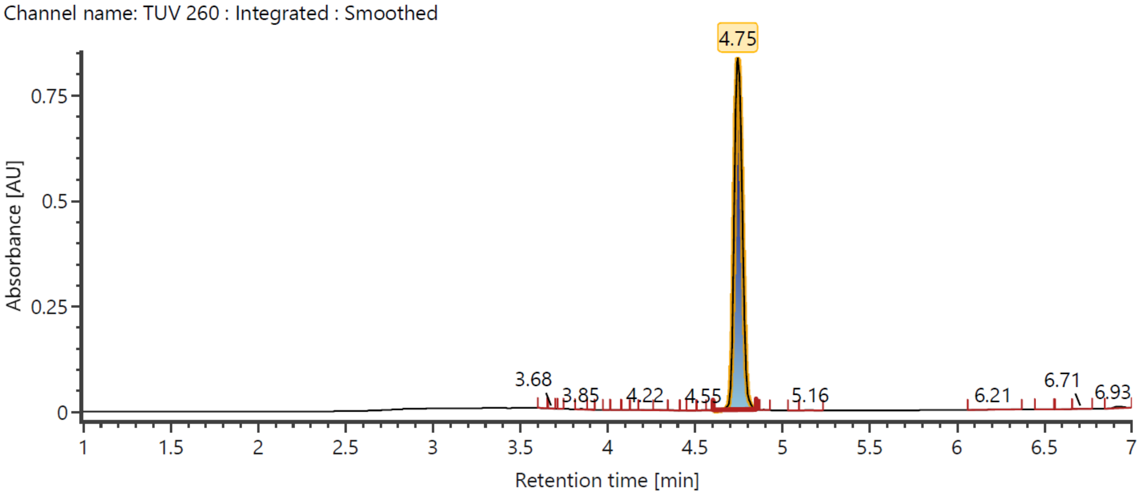


| . | Retention time (min) | Area | % Area (%) | Height |
|---|---|---|---|---|
| 1 | 4.75 | 2632987 | 98.59 | 833135 |

**Item name: EN12762-37-3**
**Channel name: 1: TOF MS (400-5000) -40V ESI- (TIC)**

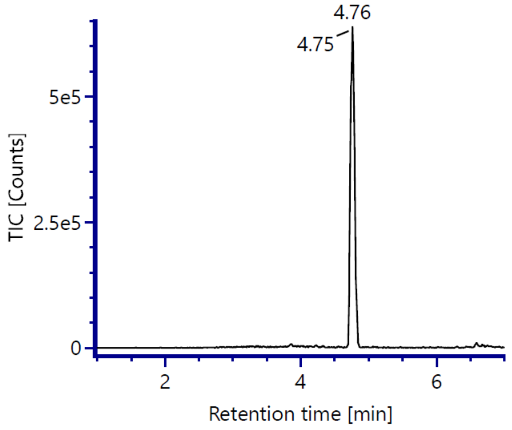


Item description: Channel name: Time 4.7603 +/- 0.0178 minutes

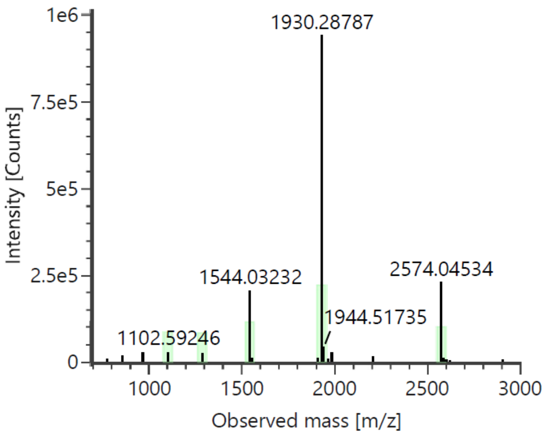

5’- mU*fA*mA-mU-mA-fC-mU-mG-mC-mC-mG-mG-mG-fU-mA-fA-mU-mG-mA-mU-mG*mG*mA (**G11**)

| Item name | Observed RT (min) | Neutral mass (Da) | Observed neutral mass (Da) | Observed m/z | Mass error (mDa) | Mass error (ppm) |
|---|---|---|---|---|---|---|
| EN12762-37-004 | 4.69 | 7746.21 | 7746.1352 | 1935.5265 | -73.0 | -9.4 |

Channel name: TUV 260 : Integrated : Smoothed

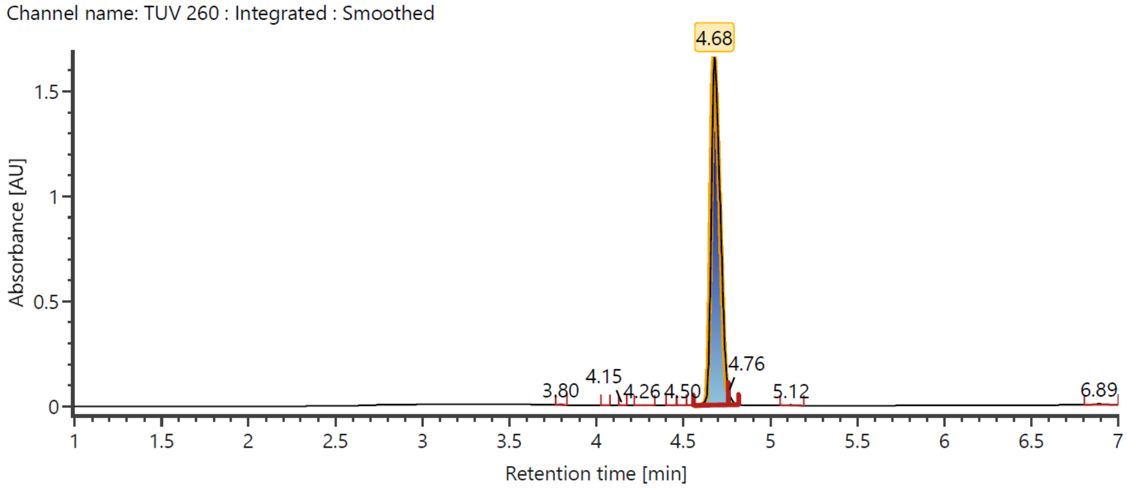


| . | Retention time (min) | Area | % Area (%) | Height |
|---|---|---|---|---|
| 1 | 4.68 | 5962873 | 98.17 | 1655935 |

**Item name: EN12762-37-004**
**Channel name: 1: TOF MS (400-5000) -40V ESI- (TIC)**

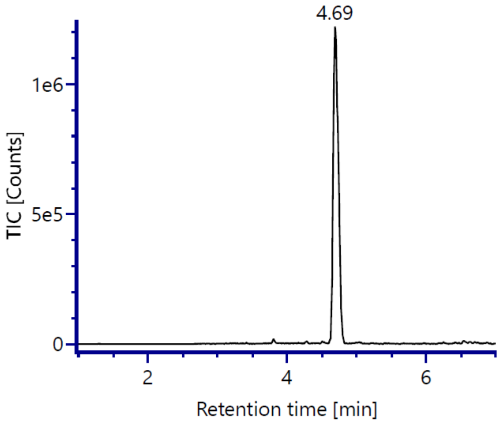


Item description: Channel name: Time 4.6908 +/- 0.0171 minutes

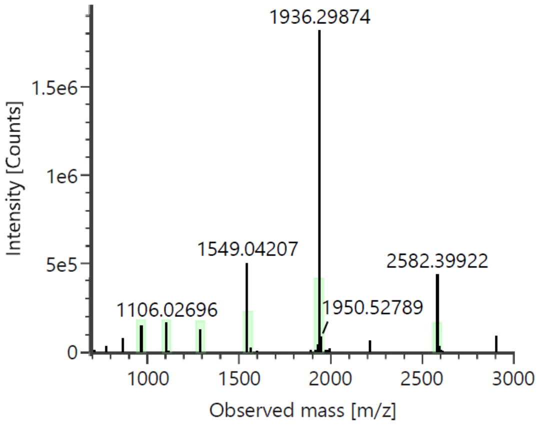

5’ - mU-fA-mA-fU-mA-fC-mU-fG-mC-fC-mG-fG-mG-fU-mA-fA-mU-fG-mA-fU-mG*fG*mA (**G12**)

| Item name | Observed RT (min) | Neutral mass (Da) | Observed neutral mass (Da) | Observed m/z | Mass error (mDa) | Mass error (ppm) |
|---|---|---|---|---|---|---|
| EN12762-38-001 | 4.67 | 7630.11 | 7630.0561 | 1906.5068 | -57.9 | -7.6 |

Channel name: TUV 260 : Integrated : Smoothed

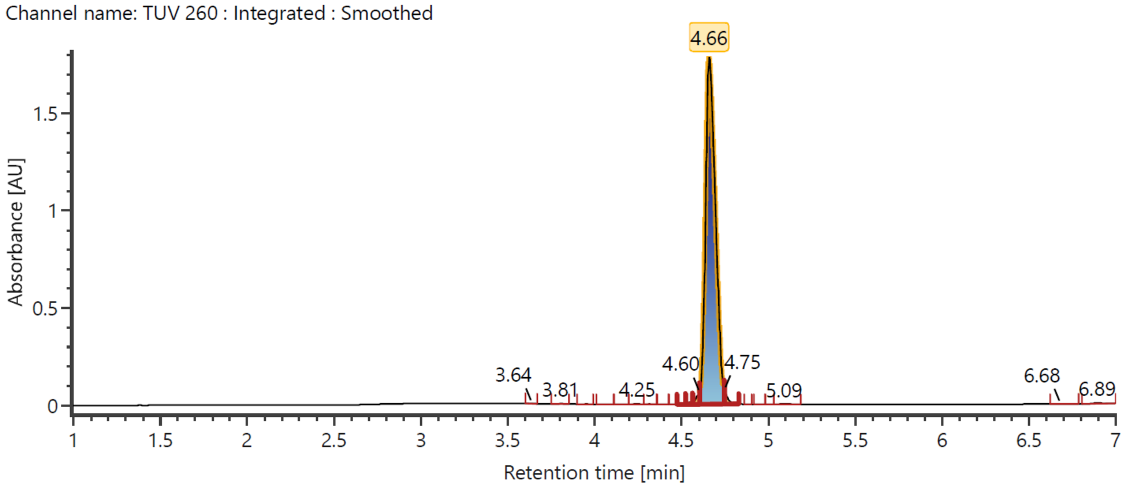


| . | Retention time (min) | Area | % Area (%) | Height |
|---|---|---|---|---|
| 1 | 4.66 | 6992661 | 96.45 | 1781308 |

**Item name: EN12762-38-001**
**Channel name: 1: TOF MS (400-5000) -40V ESI- (TIC)**

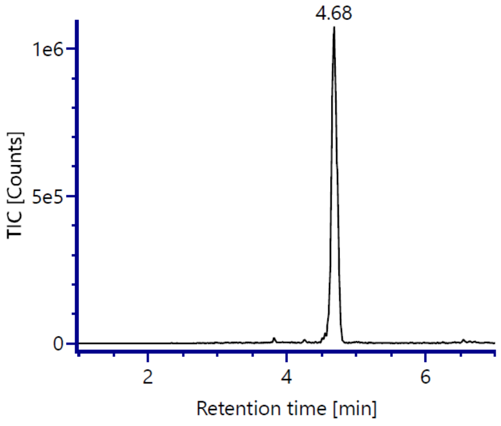


Item description: Channel name: Time 4.6740 +/- 0.0191 minutes

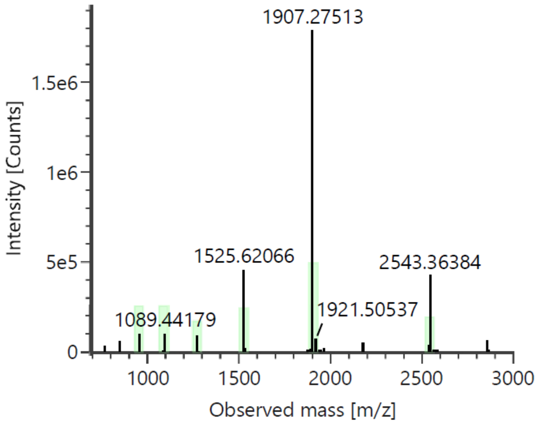

5'- fU-mA-fA-mU-fA-mC-fU-mG-fC-mC-fG-mG-fG-mU-fA-mA-fU-mG-fA-mU-fG*mG*fA (**G13**)

| Item name | Observed RT (min) | Neutral mass (Da) | Observed neutral mass (Da) | Observed m/z | Mass error (mDa) | Mass error (ppm) |
|---|---|---|---|---|---|---|
| EN12762-38-2 | 4.73 | 7618.09 | 7618.0245 | 1903.4988 | -69.5 | -9.1 |

Channel name: TUV 260 : Integrated : Smoothed

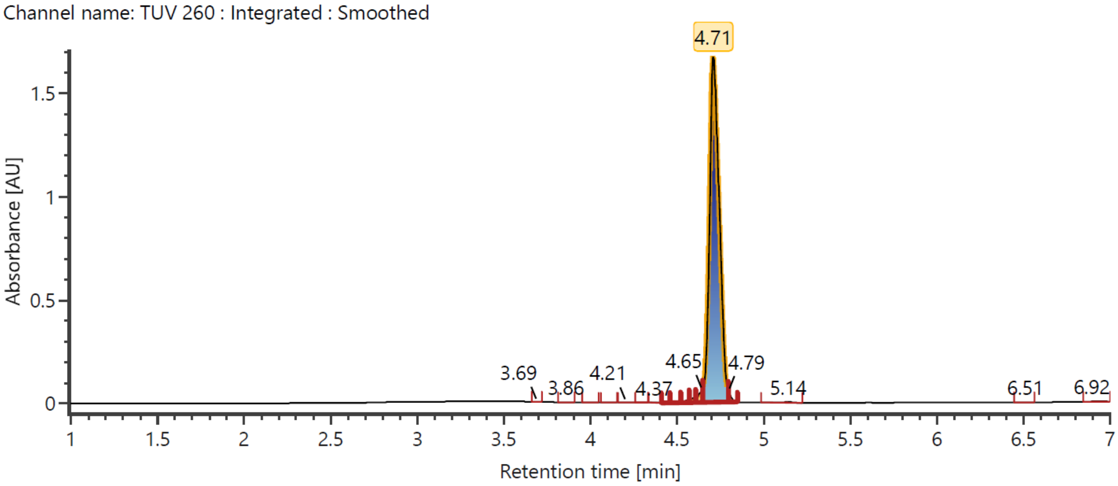


| . | Retention time (min) | Area | % Area (%) | Height |
|---|---|---|---|---|
| 1 | 4.71 | 6513541 | 96.53 | 1671454 |

**Item name: EN12762-38-2**
**Channel name: 1: TOF MS (400-5000) -40V ESI- (TIC)**

Item description: Channel name: Time 4.7250 +/- 0.0175 minutes

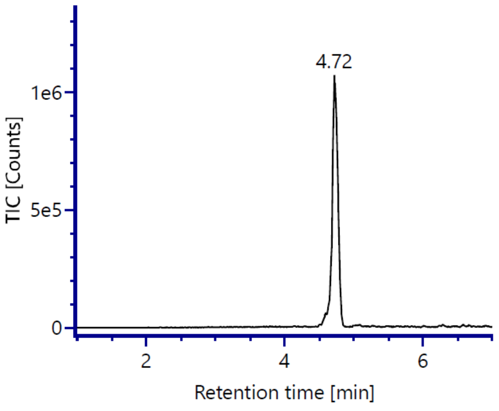


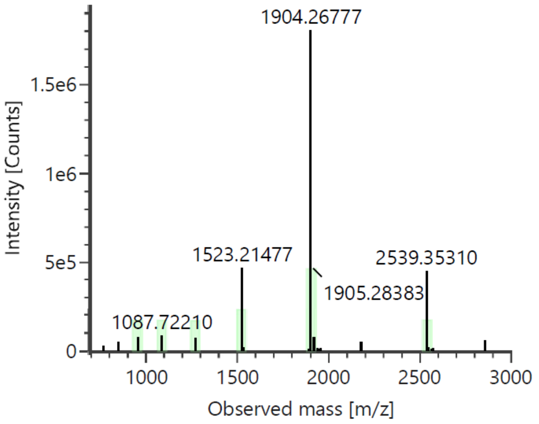

5'- fU-rA-rA-fU-rA-fC-fU-rG-fC-fC-rG-rG-rG-fU-rA-rA-fU-rG-rA-fU-rG*rG*rA (**G14**)

| Item name | Observed RT (min) | Neutral mass (Da) | Observed neutral mass (Da) | Observed m/z | Mass error (mDa) | Mass error (ppm) |
|---|---|---|---|---|---|---|
| EN12762-38-003 | 4.71 | 7457.93 | 7457.8790 | 1863.4625 | -55.9 | -7.5 |

Channel name: TUV 260 : Integrated : Smoothed

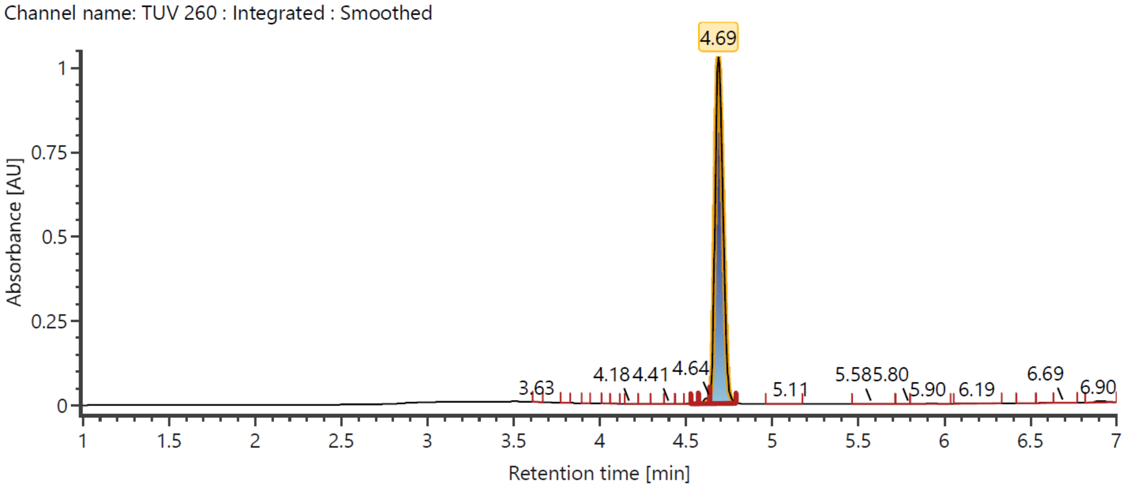


| . | Retention time (min) | Area | % Area (%) | Height |
|---|---|---|---|---|
| 1 | 4.69 | 3303349 | 97.72 | 1025959 |

**Item name: EN12762-38-003**
**Channel name: 1: TOF MS (400-5000) -40V ESI- (TIC)**

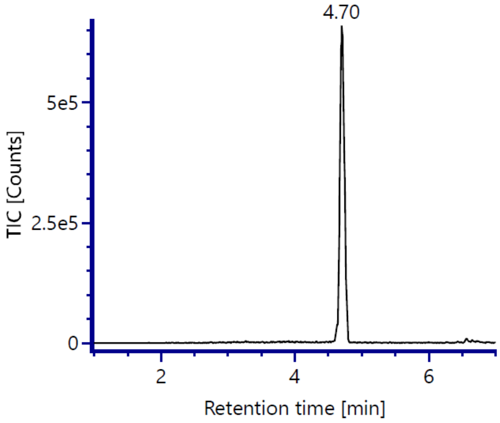


Item description: Channel name: Time 4.7050 +/- 0.0161 minutes

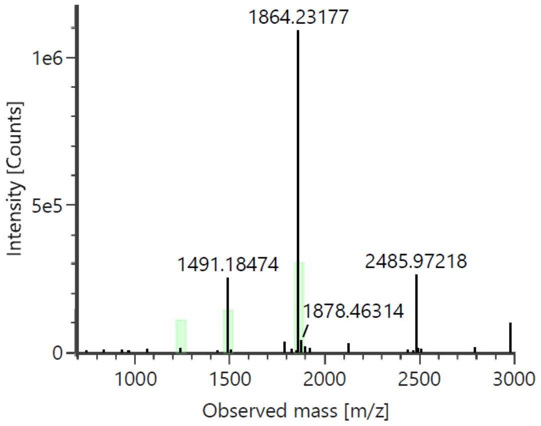

5’- rU-fA-fA-rU-fA-rC-rU-fG-rC-rC-fG-fG-fG-rU-fA-fA-rU-fG-fA-rU-fG*fG*fA (**G15**)

| Item name | Observed RT (min) | Neutral mass (Da) | Observed neutral mass (Da) | Observed m/z | Mass error (mDa) | Mass error (ppm) |
|---|---|---|---|---|---|---|
| EN12762-69-1 | 4.73 | 7467.91 | 7467.8573 | 1865.9570 | -55.9 | -7.5 |

Channel name: TUV 260 : Integrated : Smoothed

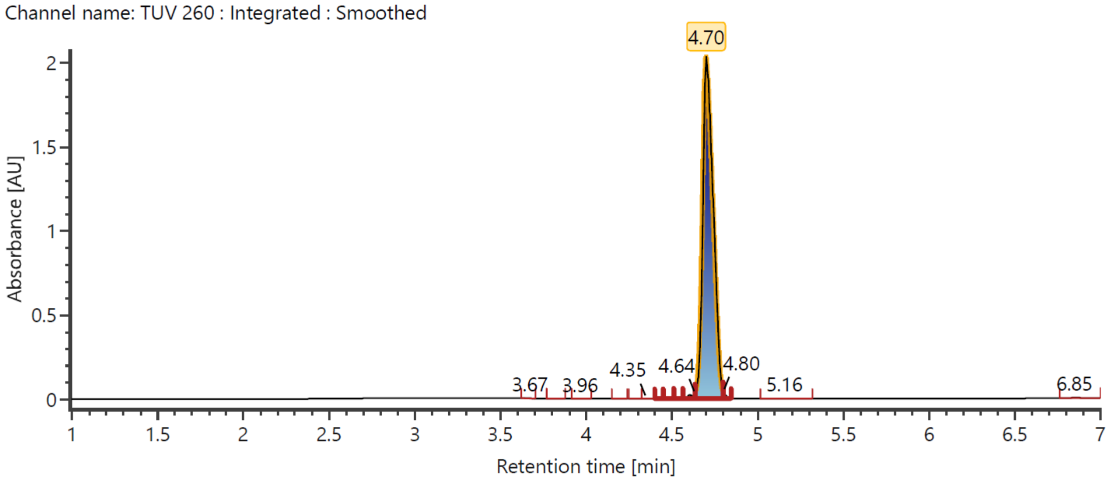


| . | Retention time (min) | Area | % Area (%) | Height |
|---|---|---|---|---|
| 1 | 4.70 | 8591479 | 98.43 | 2033326 |

**Item name: EN12762-69-1**
**Channel name: 1: TOF MS (400-5000) -40V ESI- (TIC)**

Item description:

Channel name: Time 4.7326 +/- 0.0188 minutes

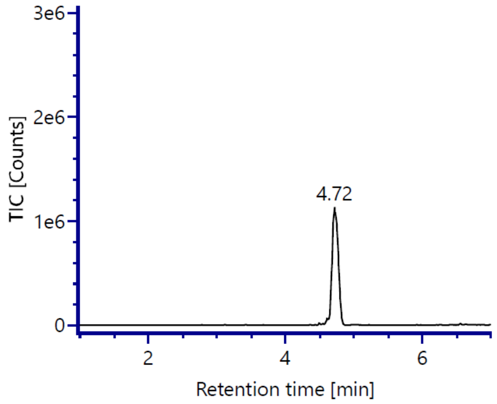


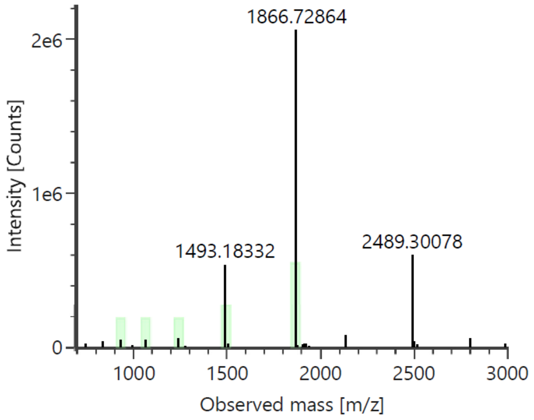

5’- mU-rA-rA-mU-rA-mC-mU-rG-mC-mC-rG-rG-rG-mU-rA-rA-mU-rG-rA-mU-rG*rG*rA (**G16**)

| Item name | Observed RT (min) | Neutral mass (Da) | Observed neutral mass (Da) | Observed m/z | Mass error (mDa) | Mass error (ppm) |
|---|---|---|---|---|---|---|
| EN12762-69-2 | 4.75 | 7566.11 | 7566.0646 | 1890.5089 | -50.1 | -6.6 |

Channel name: TUV 260 : Integrated : Smoothed

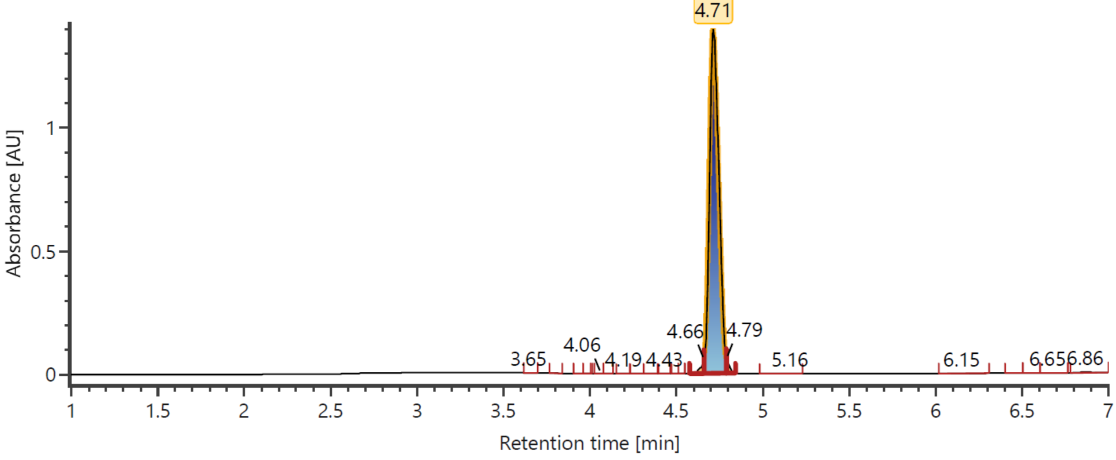


| . | Retention time (min) | Area | % Area (%) | Height |
|---|---|---|---|---|
| 1 | 4.71 | 5224510 | 97.06 | 1393921 |

**Item name: EN12762-69-2**
**Channel name: 1: TOF MS (400-5000) -40V ESI- (TIC)**

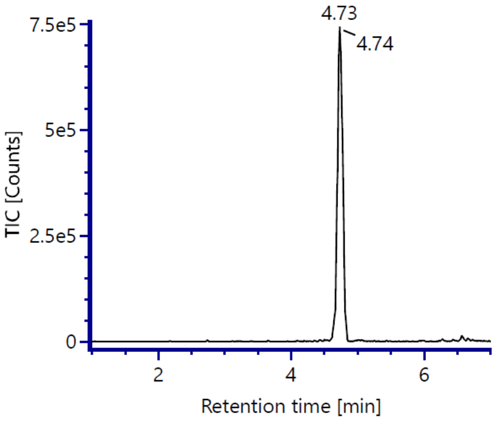


Item description: Channel name: Time 4.7474 +/- 0.0163 minutes

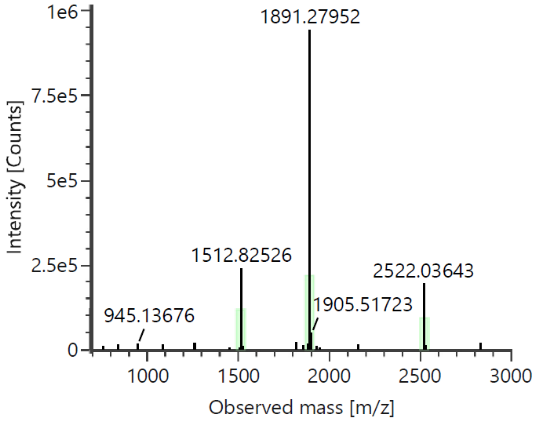

5'- fU*rA*rA-fU-rA-fC-fU-rG-fC-fC-rG-rG-rG-fU-rA-rA-fU-rG-rA-fU-rG*rG*rA (**G17**)

| Item name | Observed RT (min) | Neutral mass (Da) | Observed neutral mass (Da) | Observed m/z | Mass error (mDa) | Mass error (ppm) |
|---|---|---|---|---|---|---|
| EN12762-69-3 | 4.71 | 7457.93 | 7457.8449 | 1863.4540 | -89.9 | -12.1 |

Channel name: TUV 260 : Integrated : Smoothed

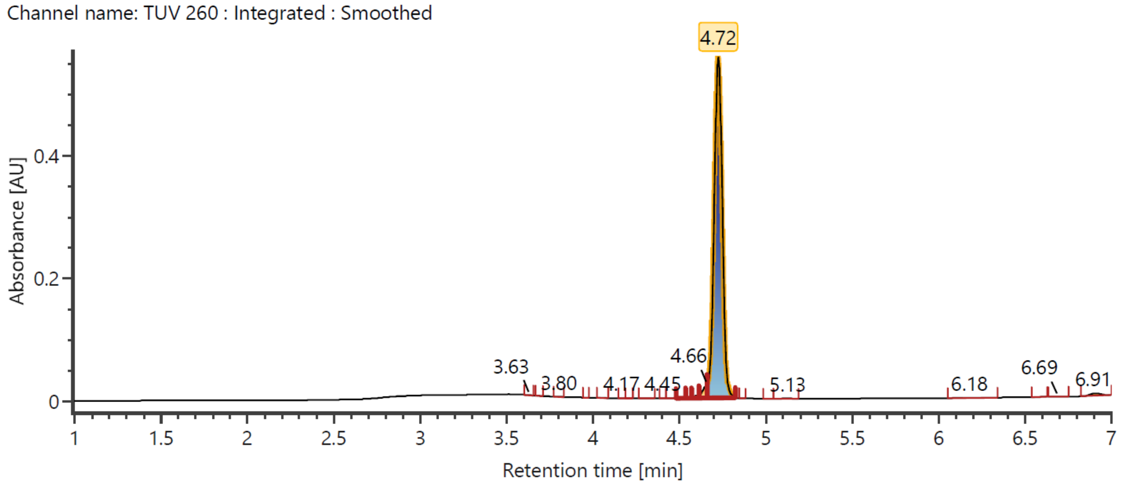


| . | Retention time (min) | Area | % Area (%) | Height |
|---|---|---|---|---|
| 1 | 4.72 | 1812755 | 95.97 | 556014 |

**Item name: EN12762-69-3**
**Channel name: 1: TOF MS (400-5000) -40V ESI- (TIC)**

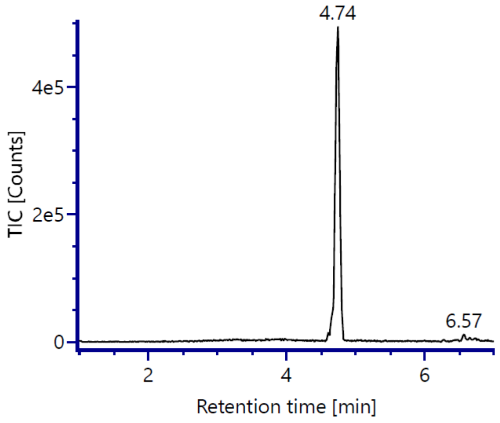


Item description: Channel name: Time 4.7111 +/- 0.0182 minutes

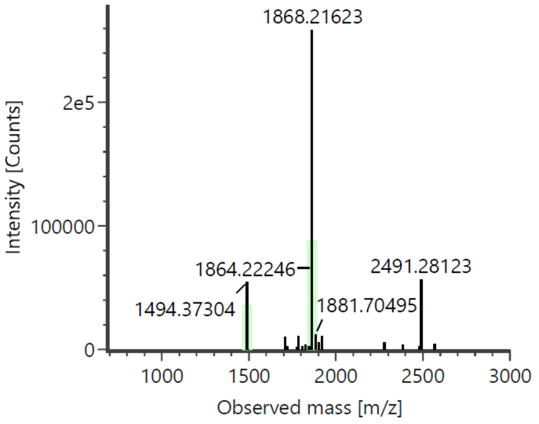

5'- rU-rU-rA-rA-rU-rG-rC-rU-rA-rA-rU-rC-rG-rU-rG-rA-rU-rA-rG-rG-rG-rG-rU-rU (**GA1**)

| Item name | Observed RT (min) | Neutral mass (Da) | Observed neutral mass (Da) | Observed m/z | Mass error (mDa) | Mass error (ppm) |
|---|---|---|---|---|---|---|
| EN12762-97-001 | 4.75 | 7692.00 | 7691.9098 | 1921.9702 | -91.9 | -12.0 |

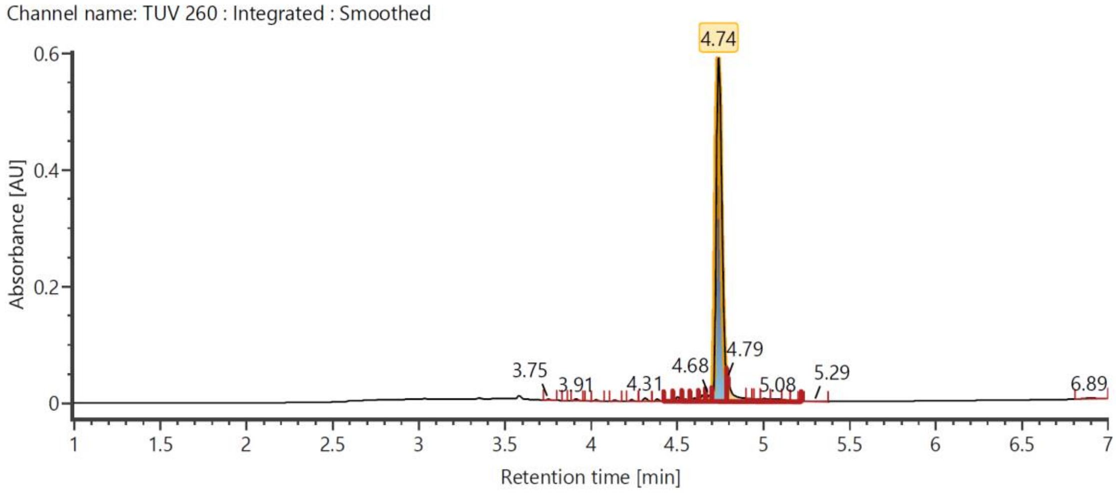


| . | Retention time (min) | Area | % Area (%) | Height |
|---|---|---|---|---|
| 1 | 4.74 | 1360037 | 84.23 | 589307 |

**Item name: EN12762-97-001**
**Channel name: 1: TOF MS (400-5000) -40V ESI- (TIC)**

Item description: Channel name: Time 4.7538 +/- 0.0172 minutes

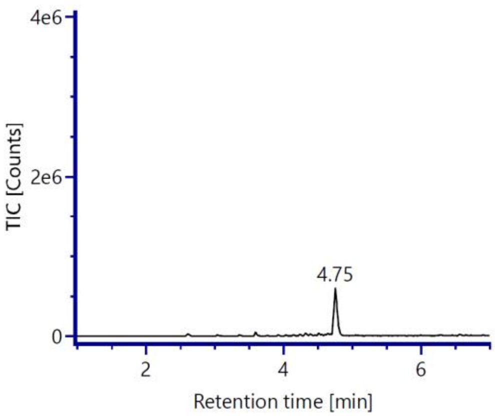


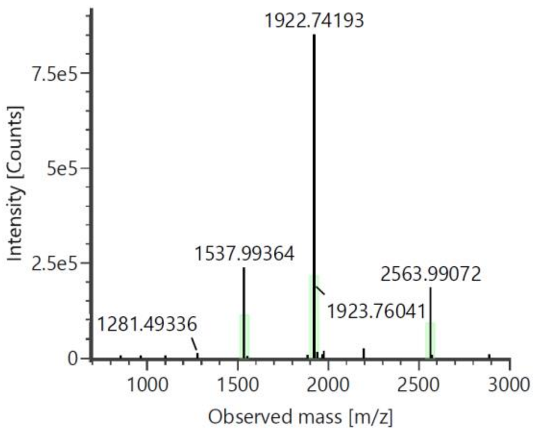

5’- fU-fU-rA-rA-fU-rG-fC-fU-rA-rA-fU-fC-rG-fU-rG-rA-fU-rA-rG-rG-rG-rG*fU*fU (**GA2**)

| Item name | Observed RT (min) | Neutral mass (Da) | Observed neutral mass (Da) | Observed m/z | Mass error (mDa) | Mass error (ppm) |
|---|---|---|---|---|---|---|
| EN12762-97-002 | 4.80 | 7745.91 | 7745.8270 | 1935.4495 | -81.3 | -10.5 |

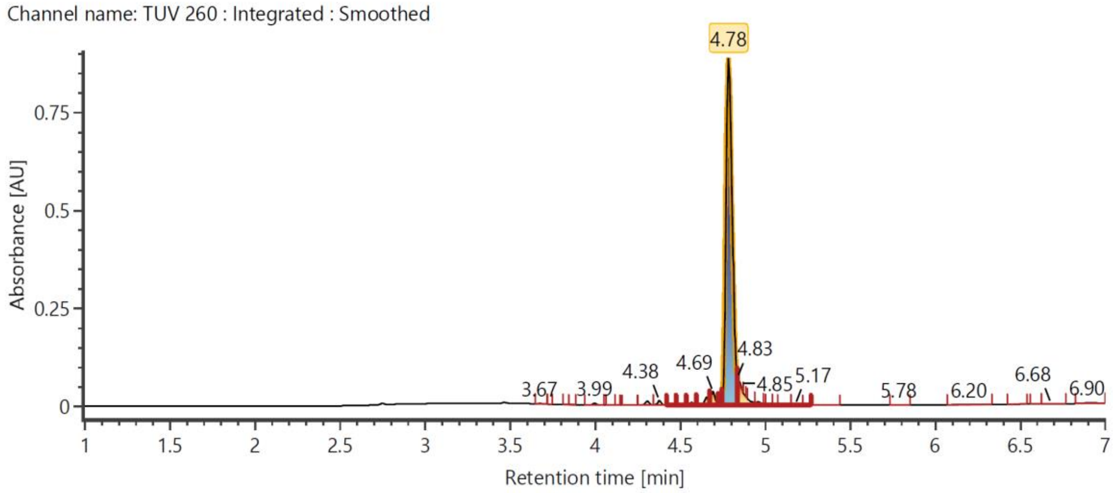


| . | Retention time (min) | Area | % Area (%) | Height |
|---|---|---|---|---|
| 1 | 4.78 | 2303167 | 85.51 | 885619 |

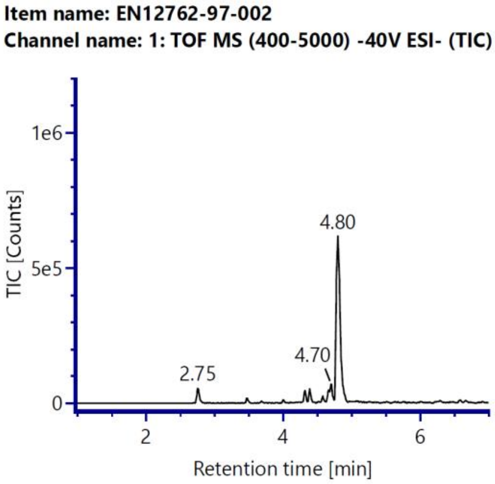


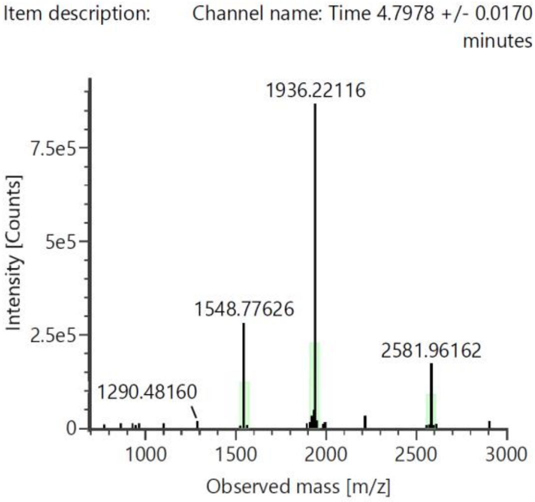

5'- mU*fU*mA-mA-mU-fG-mC-fU-fA-mA-mU-mC-mG-fU-mG-fA-mU-mA-mG-mG-mG-mG*mU*mU (**GA3**)

| Item name | Observed RT (min) | Neutral mass (Da) | Observed neutral mass (Da) | Observed m/z | Mass error (mDa) | Mass error (ppm) |
|---|---|---|---|---|---|---|
| EN12762-97-003 | 4.80 | 8020.17 | 8020.0784 | 2004.0123 | -87.6 | -10.9 |

Channel name: TUV 260 : Integrated : Smoothed

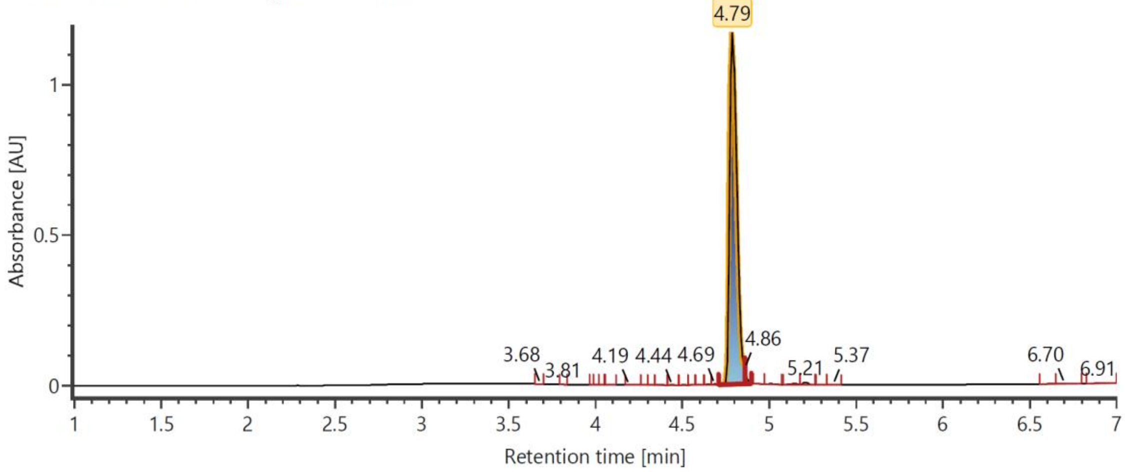


| . | Retention time (min) | Area | % Area (%) | Height |
|---|---|---|---|---|
| 1 | 4.79 | 3367178 | 97.49 | 1166167 |

**Item name: EN12762-97-003**
**Channel name: 1: TOF MS (400-5000) -40V ESI- (TIC)**

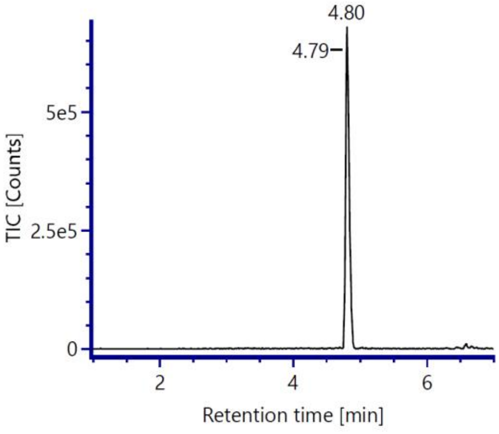


Item description: Channel name: Time 4.8013 +/- 0.0171 minutes

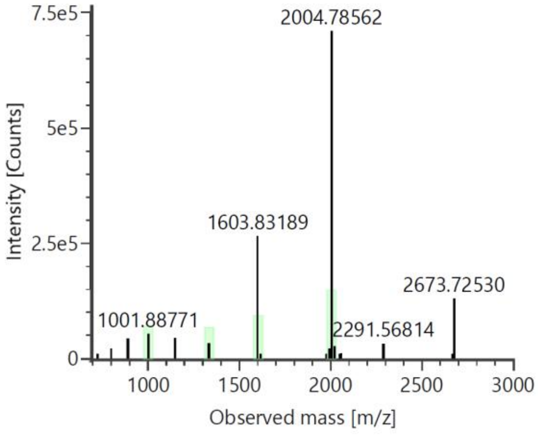

5’- rU-rG-rG-rC-rA-rG-rU-rG-rU-rC-rU-rU-rA-rG-rC-rU-rG-rG-rU-rU-rG-rU (**GB1**)

| Item name | Observed RT (min) | Neutral mass (Da) | Observed neutral mass (Da) | Observed m/z | Mass error (mDa) | Mass error (ppm) |
|---|---|---|---|---|---|---|
| EN12762-95-001 | 4.68 | 7025.88 | 7025.8569 | 1755.4570 | -23.4 | -3.3 |

Channel name: TUV 260 : Integrated : Smoothed

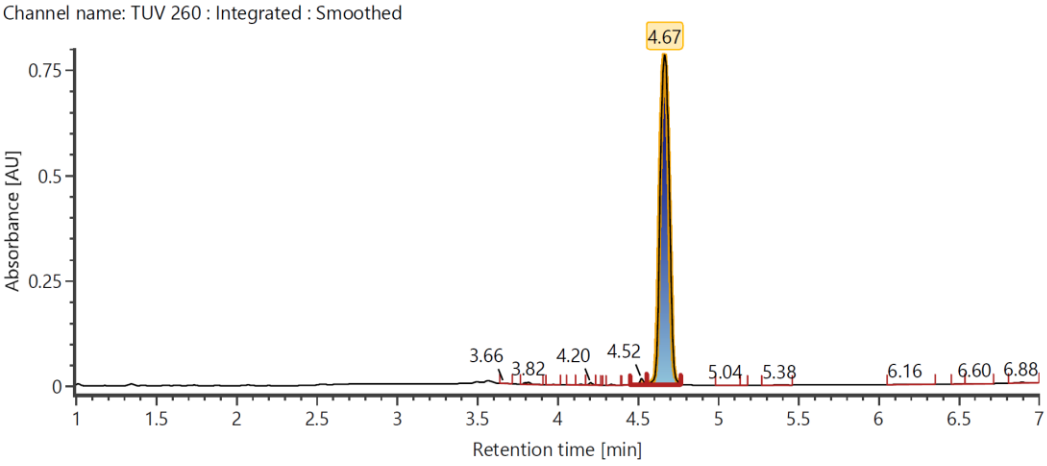


| . | Retention time (min) | Area | % Area (%) | Height |
|---|---|---|---|---|
| 1 | 4.67 | 3078137 | 97.59 | 782375 |

**Item name: EN12762-95-001**
**Channel name: 1: TOF MS (400-5000) -40V ESI- (TIC)**

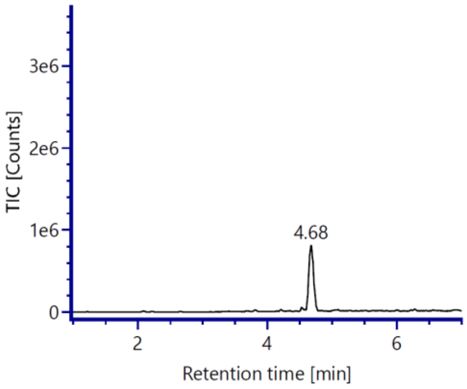


Item description: Channel name: Time 4.6765 +/- 0.0185 minutes

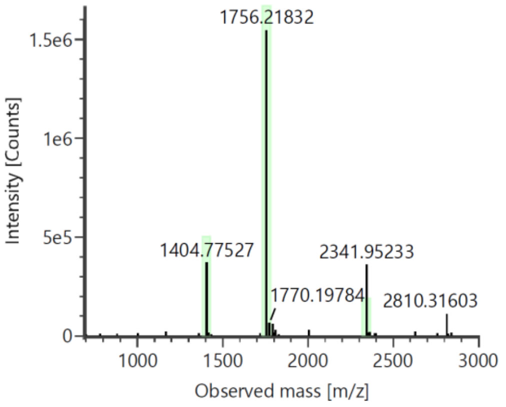

5'- fU-rG-rG-fC-rA-rG-fU-rG-fU-fC-fU-fU-rA-rG-fC-fU-rG-rG-fU-fU*rG*fU (**GB2**)

| Item name | Observed RT (min) | Neutral mass (Da) | Observed neutral mass (Da) | Observed m/z | Mass error (mDa) | Mass error (ppm) |
|---|---|---|---|---|---|---|
| EN12762-95-002 | 4.66 | 7081.78 | 7081.7414 | 1769.4281 | -41.2 | -5.8 |

Channel name: TUV 260 : Integrated : Smoothed

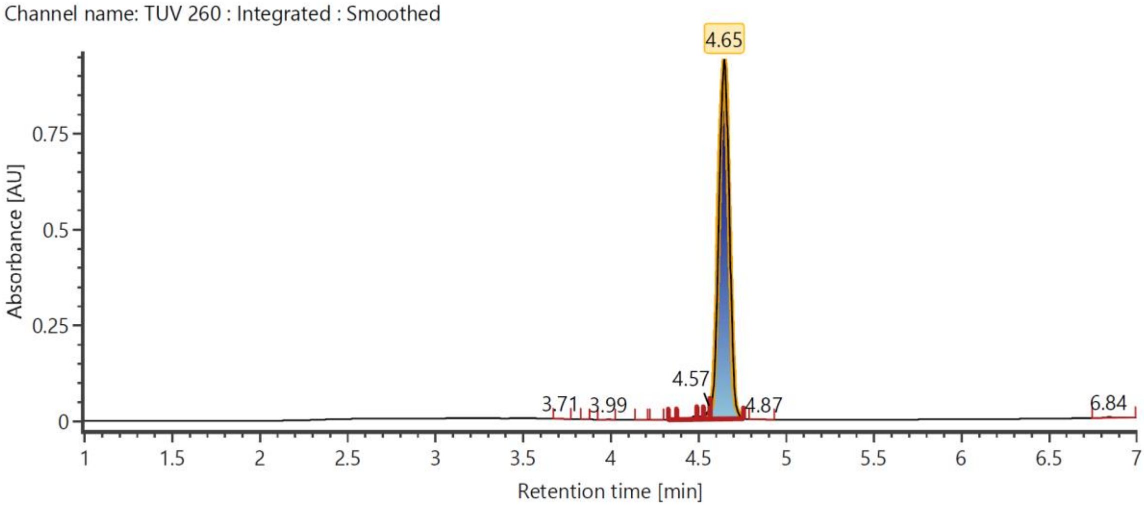


| . | Retention time (min) | Area | % Area (%) | Height |
|---|---|---|---|---|
| 1 | 4.65 | 3915687 | 97.92 | 937090 |

**Item name: EN12762-95-002**
**Channel name: 1: TOF MS (400-5000) -40V ESI- (TIC)**

Item description:

Channel name: Time 4.6618 +/- 0.0170 minutes

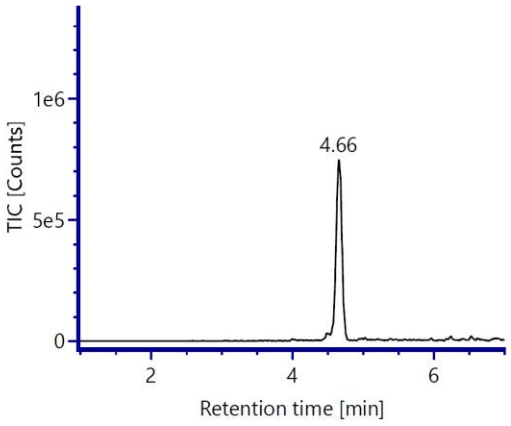


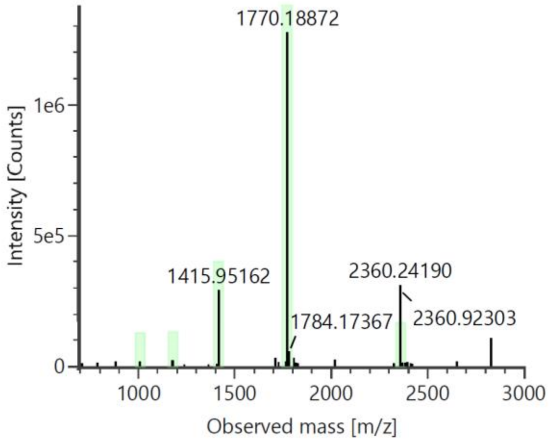

5’- mU*fG*mG-mC-mA-fG-mU-fG-fU-mC-mU-mU-fA-mG-fC-mU-mG-mG-mU-mU*mG*mU (**GB3**)

| Item name | Observed RT (min) | Neutral mass (Da) | Observed neutral mass (Da) | Observed m/z | Mass error (mDa) | Mass error (ppm) |
|---|---|---|---|---|---|---|
| EN12762-95-003 | 4.67 | 7326.01 | 7325.9555 | 1830.4816 | -57.8 | -7.9 |

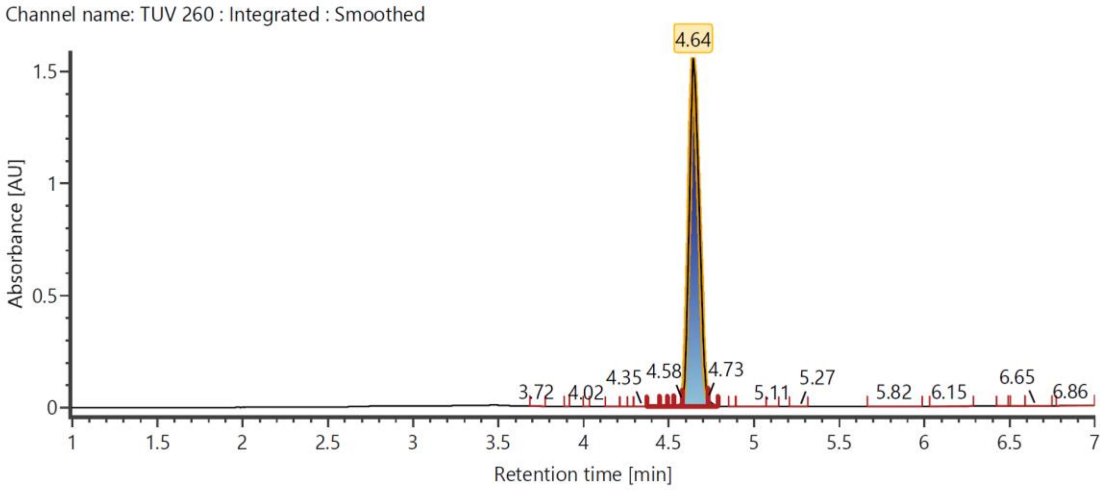


| . | Retention time (min) | Area | % Area (%) | Height |
|---|---|---|---|---|
| 1 | 4.64 | 6269941 | 98.47 | 1552225 |

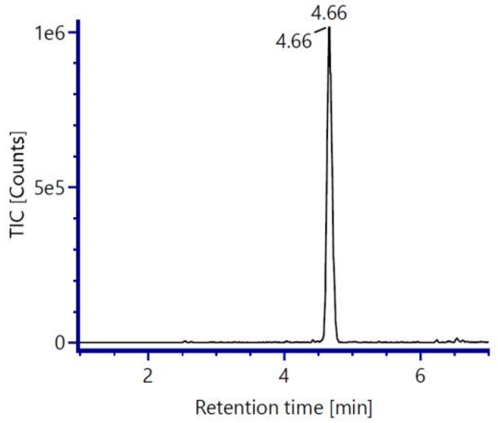


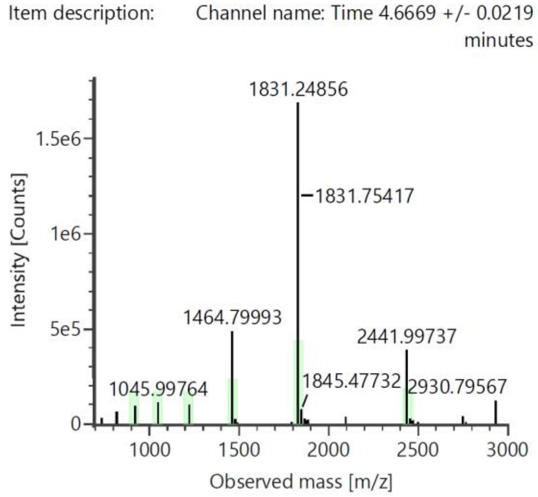

*Passenger strands*

5'- rC-rU-rC-rG-rU-rC-rA-rU-rU-rA-rC-rC-rC-rA-rG-rC-rA-rG-rU-rG-rU-rU-rU (**P1**)

| Item name | Observed RT (min) | Neutral mass (Da) | Observed neutral mass (Da) | Observed m/z | Mass error (mDa) | Mass error (ppm) |
|---|---|---|---|---|---|---|
| EN12762-35-001 | 4.70 | 7217.94 | 7217.8912 | 1803.4655 | -44.3 | -6.1 |

Channel name: TUV 260 : Integrated : Smoothed

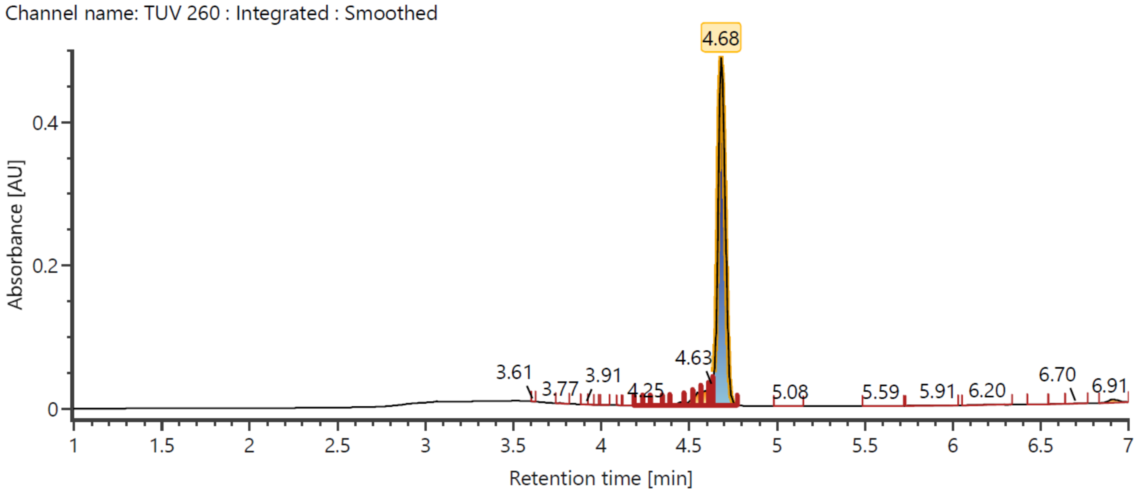


| . | Retention time (min) | Area | % Area (%) | Height |
|---|---|---|---|---|
| 1 | 4.68 | 1423279 | 88.80 | 484980 |

**Item name: EN12762-35-001**
**Channel name: 1: TOF MS (400-5000) -40V ESI- (TIC)**

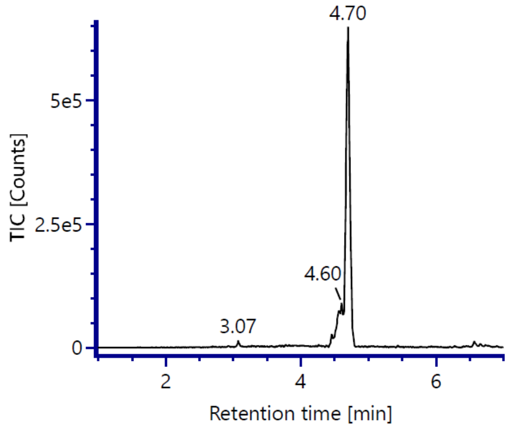


Item description: Channel name: Time 4.6967 +/- 0.0168 minutes

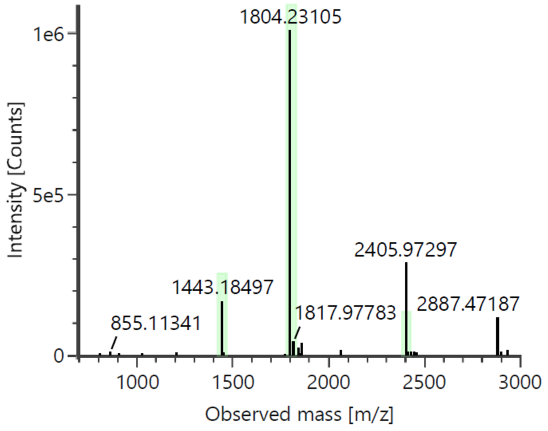

5'- fC-mU-fC-mG-fU-mC-fA-mU-fU-mA-fC-mC-fC-mA-fG-mC-fA-mG-fU-mG-fU-mU-fU (**P2**)

| Item name | Observed RT (min) | Neutral mass (Da) | Observed neutral mass (Da) | Observed m/z | Mass error (mDa) | Mass error (ppm) |
|---|---|---|---|---|---|---|
| EN12762-35-002 | 4.70 | 7396.06 | 7396.0514 | 1478.2030 | -4.2 | -0.6 |

Channel name: TUV 260 : Integrated : Smoothed

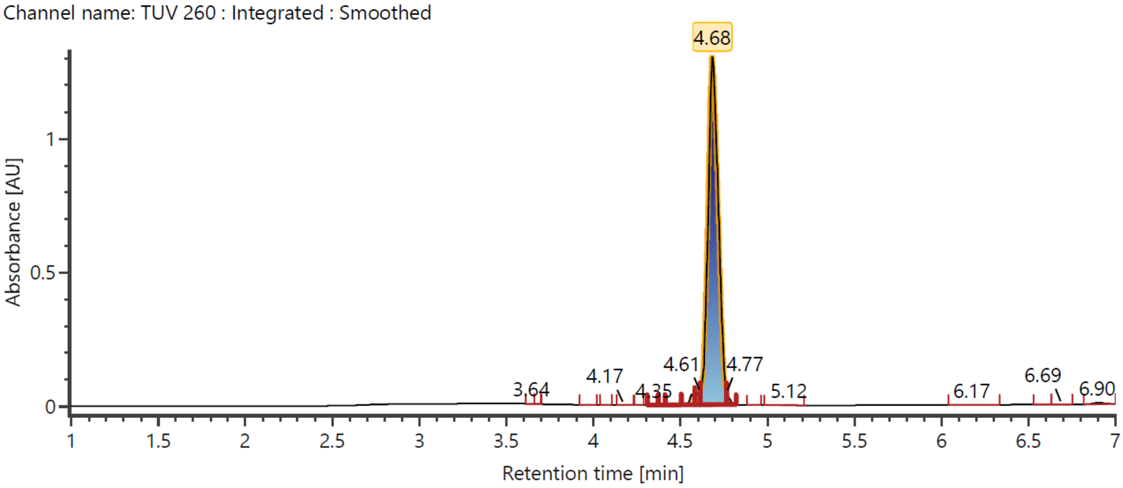


| . | Retention time (min) | Area | % Area (%) | Height |
|---|---|---|---|---|
| 1 | 4.68 | 5351360 | 95.61 | 1299225 |

**Item name: EN12762-35-002**
**Channel name: 1: TOF MS (400-5000) -40V ESI- (TIC)**

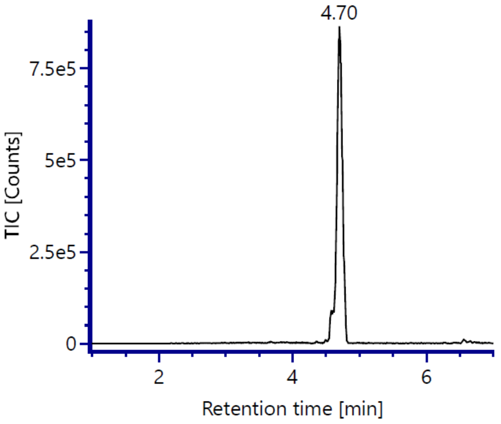


Item description: Channel name: Time 4.7034 +/- 0.0187 minutes

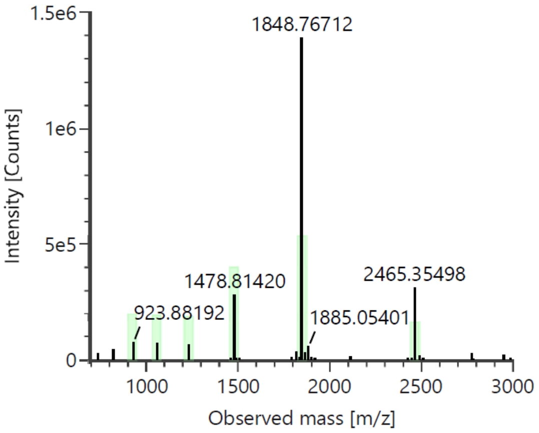

5'- mC-fU-mC-fG-mU-fC-mA-fU-mU-fA-mC-fC-mC-fA-mG-fC-mA-fG-mU-fG-mU-fU-mU (**P3**)

| Item name | Observed RT (min) | Neutral mass (Da) | Observed neutral mass (Da) | Observed m/z | Mass error (mDa) | Mass error (ppm) |
|---|---|---|---|---|---|---|
| EN12762-35-003 | 4.69 | 7408.08 | 7408.0340 | 1851.0012 | -41.6 | -5.6 |

Channel name: TUV 260 : Integrated : Smoothed

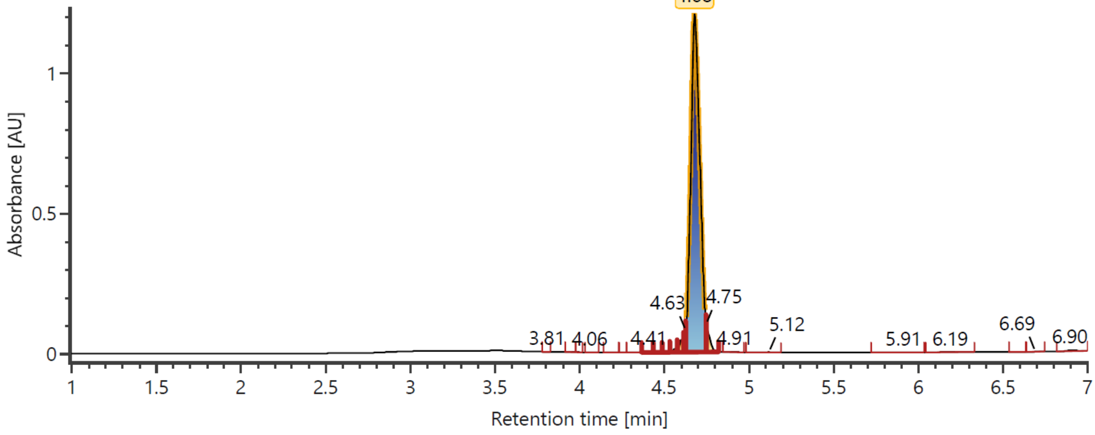


| . | Retention time (min) | Area | % Area (%) | Height |
|---|---|---|---|---|
| 1 | 4.68 | 4498313 | 93.47 | 1205723 |

**Item name: EN12762-35-003**
**Channel name: 1: TOF MS (400-5000) -40V ESI- (TIC)**

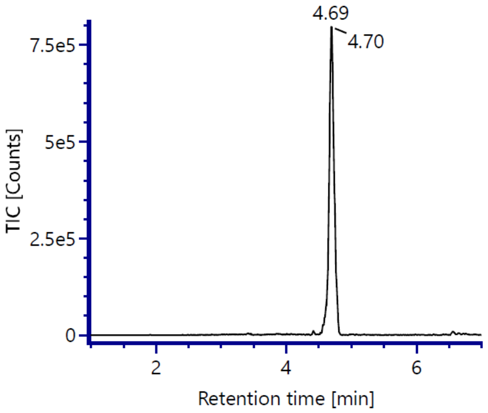


Item description: Channel name: Time 4.6943 +/- 0.0174 minutes

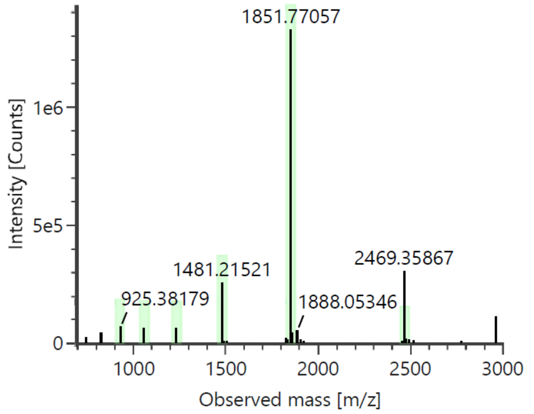

5'- fC*mU*fC-mG-fU-mC-fA-mU-fU-mA-fC-mC-fC-mA-fG-mC-fA-mG-fU-mG-fU-mU-fU (**P4**)

| Item name | Observed RT (min) | Neutral mass (Da) | Observed neutral mass (Da) | Observed m/z | Mass error (mDa) | Mass error (ppm) |
|---|---|---|---|---|---|---|
| EN12762-35-008 | 4.73 | 7428.01 | 7427.9846 | 1484.5896 | -25.3 | -3.4 |

Channel name: TUV 260 : Integrated : Smoothed

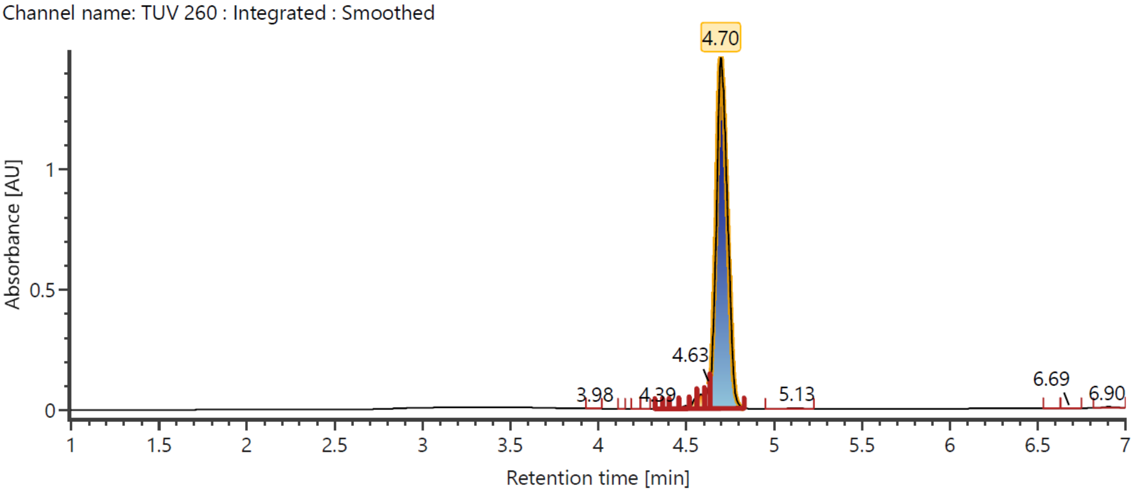


| . | Retention time (min) | Area | % Area (%) | Height |
|---|---|---|---|---|
| 1 | 4.70 | 6438563 | 94.33 | 1460535 |

**Item name: EN12762-35-008**
**Channel name: 1: TOF MS (400-5000) -40V ESI- (TIC)**

Item description: Channel name: Time 4.7266 +/- 0.0179 minutes

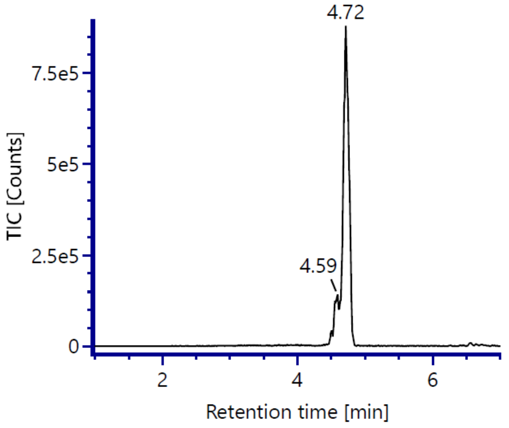


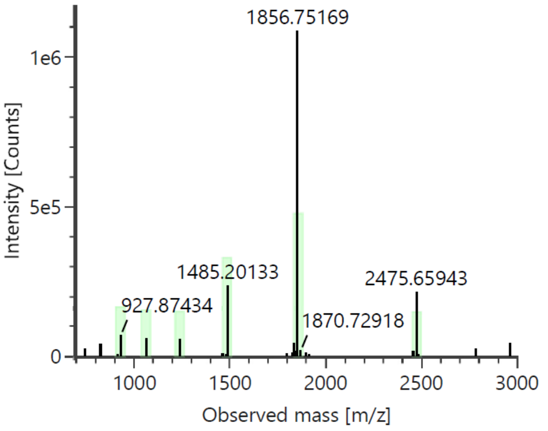

5'- fC*mU*fC-mG-fU-mC-fA-mU-fU-mA-fC-mC-fC-mA-fG-mC-fA-mG-fU-mG-fU*mU*fU (**P5**)

| Item name | Observed RT (min) | Neutral mass (Da) | Observed neutral mass (Da) | Observed m/z | Mass error (mDa) | Mass error (ppm) |
|---|---|---|---|---|---|---|
| EN12762-35-007 | 4.77 | 7459.96 | 7459.9193 | 1863.9725 | -44.9 | -6.0 |

Channel name: TUV 260 : Integrated : Smoothed

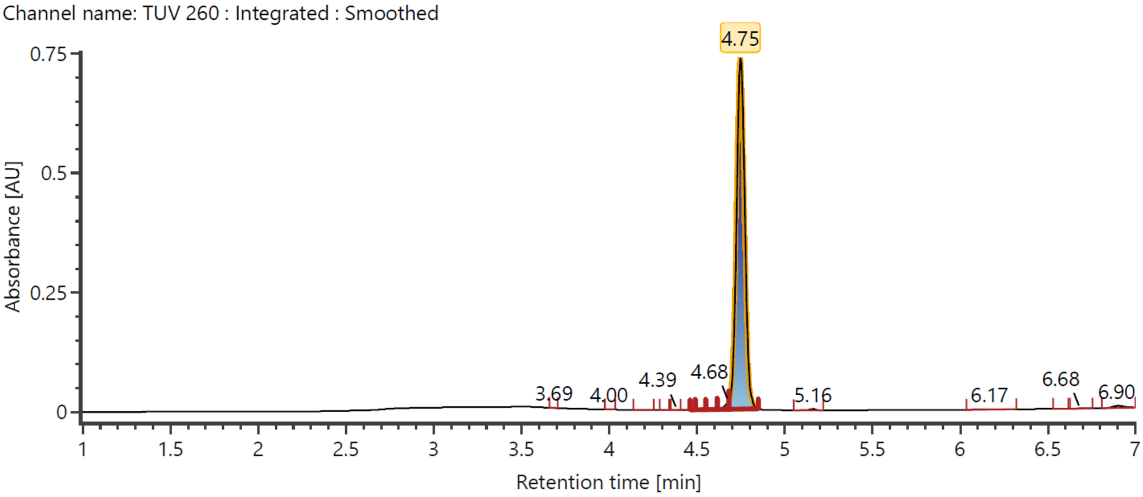


| . | Retention time (min) | Area | % Area (%) | Height |
|---|---|---|---|---|
| 1 | 4.75 | 2388980 | 97.29 | 733936 |

**Item name: EN12762-35-007**
**Channel name: 1: TOF MS (400-5000) -40V ESI- (TIC)**

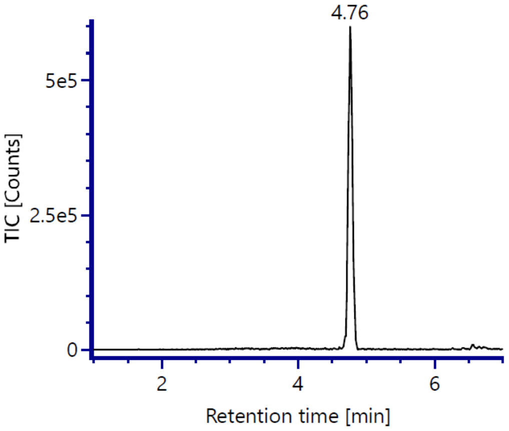


Item description: Channel name: Time 4.7696 +/- 0.0162 minutes

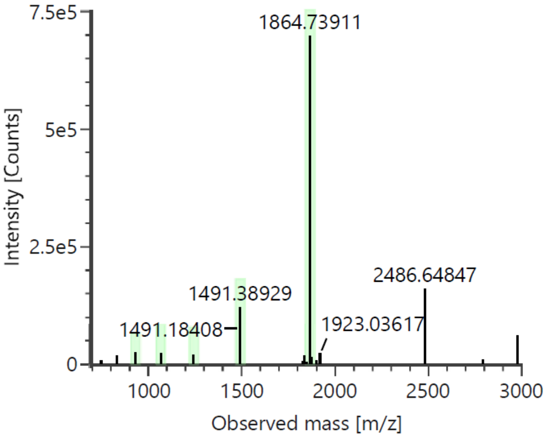

5'- fC-mU-fC-mG-fU-mC-mU-fU-mA-fC-mC-fC-mA-fG-mC-fA-mG-fU-mG-fU-mU-fU (**P6**)

| Item name | Observed RT (min) | Neutral mass (Da) | Observed neutral mass (Da) | Observed m/z | Mass error (mDa) | Mass error (ppm) |
|---|---|---|---|---|---|---|
| EN12762-35-004 | 3.55 | 7065.01 | 7064.9675 | 1765.2346 | -39.9 | -5.6 |

Channel name: TUV 260 : Integrated : Smoothed

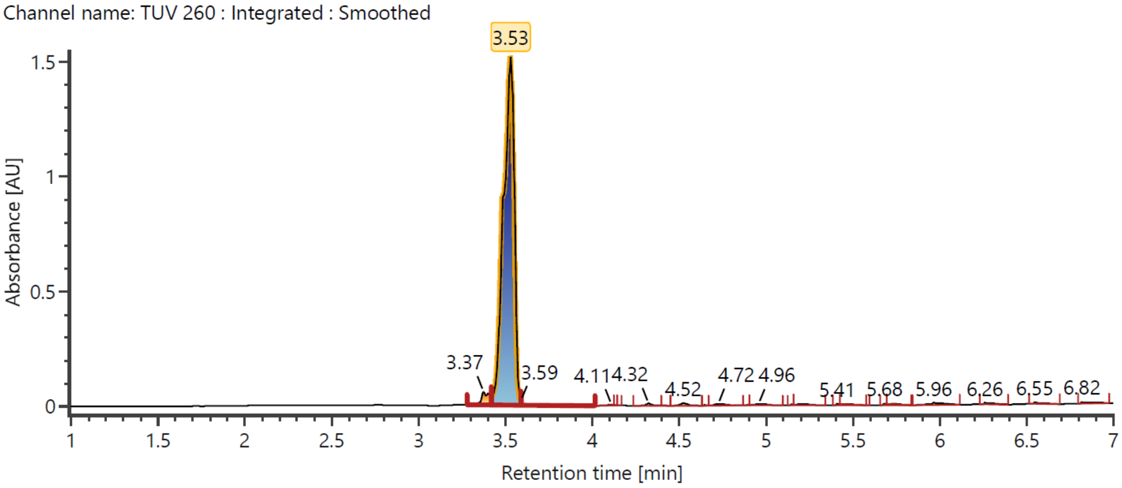


| . | Retention time (min) | Area | % Area (%) | Height |
|---|---|---|---|---|
| 1 | 3.53 | 6507141 | 91.65 | 1514860 |

**Item name: EN12762-35-004**
**Channel name: 1: TOF MS (400-5000) -40V ESI- (TIC)**

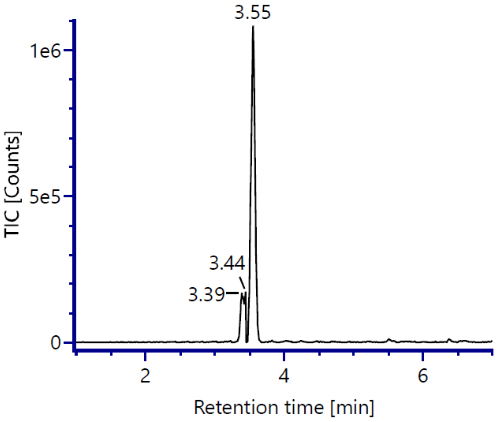


Item description: Channel name: Time 3.5540 +/- 0.0164 minutes

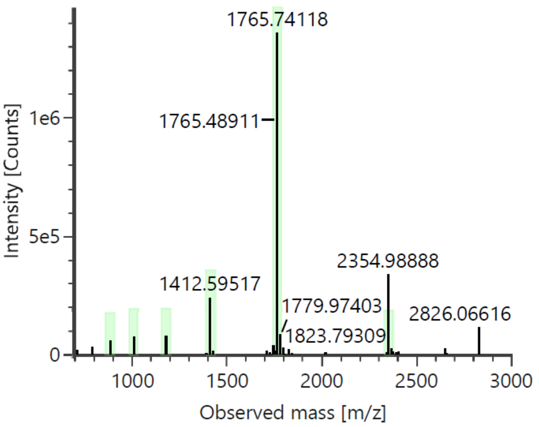

5’- fC-mU-fC-mG-fU-mC-fU-mU-fU-mA-fC-mC-fC-mA-fG-mC-fA-mG-fU-mG-fU-mU-fU (**P7**)

| Item name | Observed RT (min) | Neutral mass (Da) | Observed neutral mass (Da) | Observed m/z | Mass error (mDa) | Mass error (ppm) |
|---|---|---|---|---|---|---|
| EN12762-35-005 | 4.72 | 7373.03 | 7373.0019 | 1473.5931 | -26.5 | -3.6 |

Channel name: TUV 260 : Integrated : Smoothed

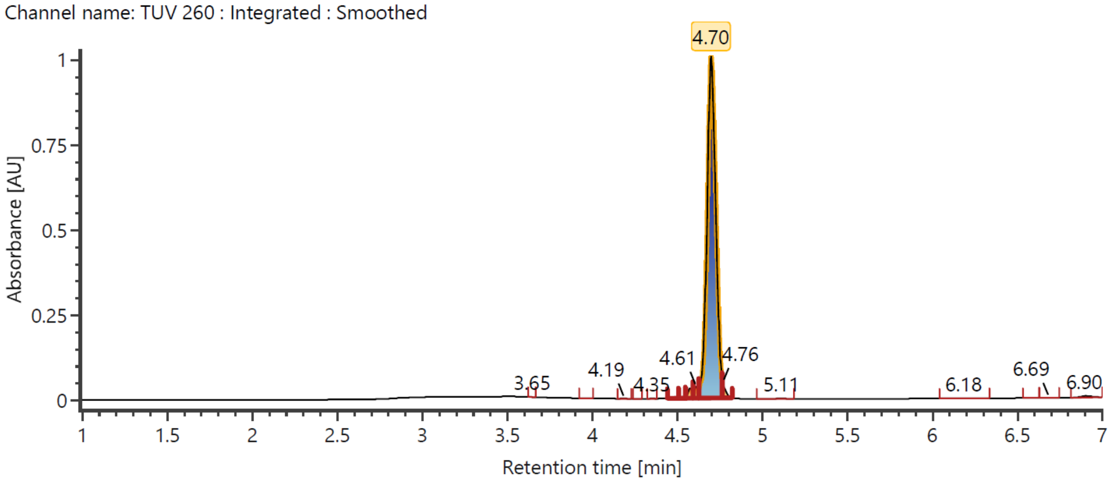


| . | Retention time (min) | Area | % Area (%) | Height |
|---|---|---|---|---|
| 1 | 4.70 | 3614756 | 94.63 | 1005397 |

**Item name: EN12762-35-005**
**Channel name: 1: TOF MS (400-5000) -40V ESI- (TIC)**

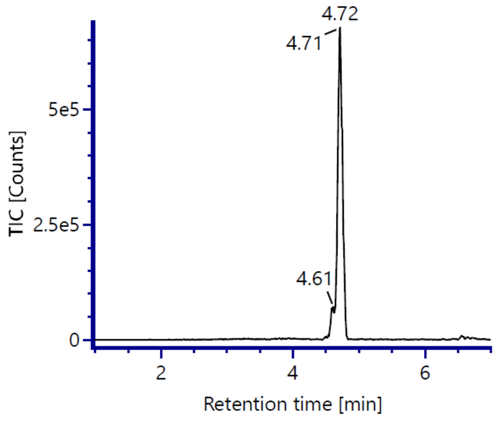


Item description: Channel name: Time 4.7152 +/- 0.0174 minutes

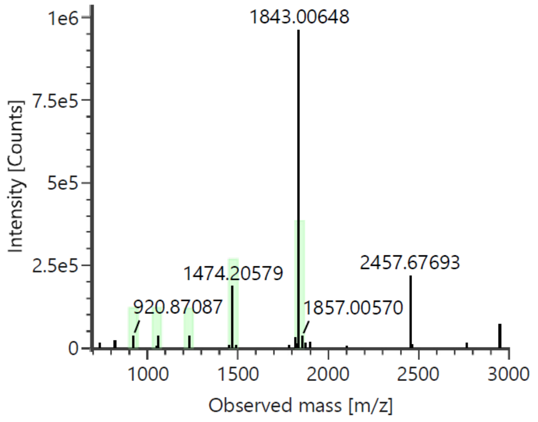

5'- fU-mC-fC-mA-fU-mC-fA-mU-fU-mA-fC-mC-fC-mG-fG-mC-fA-mG-fU-mA-fU-mU-fA (**P8**)

| Item name | Observed RT (min) | Neutral mass (Da) | Observed neutral mass (Da) | Observed m/z | Mass error (mDa) | Mass error (ppm) |
|---|---|---|---|---|---|---|
| EN12762-35-006 | 4.71 | 7403.09 | 7403.0575 | 1849.7571 | -30.4 | -4.1 |

Channel name: TUV 260 : Integrated : Smoothed

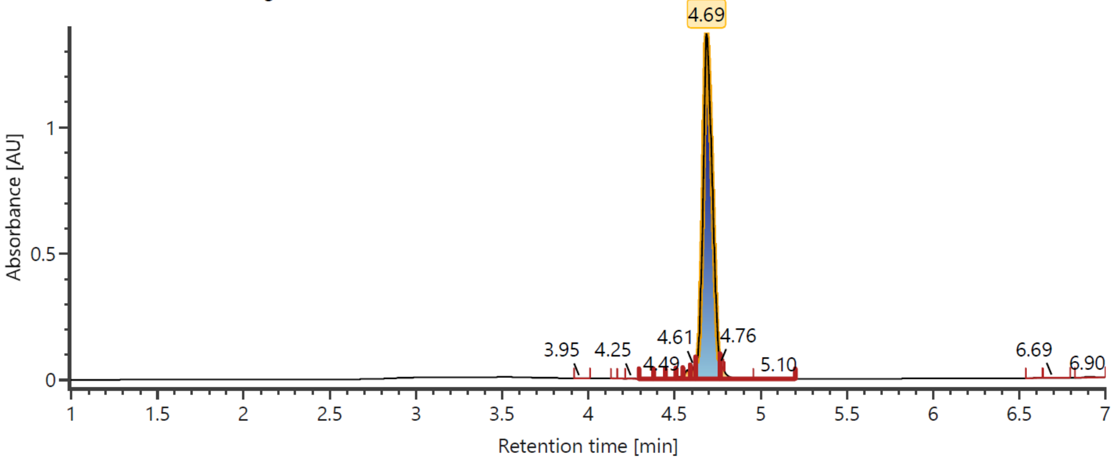


| . | Retention time (min) | Area | % Area (%) | Height |
|---|---|---|---|---|
| 1 | 4.69 | 5183161 | 94.67 | 1366613 |

**Item name: EN12762-35-006**
**Channel name: 1: TOF MS (400-5000) -40V ESI- (TIC)**

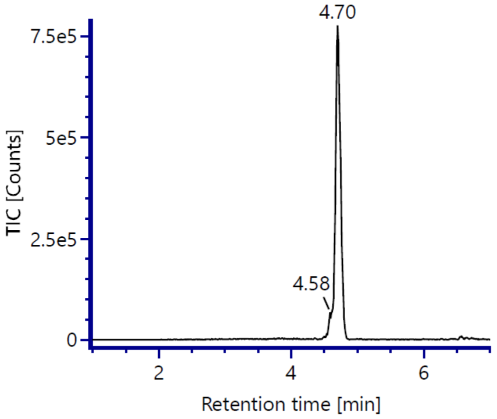


Item description: Channel name: Time 4.7067 +/- 0.0175 minutes

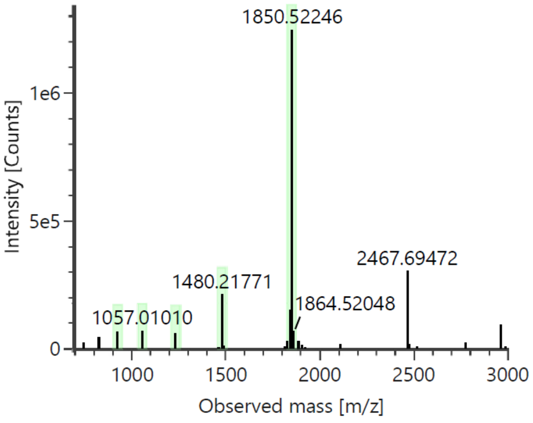

5'- fC-mG-fU-mC-fU-mU-fU-mA-fC-fC-fC-mA-fG-mC-fA-mG-fU-mG-fU-mU-fU (**P9**)

| Item name | Observed RT (min) | Neutral mass (Da) | Observed neutral mass (Da) | Observed m/z | Mass error (mDa) | Mass error (ppm) |
|---|---|---|---|---|---|---|
| EN12762-39-1 | 4.61 | 6733.93 | 6733.9436 | 1345.7814 | 13.1 | 1.9 |

Channel name: TUV 260 : Integrated : Smoothed

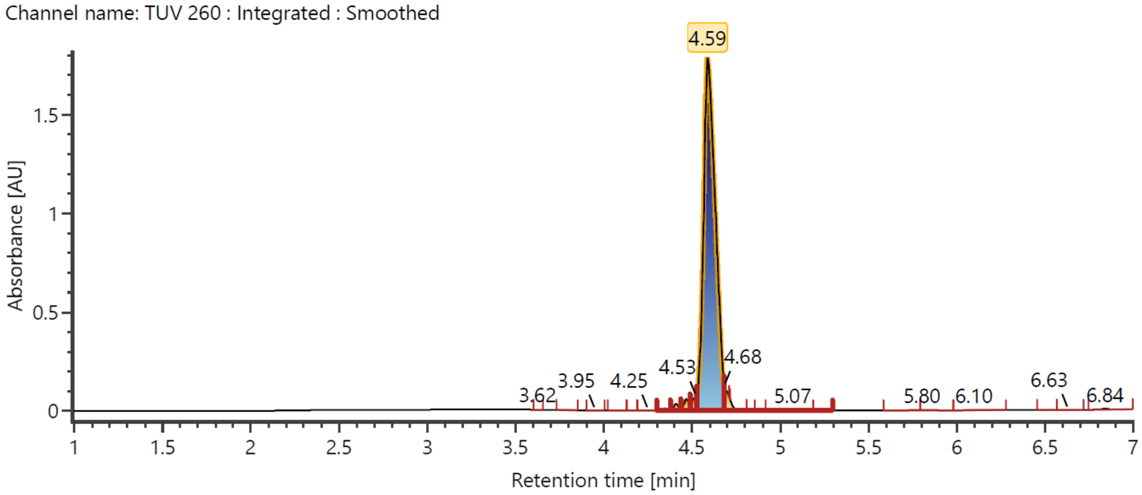


| . | Retention time (min) | Area | % Area (%) | Height |
|---|---|---|---|---|
| 1 | 4.59 | 8252357 | 93.08 | 1782812 |

**Item name: EN12762-39-1**
**Channel name: 1: TOF MS (400-5000) -40V ESI- (TIC)**

Item description: Channel name: Time 4.6110 +/- 0.0173 minutes

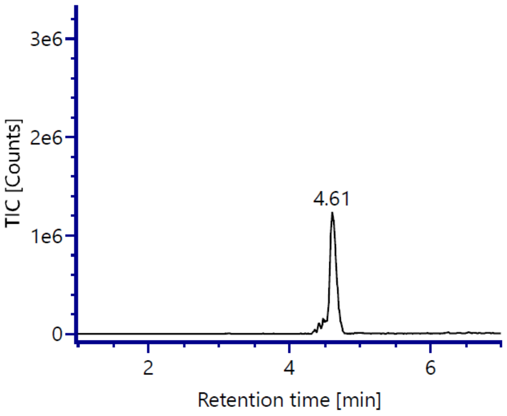


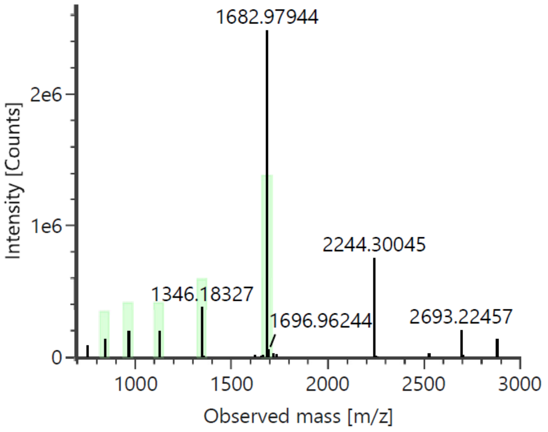

5’- fC*mG*fU-mC-fU-mU-fU-mA-fC-fC-fC-mA-fG-mC-fA-mG-fU-mG-fU-mU-fU (**P10**)

| Item name | Observed RT (min) | Neutral mass (Da) | Observed neutral mass (Da) | Observed m/z | Mass error (mDa) | Mass error (ppm) |
|---|---|---|---|---|---|---|
| EN12762-39-002 | 4.63 | 6765.88 | 6765.8906 | 1690.4654 | 5.8 | 0.9 |

Channel name: TUV 260 : Integrated : Smoothed

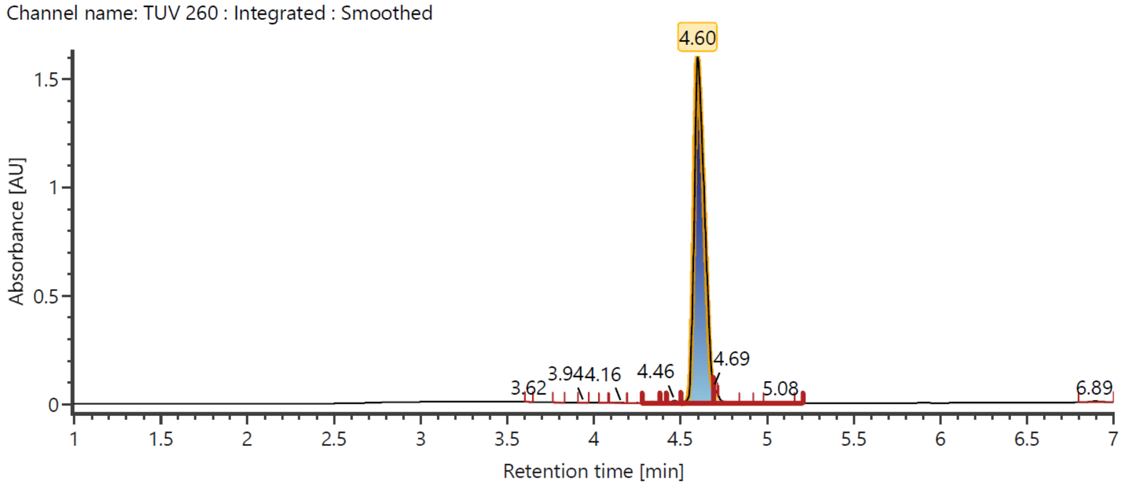


| . | Retention time (min) | Area | % Area (%) | Height |
|---|---|---|---|---|
| 1 | 4.60 | 6600923 | 96.77 | 1597755 |

**Item name: EN12762-39-002**
**Channel name: 1: TOF MS (400-5000) -40V ESI- (TIC)**

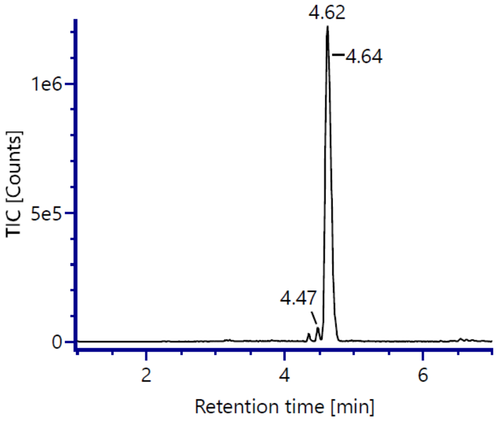


Item description: Channel name: Time 4.6285 +/- 0.0228 minutes

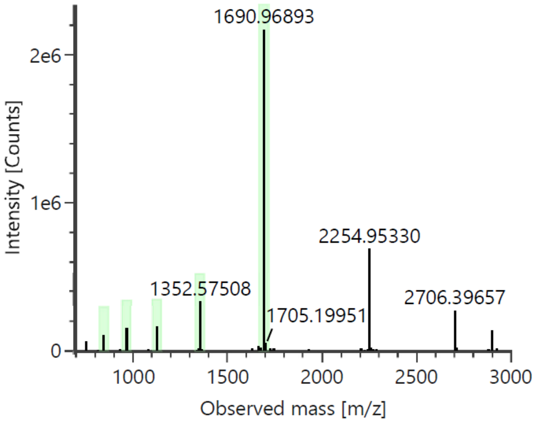

5'- mC*mG*mU-mC-mU-mU-fU-mA-fC-fC-fC-mA-mG-mC-mA-mG-mU-mG-mU-mU-mU (**P11**)

| Item name | Observed RT (min) | Neutral mass (Da) | Observed neutral mass (Da) | Observed m/z | Mass error (mDa) | Mass error (ppm) |
|---|---|---|---|---|---|---|
| EN12762-39-003 | 4.60 | 6862.04 | 6862.0414 | 1714.5031 | -3.3 | -0.5 |

Channel name: TUV 260 : Integrated : Smoothed

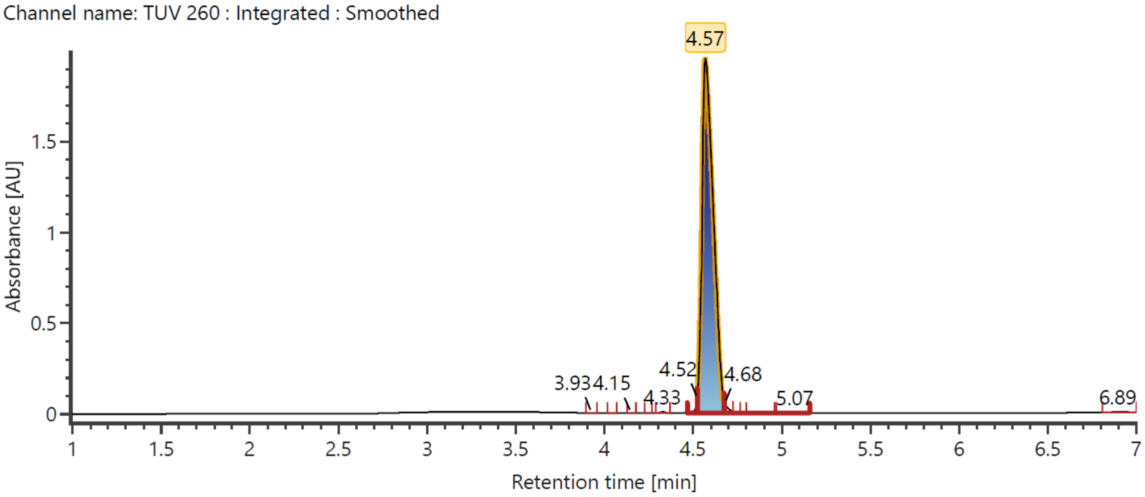


| . | Retention time (min) | Area | % Area (%) | Height |
|---|---|---|---|---|
| 1 | 4.57 | 8594094 | 97.94 | 1952396 |

**Item name: EN12762-39-003**
**Channel name: 1: TOF MS (400-5000) -40V ESI- (TIC)**

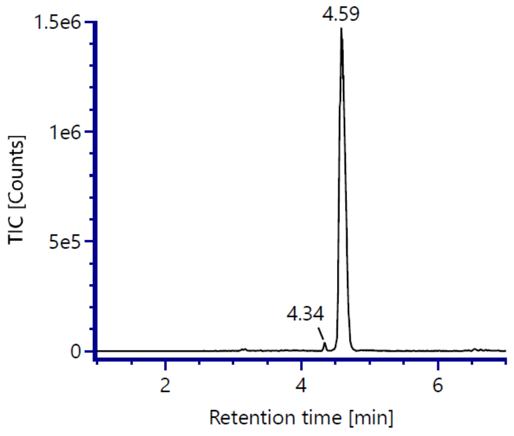


Item description: Channel name: Time 4.5978 +/- 0.0254 minutes

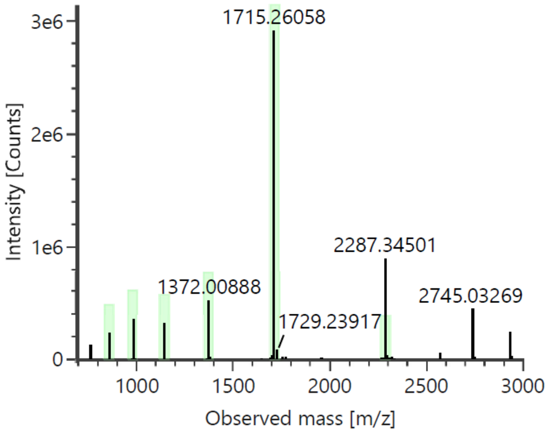

5’- fC*mU*fC-mG-fU-mC-fU-mU-fU-mA-fC-mC-fC-mA-fG-mC-fA-mG-fU-mG-fU*mU*fU (**P12**)

| Item name | Observed RT (min) | Neutral mass (Da) | Observed neutral mass (Da) | Observed m/z | Mass error (mDa) | Mass error (ppm) |
|---|---|---|---|---|---|---|
| EN12762-39-004 | 4.74 | 7436.94 | 7436.9151 | 1858.2215 | -21.9 | -2.9 |

Channel name: TUV 260 : Integrated : Smoothed

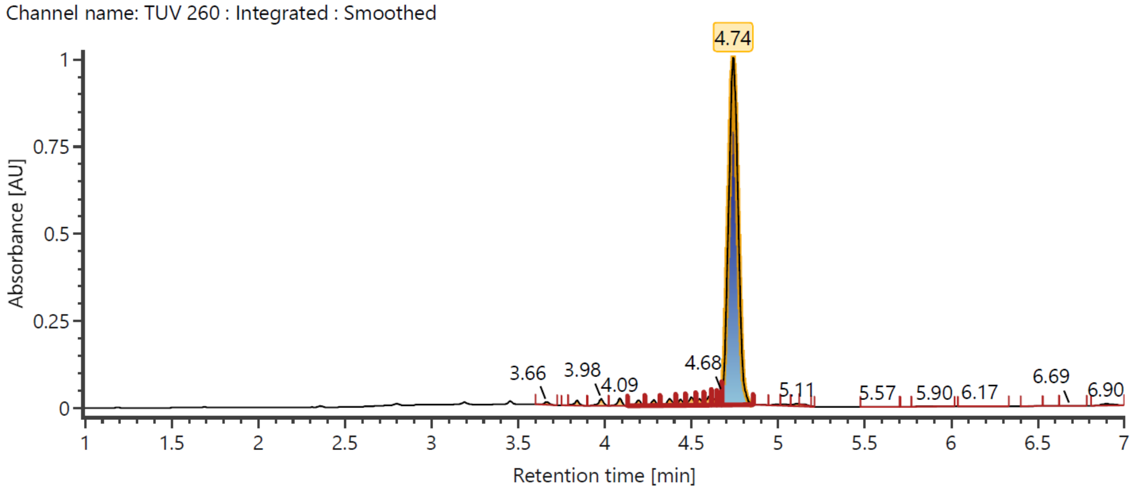


| . | Retention time (min) | Area | % Area (%) | Height |
|---|---|---|---|---|
| 1 | 4.74 | 3771763 | 87.23 | 996978 |

**Item name: EN12762-39-004**
**Channel name: 1: TOF MS (400-5000) -40V ESI- (TIC)**

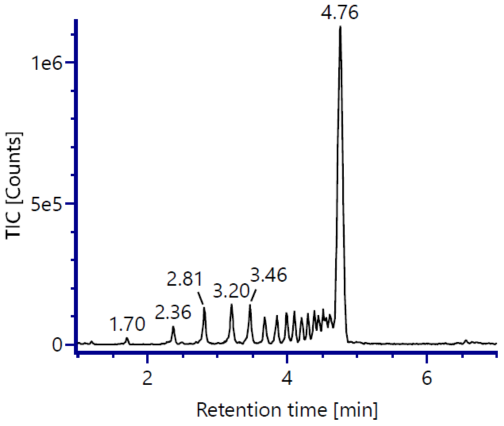


Item description: Channel name: Time 4.7388 +/- 0.0175 minutes

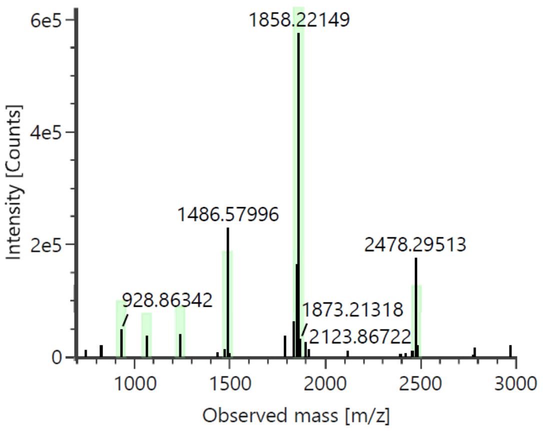

5'- mC*fU*mC-fG-mU-fC-mU-fU-mU-fA-mC-fC-mC-fA-mG-fC-mA-fG-mU-fG-mU*fU*mU (**P13**)

| Item name | Observed RT (min) | Neutral mass (Da) | Observed neutral mass (Da) | Observed m/z | Mass error (mDa) | Mass error (ppm) |
|---|---|---|---|---|---|---|
| EN12762-40-1 | 4.75 | 7457.93 | 7458.0361 | 1863.5018 | 101.3 | 13.6 |

Channel name: TUV 260 : Integrated : Smoothed

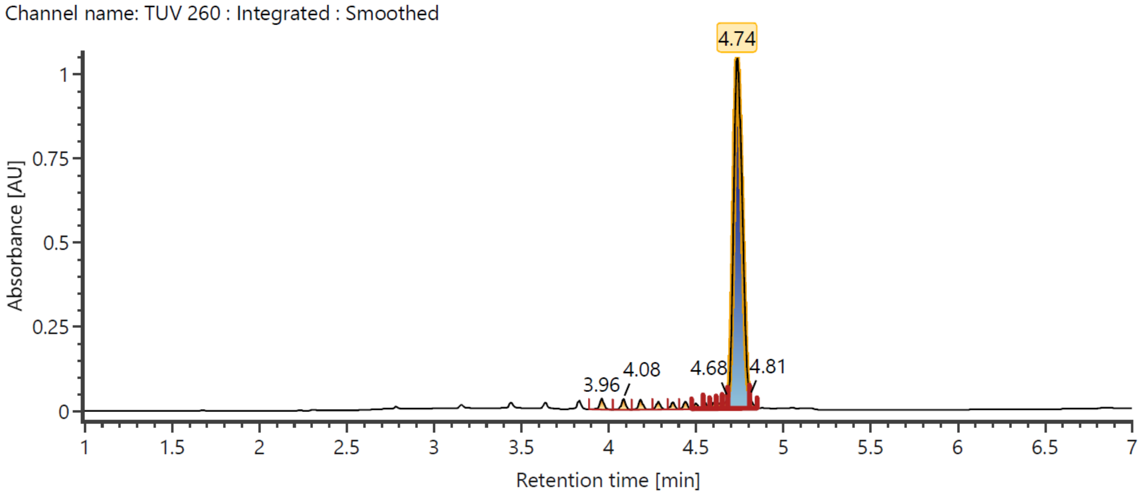


| . | Retention time (min) | Area | % Area (%) | Height |
|---|---|---|---|---|
| 1 | 4.74 | 3594248 | 89.31 | 1039636 |

**Item name: EN12762-40-1**
**Channel name: 1: TOF MS (400-5000) -40V ESI- (TIC)**

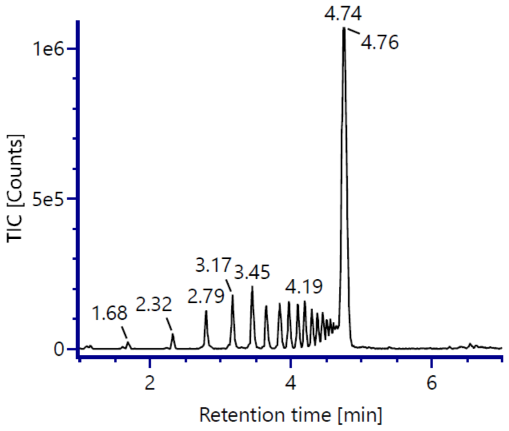


Item description: Channel name: Time 4.7534 +/- 0.0174 minutes

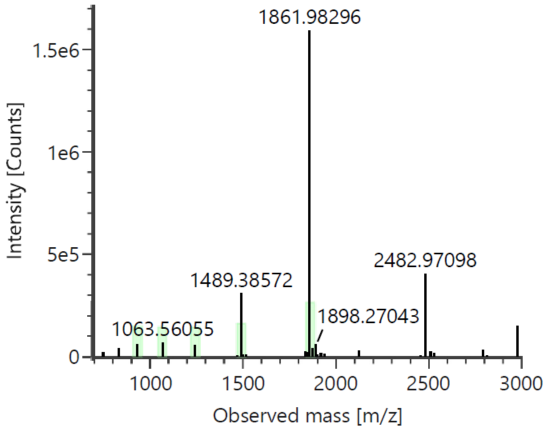

5’- fC-mG-fU-mC-fU-mU-fU-mA-fC-fC-fC-mA-fG-mC-fA-mG-fU-mG-fU-mU-fU-mG-mC-mA-mG-mC-mC-dG-dA-dA-dA-mG-mG-mC-mU-mG-mC (**P14**)

| Item name | Observed RT (min) | Neutral mass (Da) | Observed neutral mass (Da) | Observed m/z | Mass error (mDa) | Mass error (ppm) |
|---|---|---|---|---|---|---|
| EN12762-40-2 | 4.51 | 12055.87 | 12055.8678 | 2008.3040 | 2.8 | 0.2 |

Channel name: TUV 260 : Integrated : Smoothed

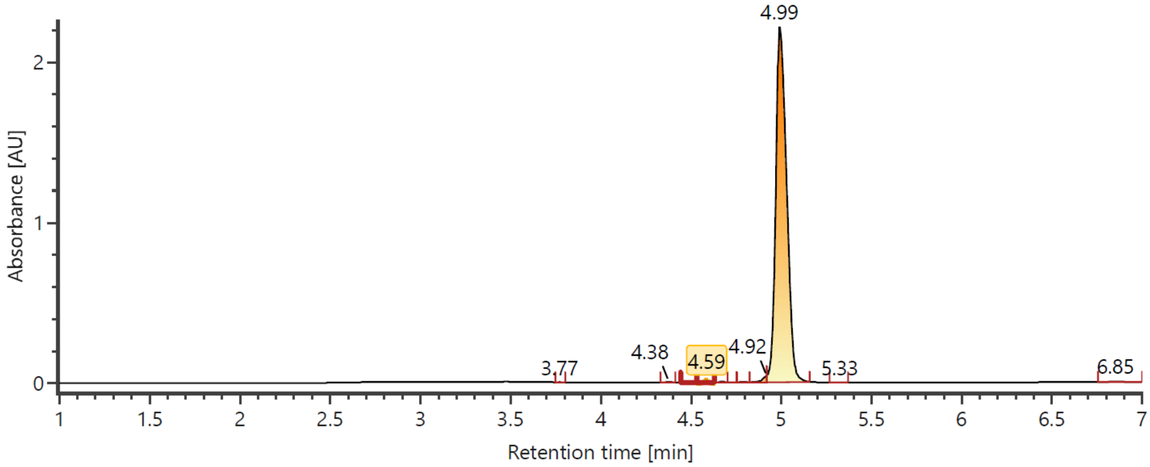


| . | Retention time (min) | Area | % Area (%) | Height |
|---|---|---|---|---|
| 1 | 4.59 | 19518 | 0.21 | 9477 |

**Item name: EN12762-40-2**
**Channel name: 1: TOF MS (400-5000) -40V ESI- (TIC)**

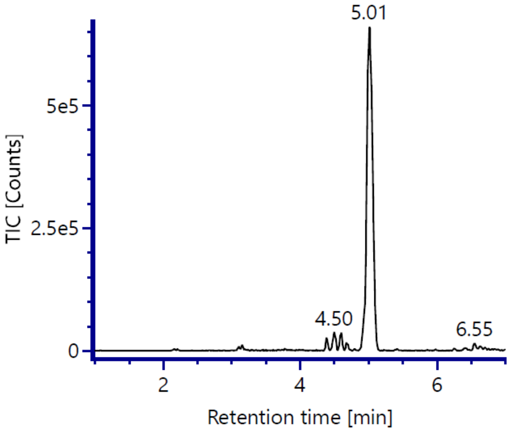


Item description: Channel name: Time 4.5096 +/- 0.0186 minutes

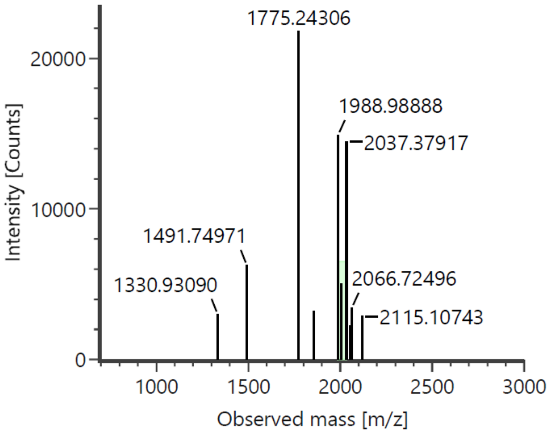

5’- mA-fG-mC-fA-mG-fU-mG-fU-mU-fU (**P15**)

| Item name | Observed RT (min) | Neutral mass (Da) | Observed neutral mass (Da) | Observed m/z | Mass error (mDa) | Mass error (ppm) |
|---|---|---|---|---|---|---|
| EN12762-40-003 | 3.73 | 3240.49 | 3240.4901 | 1079.1561 | -0.6 | -0.2 |

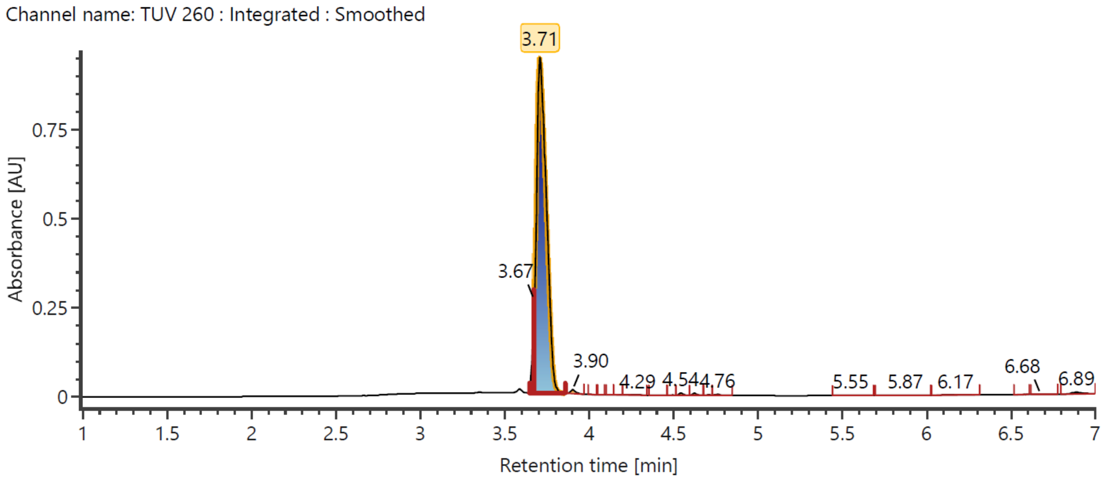


| . | Retention time (min) | Area | % Area (%) | Height |
|---|---|---|---|---|
| 1 | 3.71 | 3780554 | 93.30 | 941513 |

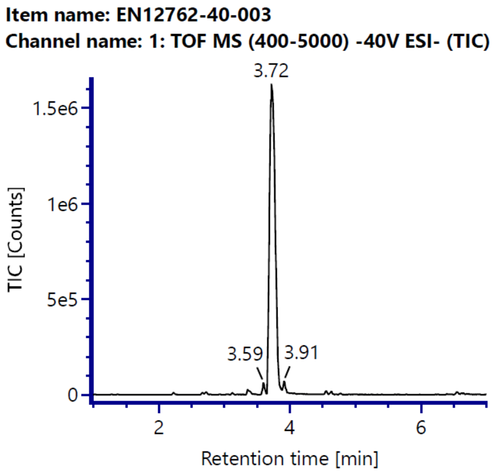


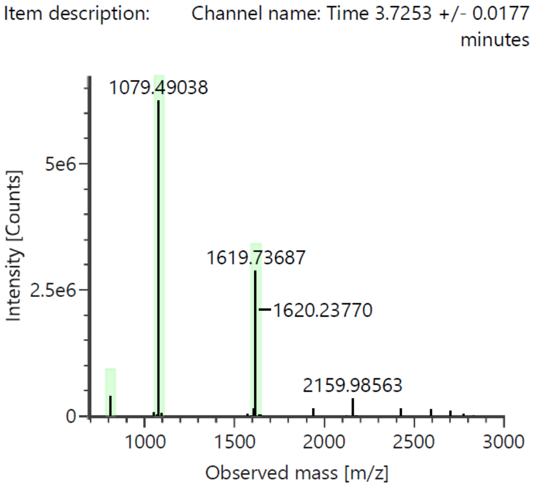

5'- mU-fU-mA-fC-mC-fC-mA-fG-mC-fA-mG-fU-mG-fU-mU-fU (**P16**)

| Item name | Observed RT (min) | Neutral mass (Da) | Observed neutral mass (Da) | Observed m/z | Mass error (mDa) | Mass error (ppm) |
|---|---|---|---|---|---|---|
| EN12762-40-004 | 4.35 | 5144.75 | 5144.7518 | 1713.9100 | 0.3 | 0.1 |

Channel name: TUV 260 : Integrated : Smoothed

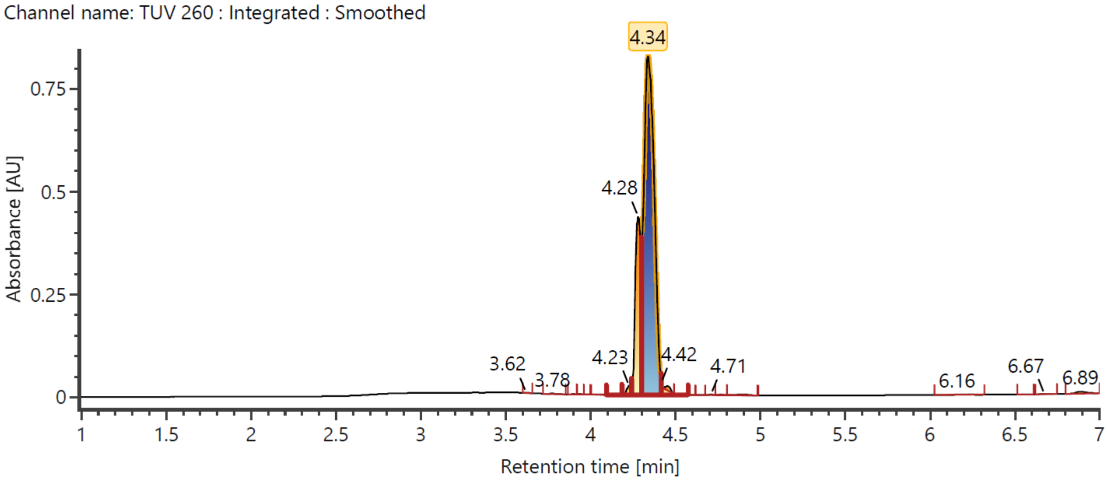


| . | Retention time (min) | Area | % Area (%) | Height |
|---|---|---|---|---|
| 1 | 4.34 | 3479148 | 74.92 | 823579 |

**Item name: EN12762-40-004**
**Channel name: 1: TOF MS (400-5000) -40V ESI- (TIC)**

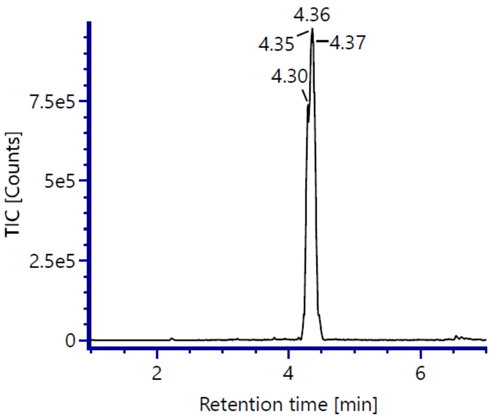


Item description: Channel name: Time 4.3466 +/- 0.0200 minutes

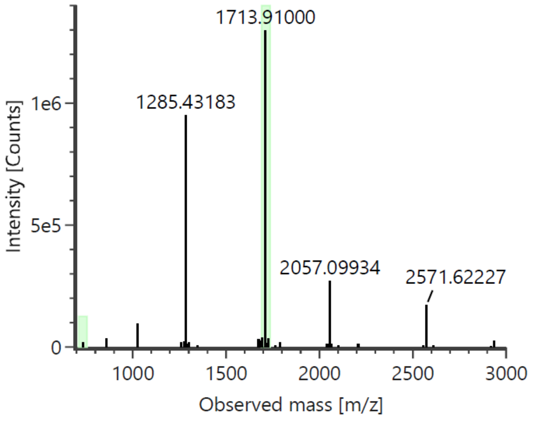

5'- fC-mG-fU-mC-fU-mU-fU-mA-fC-mC-fC-mA-fG-mC-fA-mG-fU-mG-fU-mU-fU (**P17**)

| Item name | Observed RT (min) | Neutral mass (Da) | Observed neutral mass (Da) | Observed m/z | Mass error (mDa) | Mass error (ppm) |
|---|---|---|---|---|---|---|
| EN12762-41-001 | 4.62 | 6745.95 | 6745.9105 | 2247.6296 | -39.9 | -5.9 |

Channel name: TUV 260 : Integrated : Smoothed

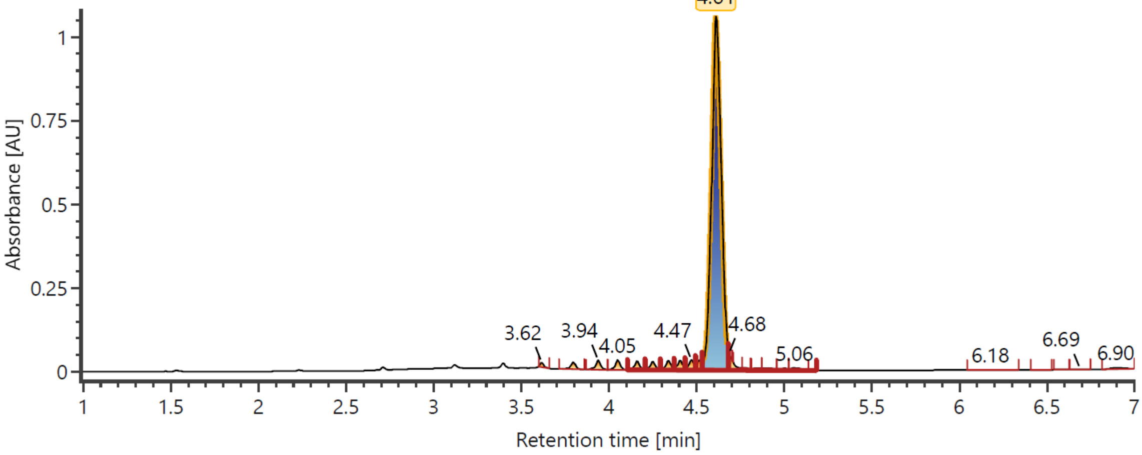


| . | Retention time (min) | Area | % Area (%) | Height |
|---|---|---|---|---|
| 1 | 4.61 | 4053235 | 84.66 | 1056791 |

**Item name: EN12762-41-001**
**Channel name: 1: TOF MS (400-5000) -40V ESI- (TIC)**

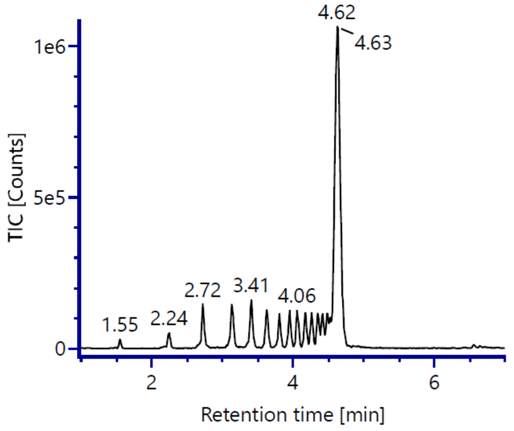


Item description: Channel name: Time 4.6160 +/- 0.0179 minutes

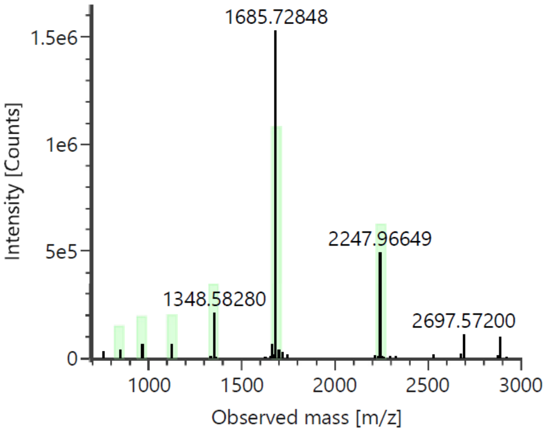

5'- mA-fG-mC-fA-mG-fU-mG-fU-mU-fU fC-mC-fC-mG-fU-mC-fU-mU-fU-mA-fC-mC-fC (**P18**)

| Item name | Observed RT (min) | Neutral mass (Da) | Observed neutral mass (Da) | Observed m/z | Mass error (mDa) | Mass error (ppm) |
|---|---|---|---|---|---|---|
| EN12762-41-2 | 4.17 | 4069.60 | 4069.6140 | 1355.5307 | 16.1 | 4.0 |

Channel name: TUV 260 : Integrated : Smoothed

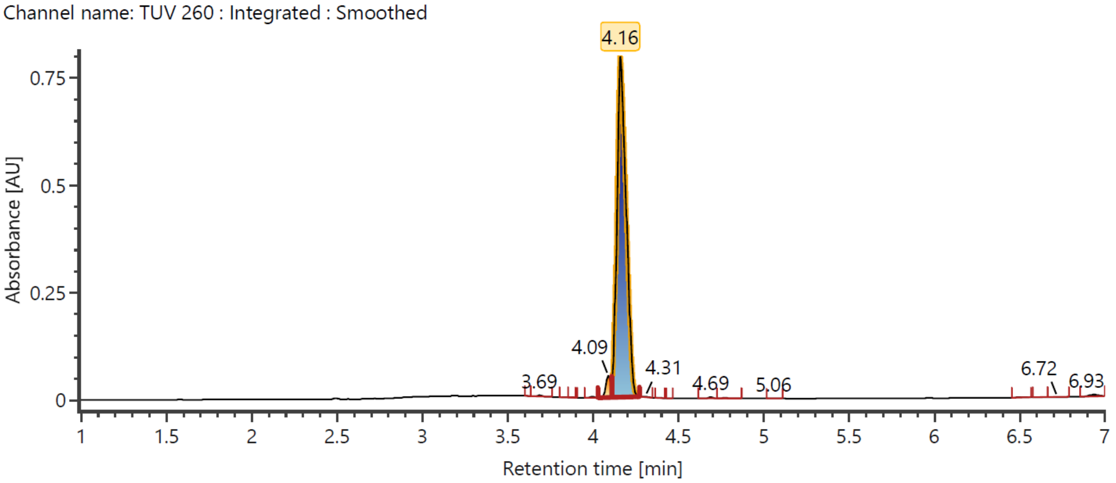


| . | Retention time (min) | Area | % Area (%) | Height |
|---|---|---|---|---|
| 1 | 4.16 | 2972251 | 95.34 | 792959 |

**Item name: EN12762-41-2**
**Channel name: 1: TOF MS (400-5000) -40V ESI- (TIC)**

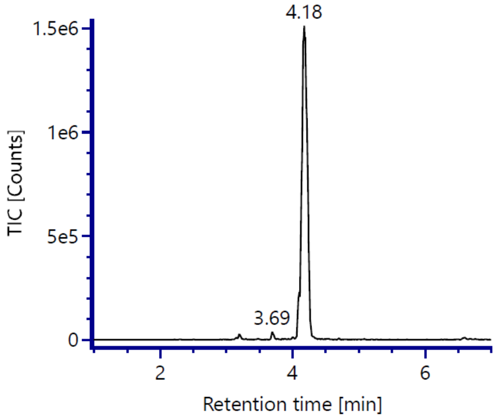


Item description: Channel name: Time 4.1747 +/- 0.0166 minutes

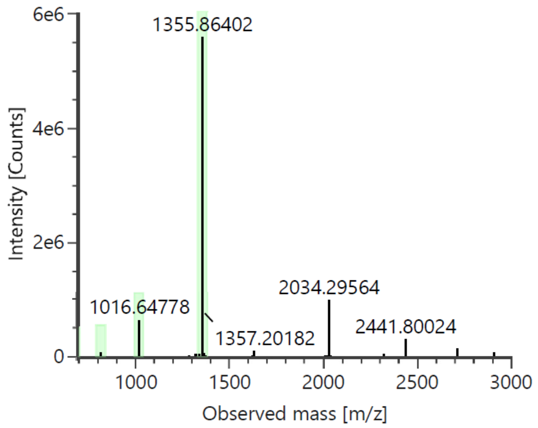

5'- mC-rG-mU-mC-mU-mU-mU-rA-mC-mC-mC-rA-rG-mC-rA-rG-mU-rG-mU-mU-mU (**P19**)

| Item name | Observed RT (min) | Neutral mass (Da) | Observed neutral mass (Da) | Observed m/z | Mass error (mDa) | Mass error (ppm) |
|---|---|---|---|---|---|---|
| EN12762-41-3 | 4.59 | 6780.06 | 6780.0331 | 1694.0010 | -27.7 | -4.1 |

Channel name: TUV 260 : Integrated : Smoothed

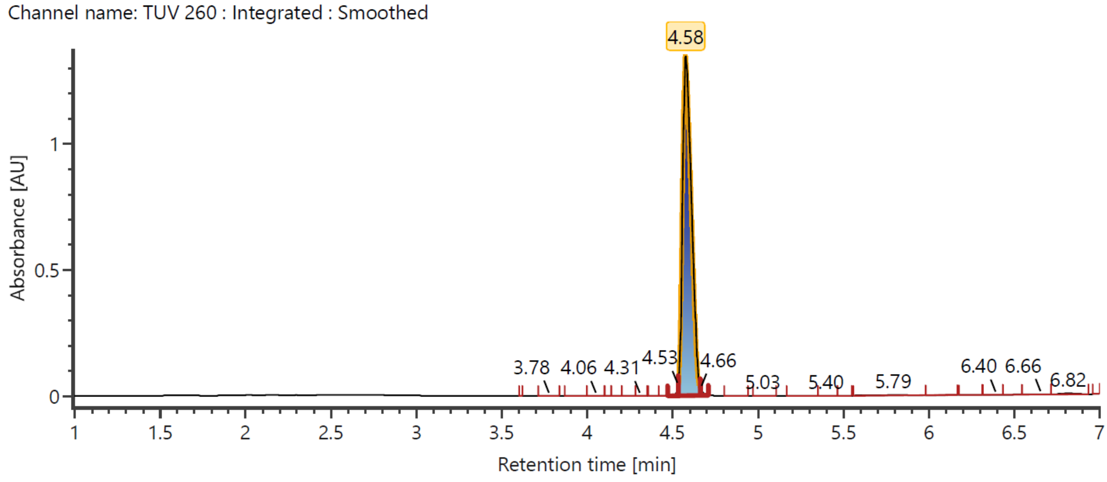


| . | Retention time (min) | Area | % Area (%) | Height |
|---|---|---|---|---|
| 1 | 4.58 | 4873479 | 97.76 | 1346461 |

**Item name: EN12762-41-3**
**Channel name: 1: TOF MS (400-5000) -40V ESI- (TIC)**

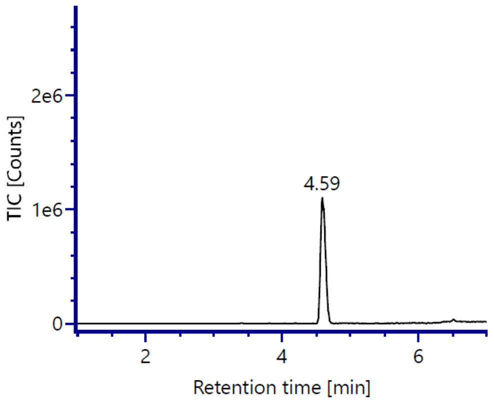


Item description: Channel name: Time 4.5924 +/- 0.0190 minutes

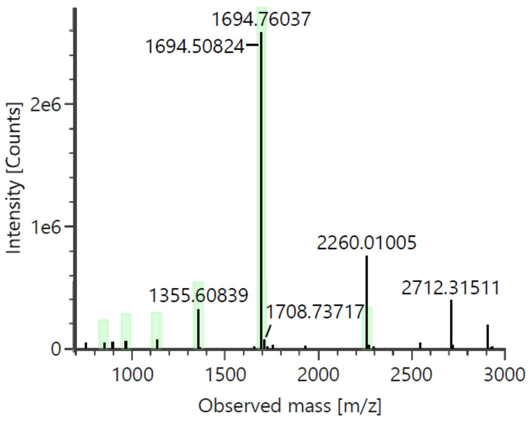

5'- rC-rU-rC-rG-rU-rC-rU-rU-rU-rA-rC-rC-rC-rA-rG-rC-rA-rG-rU-rG-rU-rU-rU (**P20**)

| Item name | Observed RT (min) | Neutral mass (Da) | Observed neutral mass (Da) | Observed m/z | Mass error (mDa) | Mass error (ppm) |
|---|---|---|---|---|---|---|
| EN12762-70-1 | 4.70 | 7194.91 | 7194.8689 | 1797.7100 | -39.3 | -5.5 |

Channel name: TUV 260 : Integrated : Smoothed

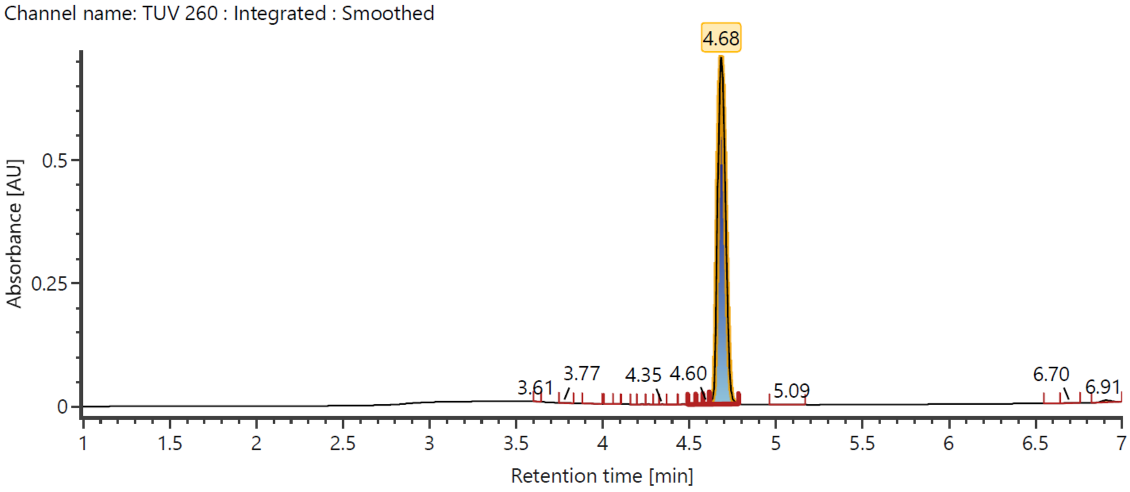


| . | Retention time (min) | Area | % Area (%) | Height |
|---|---|---|---|---|
| 1 | 4.68 | 2233144 | 98.52 | 702557 |

**Item name: EN12762-70-1**
**Channel name: 1: TOF MS (400-5000) -40V ESI- (TIC)**

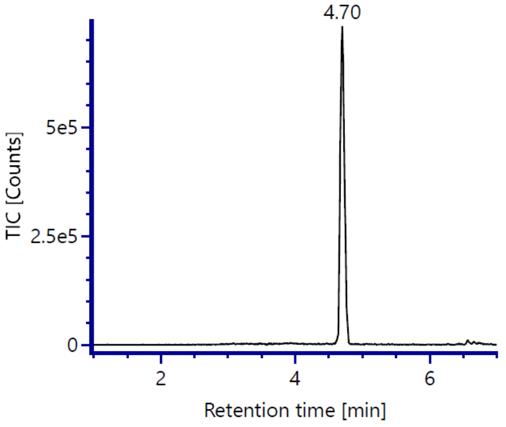


Item description: Channel name: Time 4.6994 +/- 0.0205 minutes

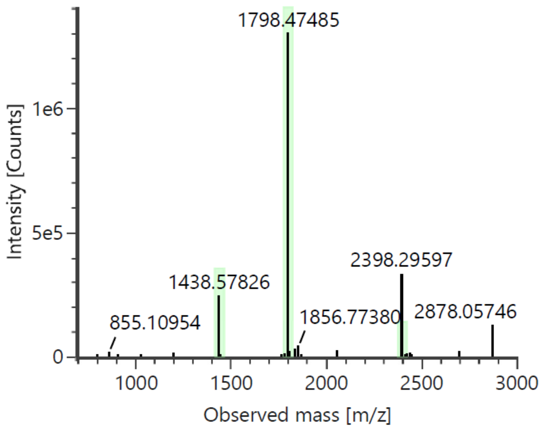

5'- rC-mG-rU-rC-rU-rU-rU-mA-rC-rC-rC-mA-mG-rC-mA-mG-rU-mG-rU-rU-rU (**P21**)

| Item name | Observed RT (min) | Neutral mass (Da) | Observed neutral mass (Da) | Observed m/z | Mass error (mDa) | Mass error (ppm) |
|---|---|---|---|---|---|---|
| EN12762-70-2 | 4.62 | 6681.95 | 6681.9467 | 1669.4794 | -4.5 | -0.7 |

Channel name: TUV 260 : Integrated : Smoothed

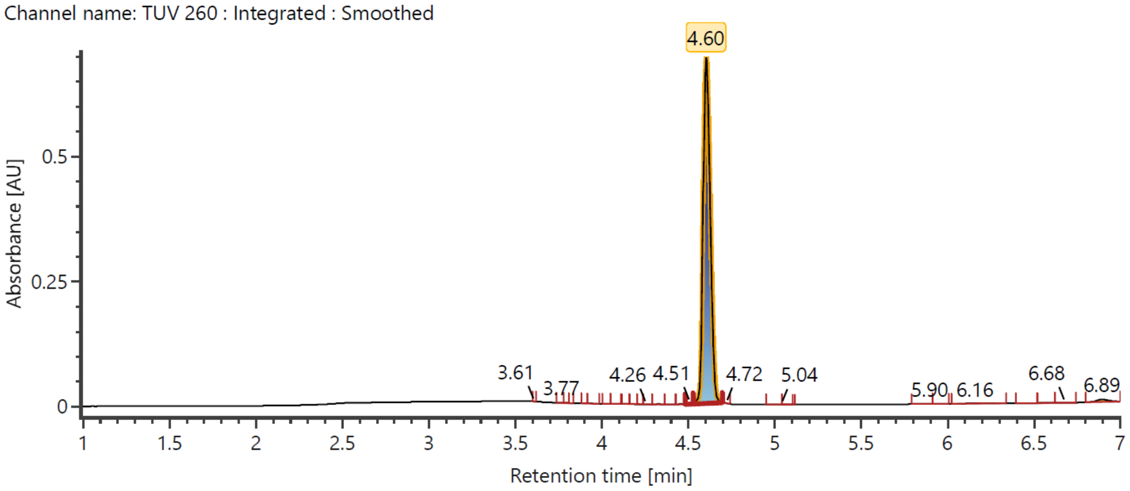


| . | Retention time (min) | Area | % Area (%) | Height |
|---|---|---|---|---|
| 1 | 4.60 | 1970518 | 97.82 | 691726 |

**Item name: EN12762-70-2**
**Channel name: 1: TOF MS (400-5000) -40V ESI- (TIC)**

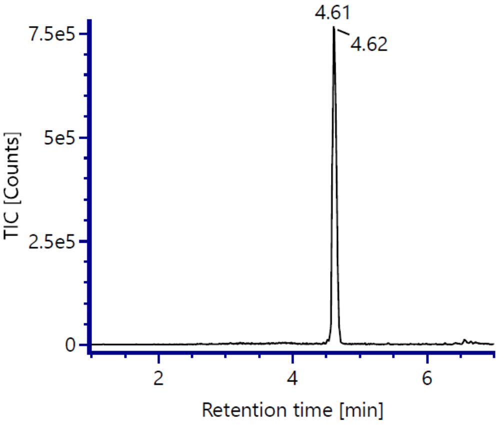


Item description: Channel name: Time 4.6167 +/- 0.0166 minutes

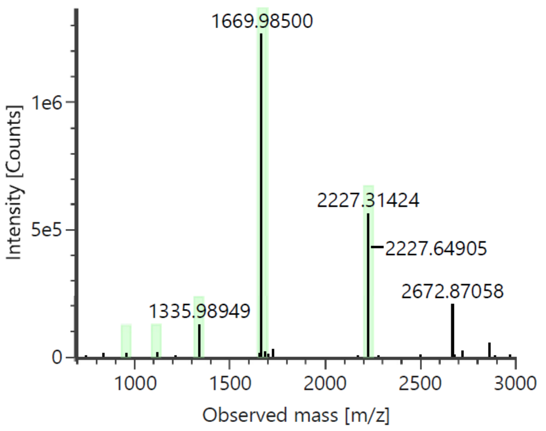

5'- fC-rG-fU-fC-fU-fU-fU-rA-fC-fC-fC-rA-rG-fC-rA-rG-fU-rG-fU-fU-fU (**P22**)

| Item name | Observed RT (min) | Neutral mass (Da) | Observed neutral mass (Da) | Observed m/z | Mass error (mDa) | Mass error (ppm) |
|---|---|---|---|---|---|---|
| EN12762-70-3 | 4.68 | 6611.78 | 6611.8012 | 1651.9430 | 20.2 | 3.1 |

Channel name: TUV 260 : Integrated : Smoothed

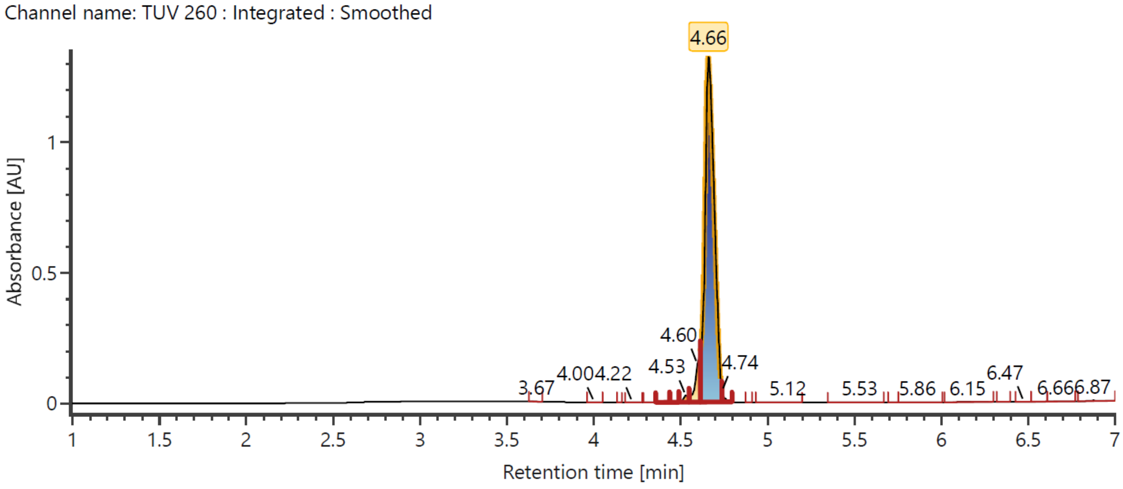


| . | Retention time (min) | Area | % Area (%) | Height |
|---|---|---|---|---|
| 1 | 4.66 | 4874990 | 91.23 | 1322359 |

**Item name: EN12762-70-3**
**Channel name: 1: TOF MS (400-5000) -40V ESI- (TIC)**

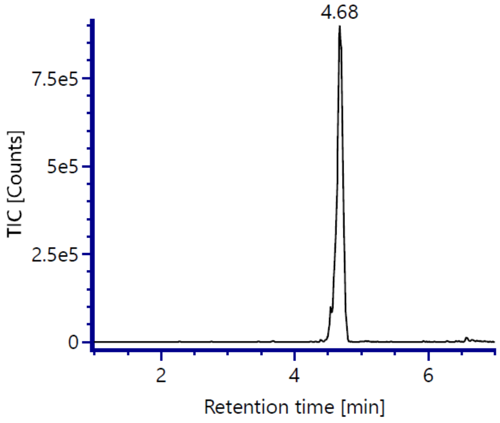


Item description: Channel name: Time 4.6814 +/- 0.0179 minutes

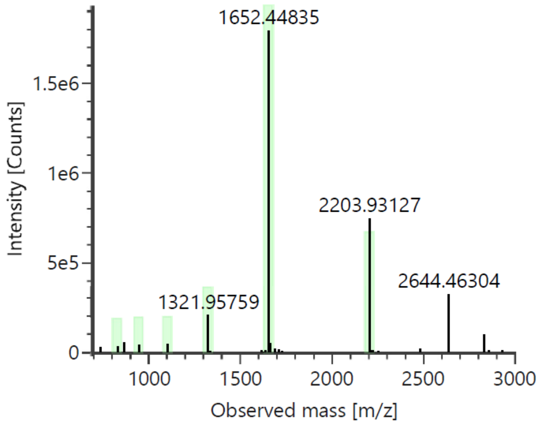

5’- mU-mU-rA-mC-mC-mC-rA-rG-mC-rA-rG-mU-rG-mU-mU-mU (**P23**)

| Item name | Observed RT (min) | Neutral mass (Da) | Observed neutral mass (Da) | Observed m/z | Mass error (mDa) | Mass error (ppm) |
|---|---|---|---|---|---|---|
| EN12762-71-1 | 4.34 | 5156.82 | 5156.8554 | 1717.9445 | 37.8 | 7.3 |

Channel name: TUV 260 : Integrated : Smoothed

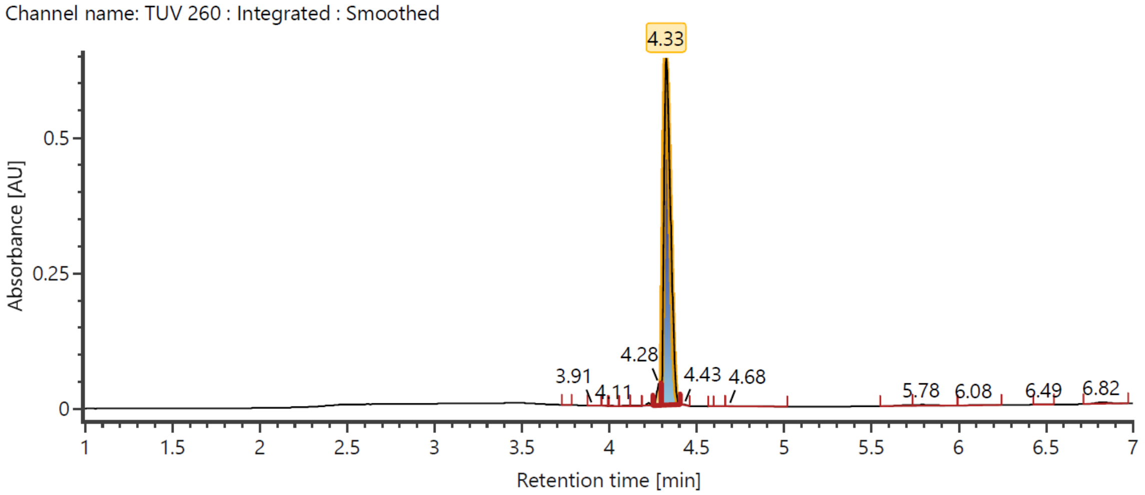


| . | Retention time (min) | Area | % Area (%) | Height |
|---|---|---|---|---|
| 1 | 4.33 | 1673359 | 93.28 | 639775 |

**Item name: EN12762-71-1**
**Channel name: 1: TOF MS (400-5000) -40V ESI- (TIC)**

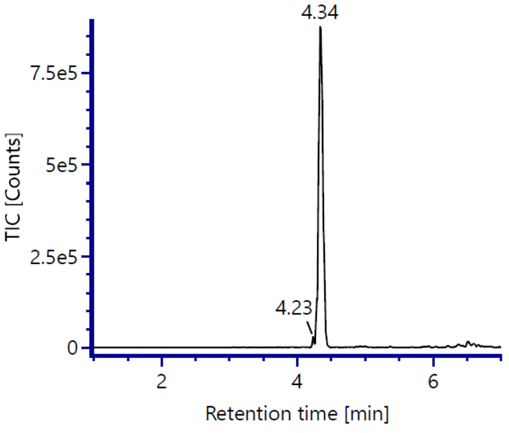


Item description: Channel name: Time 4.3390 +/- 0.0168 minutes

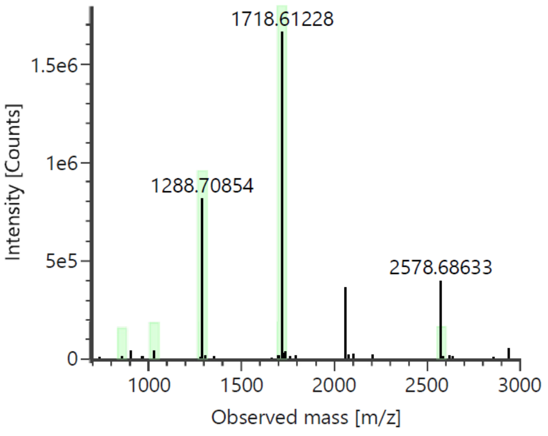

5'- mU*mU*rA-mC-mC-mC-rA-rG-mC-rA-rG-mU-rG-mU*mU*mU (**P24**)

| Item name | Observed RT (min) | Neutral mass (Da) | Observed neutral mass (Da) | Observed m/z | Mass error (mDa) | Mass error (ppm) |
|---|---|---|---|---|---|---|
| EN12762-71-2 | 4.36 | 5220.73 | 5220.7409 | 1739.2397 | 14.7 | 2.8 |

Channel name: TUV 260 : Integrated : Smoothed

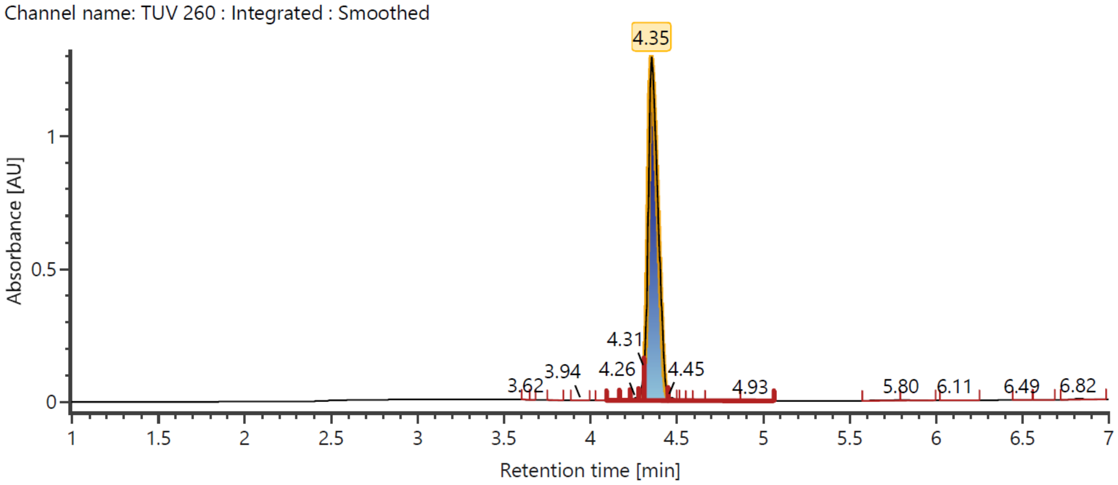


| . | Retention time (min) | Area | % Area (%) | Height |
|---|---|---|---|---|
| 1 | 4.35 | 5049481 | 96.05 | 1292614 |

**Item name: EN12762-71-2**
**Channel name: 1: TOF MS (400-5000) -40V ESI- (TIC)**

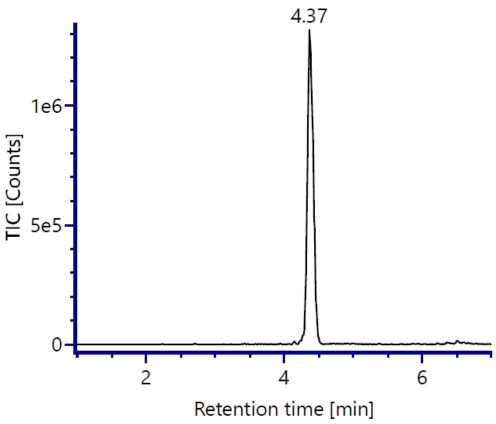


Item description: Channel name: Time 4.3644 +/- 0.0168 minutes

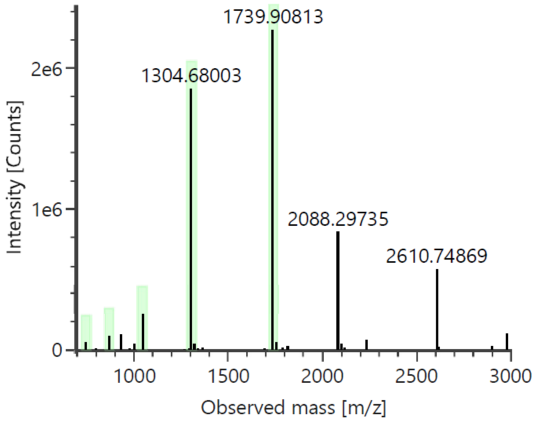

5'- mC*rG*mU-mC-mU-mU-mU-rA-mC-mC-mC-rA-rG-mC-rA-rG-mU-rG-mU-mU-mU (**P25**)

| Item name | Observed RT (min) | Neutral mass (Da) | Observed neutral mass (Da) | Observed m/z | Mass error (mDa) | Mass error (ppm) |
|---|---|---|---|---|---|---|
| EN12762-72-1 | 4.59 | 6812.02 | 6812.0239 | 1701.9987 | 8.8 | 1.3 |

Channel name: TUV 260 : Integrated : Smoothed

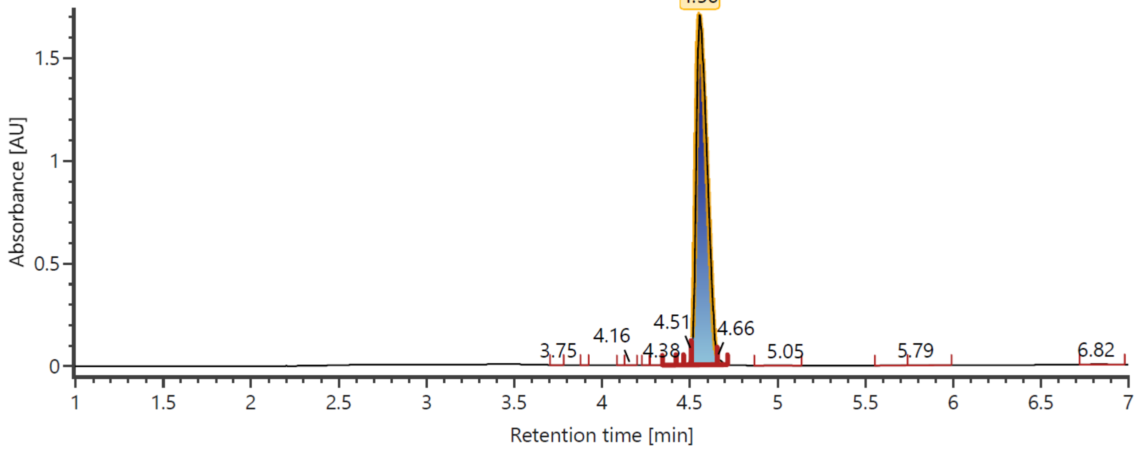


| . | Retention time (min) | Area | % Area (%) | Height |
|---|---|---|---|---|
| 1 | 4.56 | 7344249 | 98.11 | 1700763 |

**Item name: EN12762-72-1**
**Channel name: 1: TOF MS (400-5000) -40V ESI- (TIC)**

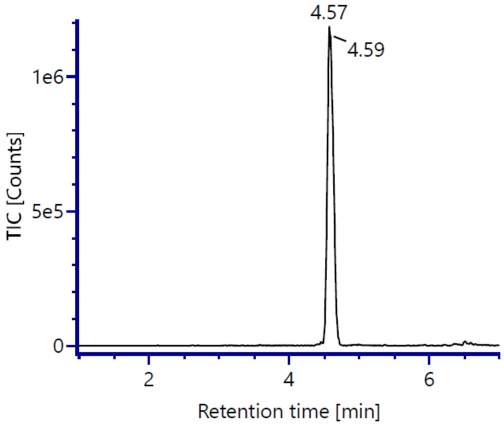


Item description: Channel name: Time 4.5929 +/- 0.0203 minutes

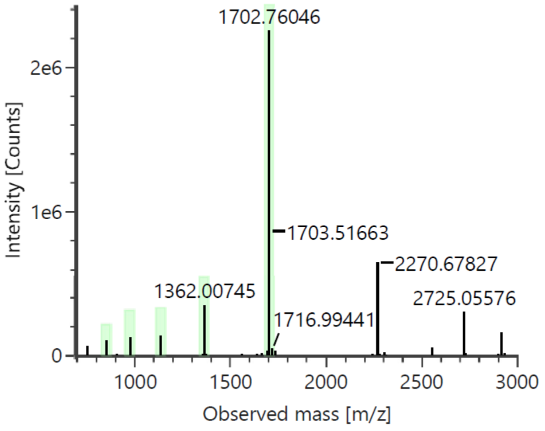

5’- mC*rG*mU-mC-mU-mU-mU-rA-mC-mC-mC-rA-rG-mC-rA-rG-mU-rG-mU*mU*mU (**P26**)

| Item name | Observed RT (min) | Neutral mass (Da) | Observed neutral mass (Da) | Observed m/z | Mass error (mDa) | Mass error (ppm) |
|---|---|---|---|---|---|---|
| EN12762-72-2 | 4.63 | 6843.97 | 6843.9790 | 1709.9875 | 9.6 | 1.4 |

Channel name: TUV 260 : Integrated : Smoothed

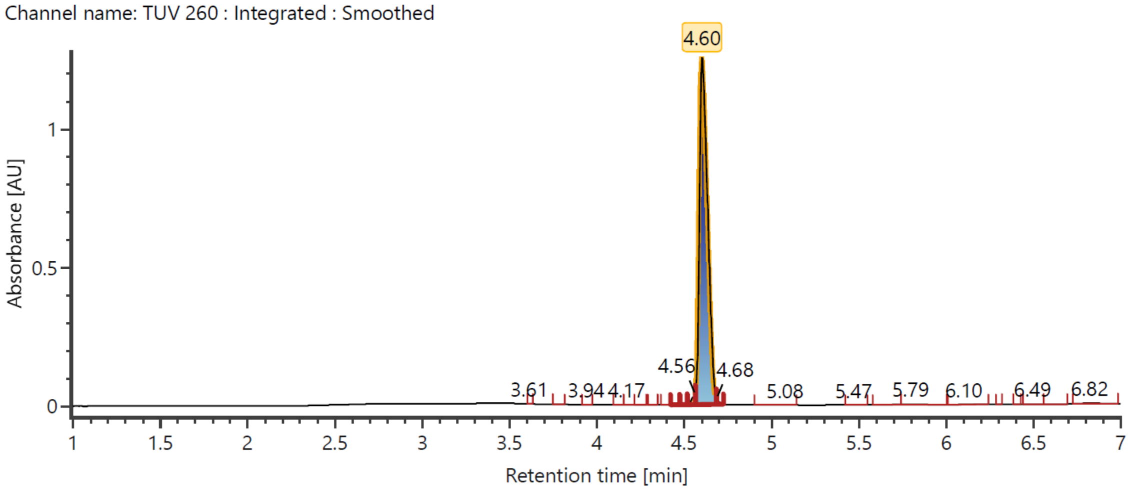


| . | Retention time (min) | Area | % Area (%) | Height |
|---|---|---|---|---|
| 1 | 4.60 | 4109558 | 97.36 | 1252810 |

**Item name: EN12762-72-2**
**Channel name: 1: TOF MS (400-5000) -40V ESI- (TIC)**

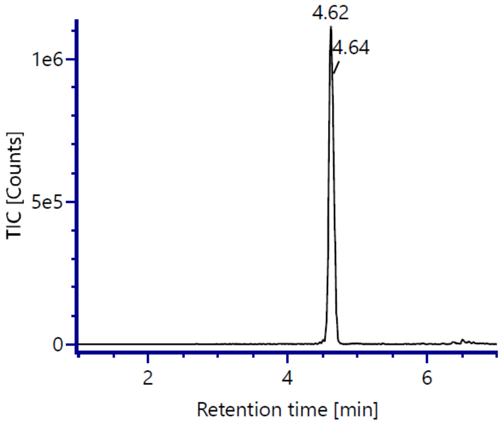


Item description:

Channel name: Time 4.6264 +/- 0.0205 minutes

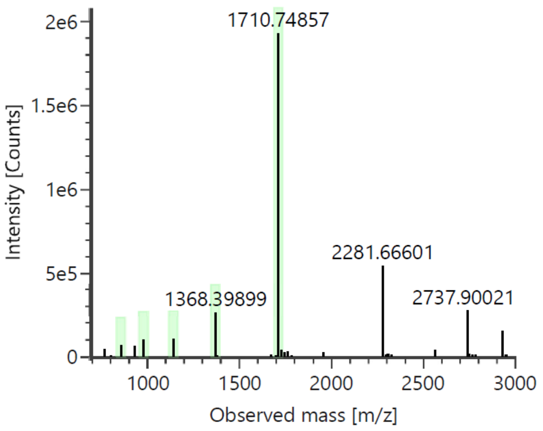

5'- rG-rA-rC-rU-rC-rC-rU-rA-rC-rA-rU-rA-rU-rU-rA-rG-rC-rA-rU-rU-rA-rA (**PA1**)

| Item name | Observed RT (min) | Neutral mass (Da) | Observed neutral mass (Da) | Observed m/z | Mass error (mDa) | Mass error (ppm) |
|---|---|---|---|---|---|---|
| EN12762-98-001 | 4.71 | 6927.94 | 6927.9161 | 1730.9717 | -26.7 | -3.9 |

Channel name: TUV 260 : Integrated : Smoothed

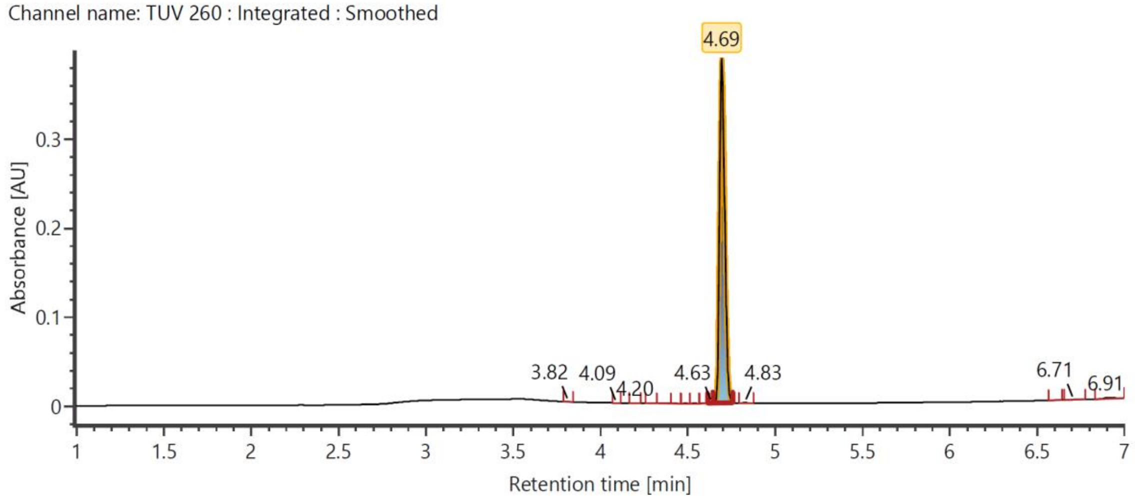


| . | Retention time (min) | Area | % Area (%) | Height |
|---|---|---|---|---|
| 1 | 4.69 | 806886 | 98.74 | 387051 |

**Item name: EN12762-98-001**
**Channel name: 1: TOF MS (400-5000) -40V ESI- (TIC)**

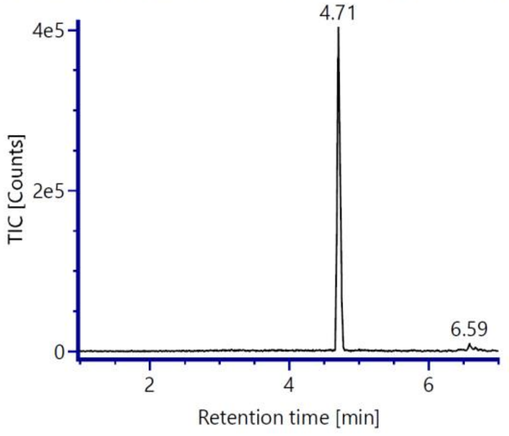


Item description:

Channel name: Time 4.7077 +/- 0.0172 minutes

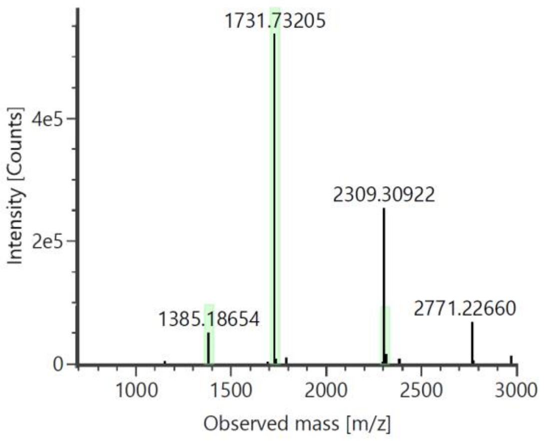

5'- mC-mU-mC-mC-mU-rA-mC-rA-mU-rA-mU-mU-rA-rG-mC-rA-mU-mU-rA-rA (**PA2**)

| Item name | Observed RT (min) | Neutral mass (Da) | Observed neutral mass (Da) | Observed m/z | Mass error (mDa) | Mass error (ppm) |
|---|---|---|---|---|---|---|
| EN12762-98-002 | 4.62 | 6422.03 | 6422.0429 | 1604.5035 | 12.3 | 1.9 |

Channel name: TUV 260 : Integrated : Smoothed

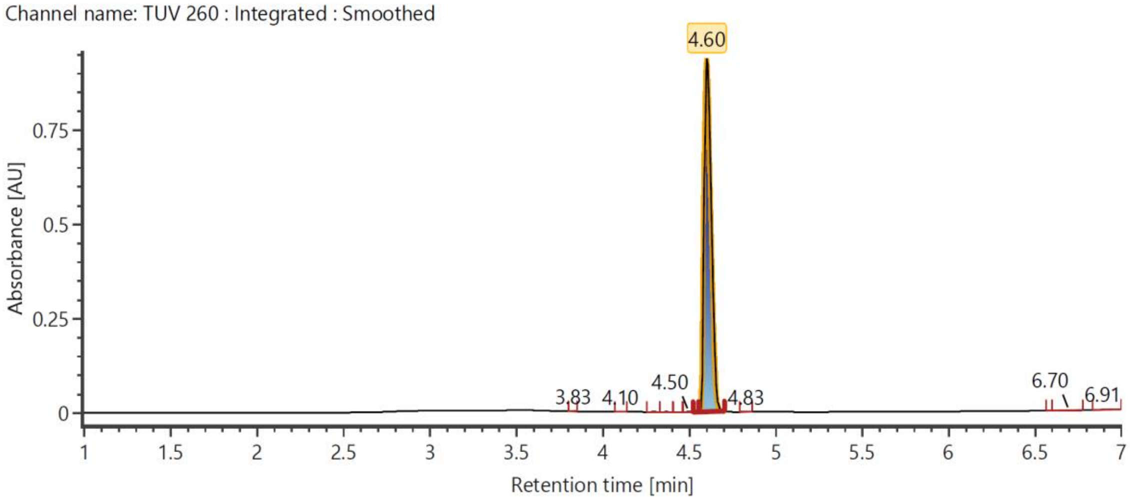


| . | Retention time (min) | Area | % Area (%) | Height |
|---|---|---|---|---|
| 1 | 4.60 | 2686360 | 99.64 | 935230 |

**Item name: EN12762-98-002**
**Channel name: 1: TOF MS (400-5000) -40V ESI- (TIC)**

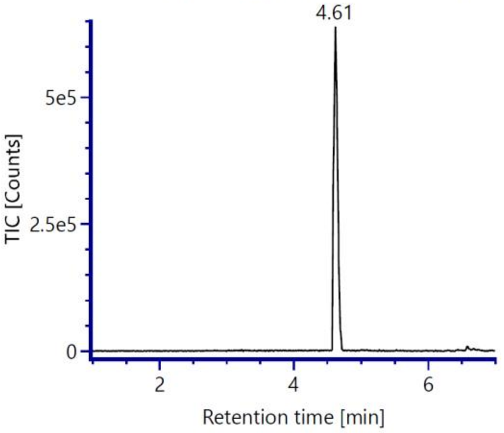


Item description: Channel name: Time 4.6186 +/- 0.0211 minutes

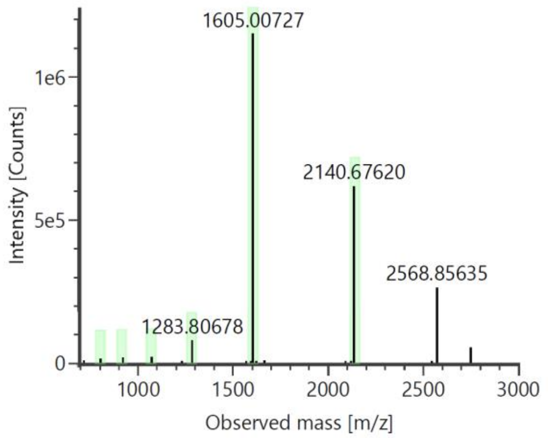

5'- mC*mU*mC-mC-mU-mA-fC-mA-fU-fA-mU-mU-mA-mG-mC-mA-mU-mU-mA-mA (**PA3**)

| Item name | Observed RT (min) | Neutral mass (Da) | Observed neutral mass (Da) | Observed m/z | Mass error (mDa) | Mass error (ppm) |
|---|---|---|---|---|---|---|
| EN12762-98-003 | 4.65 | 6530.05 | 6530.0369 | 1631.5019 | -13.3 | -2.0 |

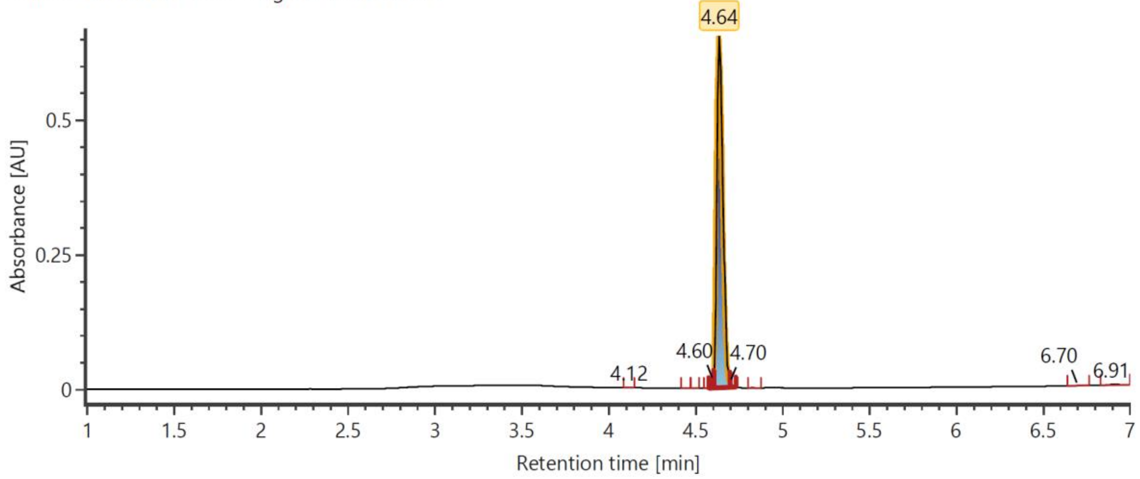


| . | Retention time (min) | Area | % Area (%) | Height |
|---|---|---|---|---|
| 1 | 4.64 | 1608781 | 98.83 | 652092 |

**Item name: EN12762-98-003**
**Channel name: 1: TOF MS (400-5000) -40V ESI- (TIC)**

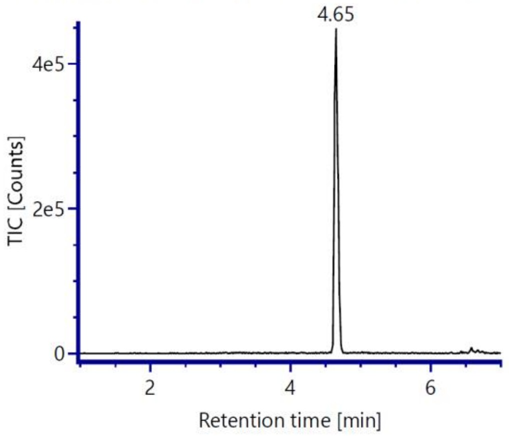


Item description: Channel name: Time 4.6500 +/- 0.0171 minutes

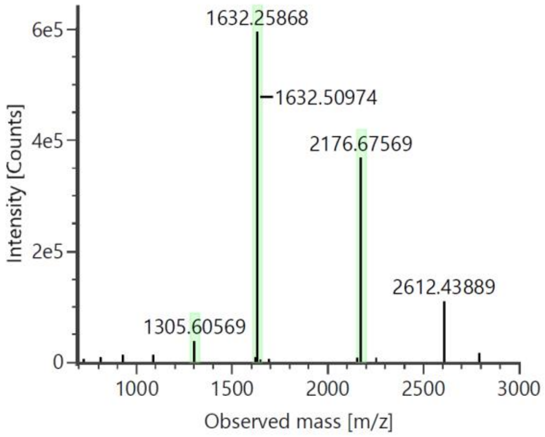

5’- rG-rC-rA-rA-rU-rC-rA-rG-rC-rA-rA-rG-rU-rA-rU-rA-rC-rU-rG-rC-rC-rC (**PB1**)

| Item name | Observed RT (min) | Neutral mass (Da) | Observed neutral mass (Da) | Observed m/z | Mass error (mDa) | Mass error (ppm) |
|---|---|---|---|---|---|---|
| EN12762-96-001 | 4.61 | 6980.99 | 6980.9584 | 1744.2323 | -33.5 | -4.8 |

Channel name: TUV 260 : Integrated : Smoothed

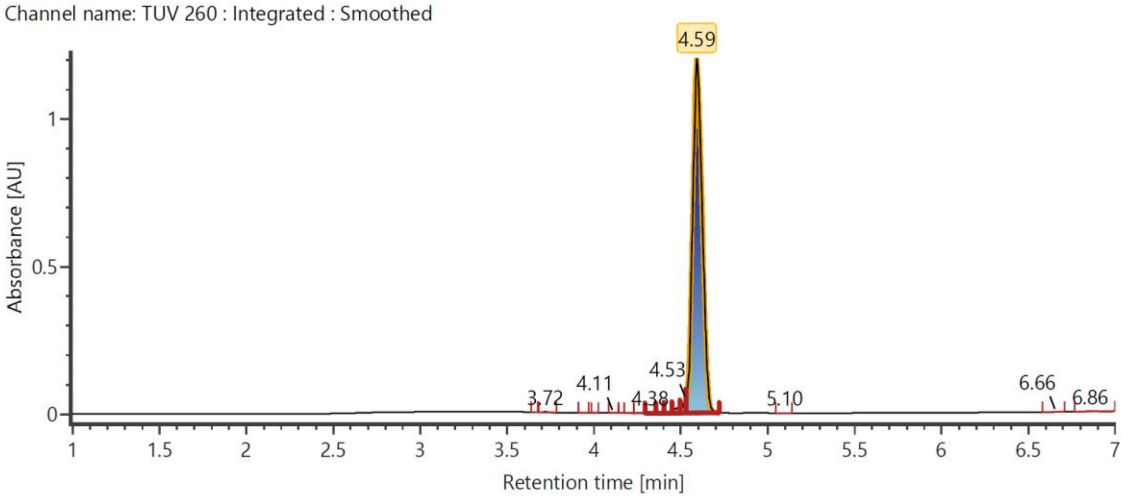


| . | Retention time (min) | Area | % Area (%) | Height |
|---|---|---|---|---|
| 1 | 4.59 | 4617550 | 97.75 | 1200054 |

**Item name: EN12762-96-001**
**Channel name: 1: TOF MS (400-5000) -40V ESI- (TIC)**

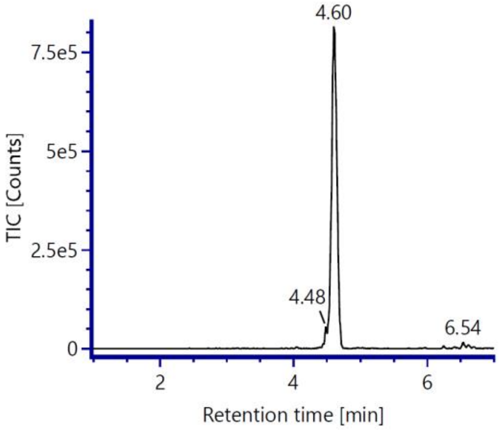


Item description: Channel name: Time 4.6064 +/- 0.0171 minutes

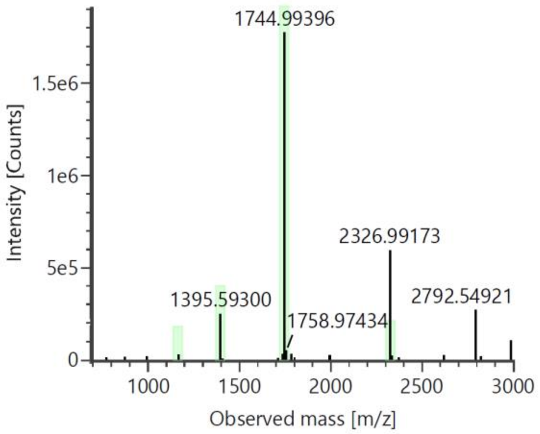

5'- rA-rA-mU-mC-rA-rG-mC-rA-rA-rG-mU-rA-mU-rA-mC-mU-rG-mC-mC-mC (**PB2**)

| Item name | Observed RT (min) | Neutral mass (Da) | Observed neutral mass (Da) | Observed m/z | Mass error (mDa) | Mass error (ppm) |
|---|---|---|---|---|---|---|
| EN12762-96-002 | 4.54 | 6471.06 | 6471.0451 | 1616.7540 | -14.5 | -2.2 |

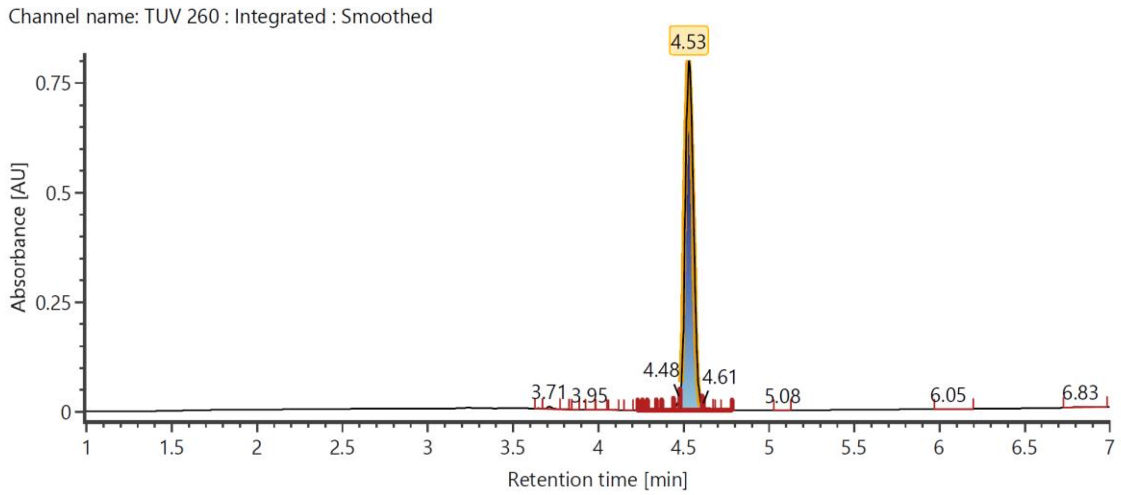


| . | Retention time (min) | Area | % Area (%) | Height |
|---|---|---|---|---|
| 1 | 4.53 | 2694717 | 97.19 | 797002 |

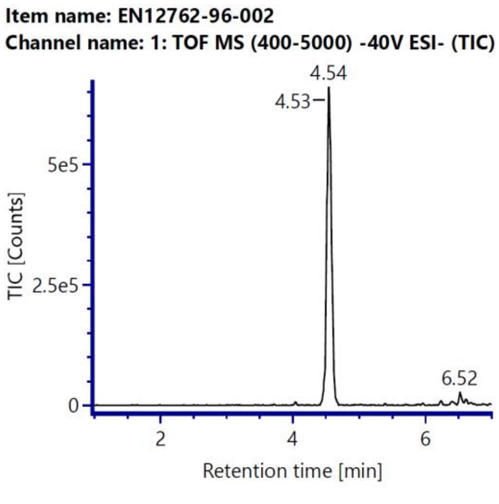


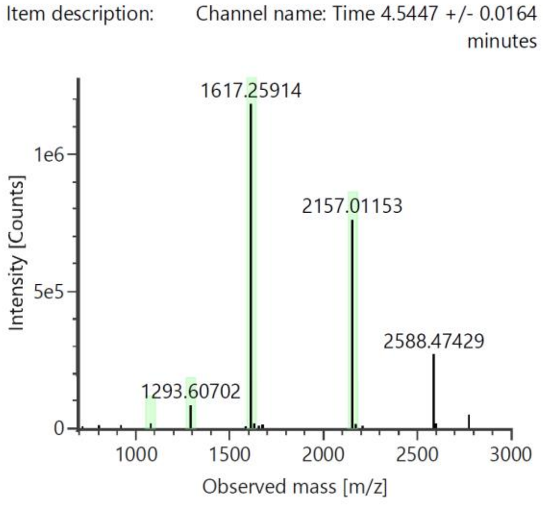

5’- mA*mA*mU-mC-mA-mG-fC-fA-fA-fG-mU-mA-mU-mA-mC-mU-mG-mC-mC-mC (**PB3**)

| Item name | Observed RT (min) | Neutral mass (Da) | Observed neutral mass (Da) | Observed m/z | Mass error (mDa) | Mass error (ppm) |
|---|---|---|---|---|---|---|
| EN12762-96-003 | 4.57 | 6595.09 | 6595.1107 | 1647.7704 | 20.2 | 3.1 |

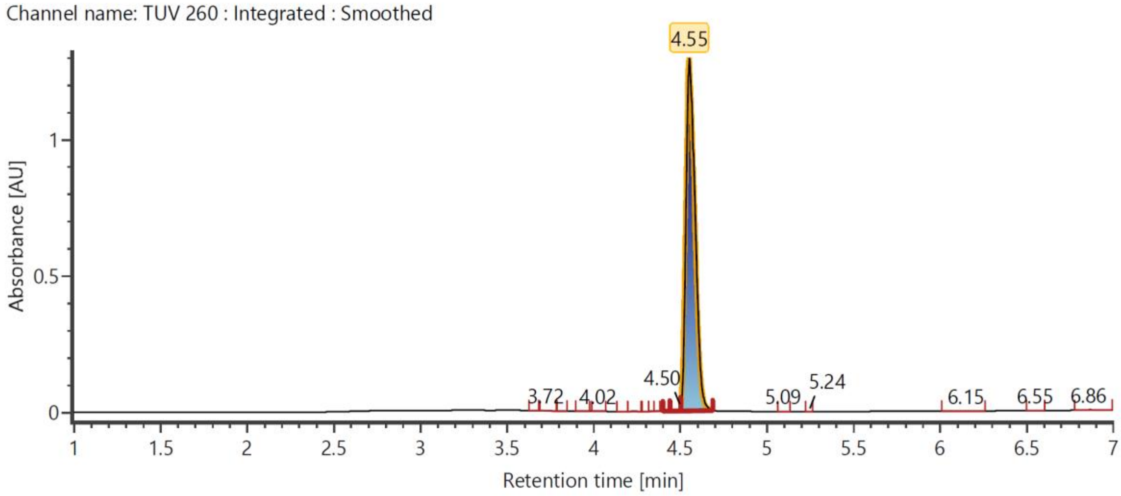


| . | Retention time (min) | Area | % Area (%) | Height |
|---|---|---|---|---|
| 1 | 4.55 | 4724944 | 99.44 | 1293199 |

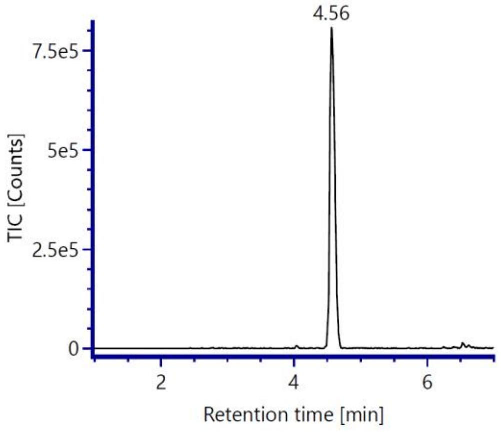


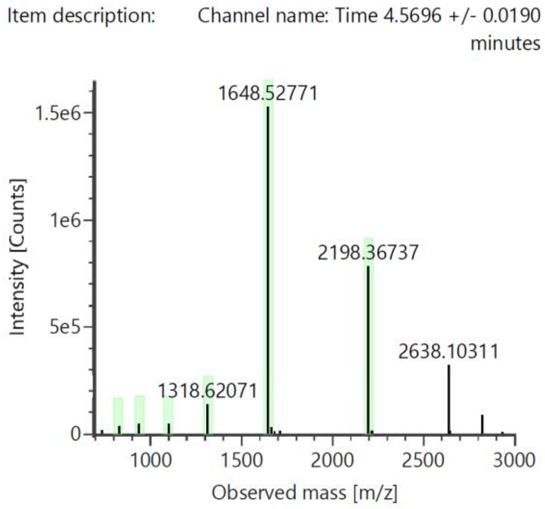

**References**


1. miRNET. https://www.mirnet.ca.
2. miRDB. http://mirdb.org.
3. miRTarbase. http://miRTarBase.cuhk.edu.cn/.
4. ExpressionAtlas. https://www.ebi.ac.uk/gxa/experiments/E-MTAB-4729/Results.
5. StringDB. https://string-db.org/.


**Full references from the main text:**


Pecot, C.V., Rupaimoole, R., Yang, D., Akbani, R., Ivan, C., Lu, C., Wu, S., Han, H.-D., Shah, M.Y., Rodriguez-Aguayo, C., Bottsford-Miller, J., Liu, Y., Kim, S. B., Unruh, A., Gonzalez-Villasana, V., Huang, L., Zand, B., Moreno-Smith, M., Mangala L. S., Taylor, M., Dalton, H. J., Sehgal, V., Wen, Y., Kang, Y., Baggerly, K. A., Lee, J-S., Ram, P. T., Ravoori, M. K., Kundra, V., Zhang, X., Ali-Fehmi, R., Gonzalez-Angulo, A-M., Massion, P. P., Calin, G. A., Lopez-Berestein, G., Zhang, W. and Soo, A. K. (2013) Tumour angiogenesis regulation by the miR-200 family. *Nature Communications*, **4**.

Chioccioli, M., Roy, S., Newell, R., Pestano, L., Dickinson, B., Rigby, K., Herazo-Maya, J., Jenkins, G., Ian, S., Saini, G., Johnson, S. R., Braybrooke, R., Yu, G., Sauler, M., Ahangari, F., Ding, S., DeIuliis, J., Aurelien, N., Montgomery, R. L. and Kaminski, N. (2022) A lung targeted miR-29 mimic as a therapy for pulmonary fibrosis. *eBioMedicine*, **85**, 104304.

Hassler, M.R., Turanov, A.A., Alterman, J.F., Haraszti, R.A., Coles, A.H., Osborn, M.F., Echeverria, D., Nikan, M., Salomon, W.E., Roux, L., Godinho, B. M. D. C., Davis, S. M., Morrissey, D. V., Zamore, P.D., Karumanchi, S. A., Moore, M. J., Aronin, N. and Khvorova, A. (2018) Comparison of partially and fully chemically-modified siRNA in conjugate-mediated delivery in vivo. *Nucleic Acids Res.*, **46**, 2185-2196.
